# *Gaultheria* Berries as Potential Functional Foods: Phytochemical Composition, Health-Promoting Properties, and Nutritional Potential

**DOI:** 10.3390/nu18183015

**Published:** 2026-09-15

**Authors:** Piotr Michel

**Affiliations:** Department of Pharmacognosy, Faculty of Pharmacy, Medical University of Lodz, Muszyńskiego 1, 90-151 Lodz, Poland; piotr.michel@umed.lodz.pl; Tel.: +48-426779169

**Keywords:** *Gaultheria* fruits, *Gaultheria* berries, phytochemical composition, polyphenols, biological activity, nutritional potential

## Abstract

Background/Objectives: *Gaultheria* L. (Ericaceae) berries are edible wild fruits traditionally consumed in several regions of Asia and the Americas and used in folk medicine for the management of fever, pain, inflammation, and digestive disorders. In recent years, they have attracted increasing attention as prospective sources of phytochemicals with potential biological activity and as candidate ingredients for future food applications. This review aimed to critically evaluate the current knowledge on the nutritional value, phytochemical composition, biological activities, and functional food potential of *Gaultheria* fruits. Methods: A comprehensive literature review was conducted using major scientific databases (Web of Science, Scopus, PubMed, Google Scholar, and ScienceDirect) to identify studies investigating the chemical composition, nutritional constituents, biological activities, and potential applications of *Gaultheria* berries. Published evidence from phytochemical analyses, cell-free assays, cellular models, and available nutritional studies was critically assessed and comparatively summarised across different *Gaultheria* species. Results: Available evidence indicates that *Gaultheria* berries are rich in polyphenols, including anthocyanins, proanthocyanidins, flavonoids, hydroxycinnamic acid derivatives, and chlorogenic acid isomers, together with characteristic salicylate glycosides, iridoids, volatile methyl salicylate derivatives, vitamins, and mineral elements. Experimental studies, predominantly performed in vitro using chemical and cell-based models, provide preliminary evidence of antioxidant, anti-inflammatory, antimicrobial, anti-diabetic, neuroprotective, photoprotective, cytoprotective, antiproliferative, dermatological, and enzyme-inhibitory activities. Despite these promising findings, comparative investigations across species remain limited, and evidence regarding bioavailability, metabolism, mechanisms of action, and clinical efficacy remains insufficient. Conclusions: Current evidence suggests that *Gaultheria* berries represent underutilised sources of structurally diverse bioactive compounds that warrant further investigation as potential food and nutraceutical ingredients. However, their health-promoting effects in humans remain unestablished. Future research should prioritise comprehensive metabolomic profiling, standardisation of phytochemical composition, bioavailability and safety assessment, in vivo validation, and controlled human studies before specific health benefits or therapeutic applications can be substantiated.

## 1. Introduction

The berry-type fruits have long been valued in traditional medicine systems across Europe, Asia, and the Americas, where different species have been used not only as staple foods but also as remedies for fever, colds, urinary-tract and bladder problems, digestive complaints, infections, and wound healing [1,2,3]. These applications anticipated what is now well documented in experimental and clinical research: berry fruits are exceptionally rich sources of vitamins, minerals, dietary fibre, and diverse phytochemicals, particularly polyphenols such as anthocyanins, flavonoids, proanthocyanidins, and phenolic acids, which together confer potent antioxidant and anti-inflammatory activities [2,4]. Regular consumption of anthocyanin-rich berries has been associated in prospective cohorts and randomised controlled trials with improved cardiometabolic health, including reduced risk of cardiovascular disease and type 2 diabetes, better lipid profiles, improved vascular function and blood pressure control, and more favourable markers of glucose homeostasis and insulin sensitivity [5,6,7]. In addition, berry-derived polyphenols have been shown in vitro and in vivo to modulate oxidative stress, endothelial function, gut microbiota, neuroinflammation, and neuronal signalling, suggesting potential neuroprotective effects and benefits for healthy ageing [8,9,10]. As a result, berries are now widely regarded as “functional” or “superfoods”, where their dense content of bioactive compounds complements their relatively low energy density and high fibre content, making them significant contributors to a health-promoting diet and attractive candidates for nutraceutical and functional-food development [3,11]. In this context, attention has recently focused on berries of the Ericaceae family, many of which have unique phenolic profiles and a long history of cultural and medicinal use among indigenous communities [12,13,14]. Among them, fruits of the genus *Gaultheria* L. stand out for their distinctive chemical composition and emerging evidence of biological activity, positioning them as underexplored sources of phytochemicals with potentially relevant biological properties.

**Figure 1:**
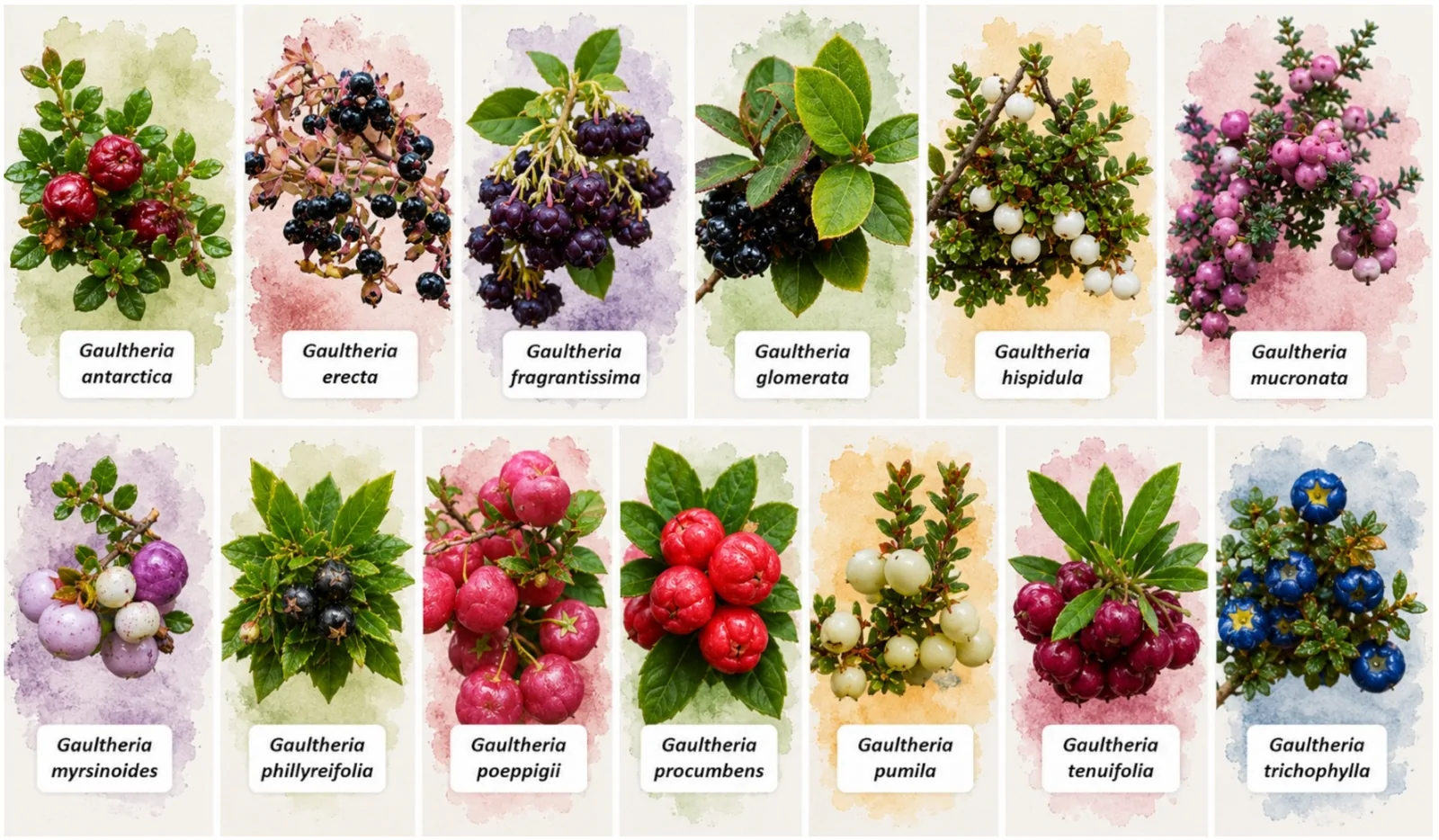
The appearance of selected *Gaultheria* fruits (Original drawings prepared by the Author based on photographic reference materials; all illustrations were completely redrawn and substantially modified from the reference images).

The genus *Gaultheria* L. (Ericaceae) comprises more than 200 species distributed across temperate and montane regions of North and South America, Asia, Australia, and New Zealand, with the greatest diversity occurring in Andean and Himalayan ecosystems [15,16,17]. Many species produce edible berries, making the genus an important natural source of wild fruits for local populations [16]. The fruits are typically small, berry-like structures botanically classified as capsules enclosed by a fleshy, enlarged fruiting calyx, and they vary in colour from white and pink to bright red, purple, or dark blue, with diameters generally ranging from a few millimetres to over one centimetre, reflecting considerable interspecific diversity as summarised in Table 1 and shown in Figure 1. Traditional medicinal use of *Gaultheria* plants is well documented in ethnobotanical records, particularly among Indigenous peoples of North and South America, where leaves and fruits have been used to treat fever, inflammation, rheumatic pain, headaches, colds, digestive ailments, and infections [13,14,15,17,18,19,20]. Phytochemical research has revealed that *Gaultheria* species accumulate a broad spectrum of bioactive compounds, including polyphenols such as flavonoids, anthocyanins, proanthocyanidins, caffeoylquinic acids, and other phenolic acids, as well as salicylate glycosides, iridoids, and, in some species, volatile methyl salicylate derivatives [15,16,17,21]. Correspondingly, extracts from *Gaultheria* fruits have shown antioxidant, anti-inflammatory, antimicrobial, antidiabetic, neuroprotective, and cytoprotective activities, predominantly in cell-free and cellular models [15,16,17,22]. These findings support further investigation of *Gaultheria* berries as candidate sources of bioactive compounds and potential food ingredients; however, their physiological and therapeutic relevance remains to be established in appropriate in vivo and human studies.

Given the growing scientific and commercial interest in the functional and bioactive properties of berry fruits and the limited, fragmented literature on *Gaultheria* species, this review provides a concise, critical overview of the phytochemical composition and biological potential of *Gaultheria* berries. It summarises and integrates current evidence on their phytochemical constituents, with particular emphasis on polyphenols and other specialised metabolites that may contribute to the biological activities observed experimentally.

Furthermore, this review critically evaluates findings from in vitro non-cellular and cell-based studies, highlighting the antioxidant, anti-inflammatory, antidiabetic, and neuroprotective activities associated with these fruits. By comparing evidence across multiple species and experimental approaches, this work identifies key patterns, evaluates current knowledge, and highlights important research gaps. It also outlines priorities for future phytochemical and nutritional research, with particular emphasis on the evidence needed to evaluate whether *Gaultheria* berries can be developed into safe, scientifically substantiated food or nutraceutical ingredients.

## 2. Methodology—Literature Search Strategy

A comprehensive literature search identified publications on the nutritional composition, phytochemical profile, traditional uses, biological activities, safety, and potential food-related applications of *Gaultheria* fruits. The electronic databases Web of Science, Scopus, PubMed, ScienceDirect, and Google Scholar were searched for publications covering the period from 1986 to 2026. The final literature search was conducted on 25 July 2026.

To maximise the coverage of the available literature, a combination of general and specific search terms was used. The principal search string was based on combinations of the following terms using the Boolean operators AND and OR: “*Gaultheria*” OR “*Gaultheria* fruits” OR “*Gaultheria* berries” AND (“phytochemical composition” OR “phenolic compounds” OR “polyphenols” OR “anthocyanins” OR “flavonoids” OR “phenolic acids” OR “salicylates” OR “salicylate glycosides” OR “iridoids” OR “terpenoids” OR “nutritional composition” OR “proximate composition” OR “minerals” OR “vitamin C” OR “traditional use” OR “ethnobotany” OR “antioxidant” OR “anti-inflammatory” OR “antidiabetic” OR “neuroprotective” OR “antimicrobial” OR “biological activity” OR “bioavailability” OR “bioaccessibility” OR “toxicity” OR “safety” OR “functional food” OR “nutraceutical” OR “food application”).

In addition, species-specific searches were performed by combining the names of individual *Gaultheria* species identified during the initial screening with the terms listed above. The reference lists of relevant publications were also screened manually to identify additional eligible studies that were not retrieved during the electronic database searches. The taxonomic nomenclature of the included *Gaultheria* species was verified according to the World Flora Online Plant List.

Following the initial search, duplicate records were removed. The remaining records were screened by title and abstract, followed, when necessary, by full-text assessment. Publications were considered eligible when they contained data specifically related to fruits, berries, fruit extracts, or fruit-derived constituents of *Gaultheria* species. Studies concerning other plant organs, including leaves, stems, roots, or essential oils obtained exclusively from non-fruit material, were excluded from the main analysis, unless they provided essential contextual information directly relevant to the interpretation of fruit composition, traditional use, safety, or biological activity.

Review articles and relevant books were not considered primary sources for the extraction of quantitative phytochemical, nutritional, or biological data; however, selected secondary sources were consulted to provide botanical, ethnobotanical, methodological, safety, and regulatory context and to identify additional primary publications through reference-list screening.

**Figure 2:**
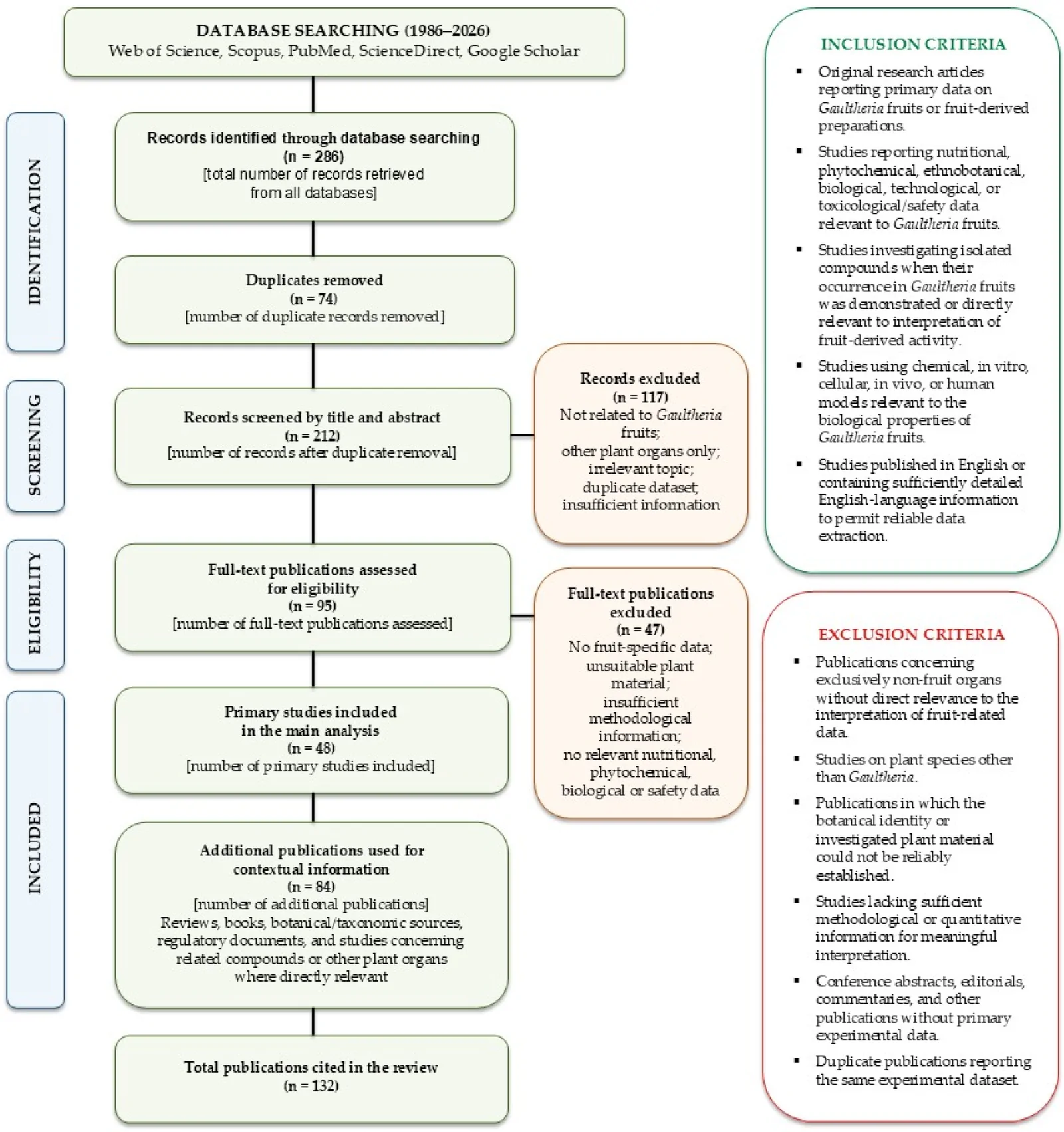
Flowchart of the literature search and study-selection process for the analysis of *Gaultheria* fruits.

Although the present work does not follow the formal framework of a systematic review, a structured literature-search and study-selection strategy was applied to increase the transparency, reproducibility, and comprehensiveness of the review. The literature-selection process, including identification, duplicate removal, title/abstract screening, full-text assessment, and final inclusion, is summarised in Figure 2.

## 3. Results and Discussion

### 3.1. Traditional Application

Traditional knowledge of *Gaultheria* fruit use is deeply rooted in the medicinal and dietary practices of Indigenous and local communities throughout North and South America, as well as in regions of Asia where the genus naturally occurs. Beyond their role as seasonal food sources, these berries have historically been used to treat fever, headache, rheumatism, inflammatory conditions, digestive discomfort, and respiratory ailments, often consumed fresh, brewed into infusions, or incorporated into poultices and tonics [13,15,16]. In several cultures, the fruits also served as a source of energy during winter months, and in some species, their characteristic aroma and flavour made them valuable as condiments or ceremonial foods [14,17].

Although the extent of ethnobotanical documentation varies among species and geographic regions, the recurrent association of *Gaultheria* fruits with anti-inflammatory applications is consistent across multiple cultural contexts. This traditional use aligns with contemporary phytochemical findings showing that *Gaultheria* fruits contain methyl salicylate derivatives, proanthocyanidins, flavonoids, and phenolic acids, compounds known to modulate inflammatory pathways and oxidative stress [15,16,17,21]. Collectively, these converging ethnobotanical and phytochemical data support the relevance of *Gaultheria* fruits as historically important food and medicinal resources containing phytochemicals with experimentally demonstrated biological activities.

#### 3.1.1. Ethnopharmacological Use

Ethnopharmacological reports document the use of *Gaultheria* fruits to manage conditions associated with pain, fever, inflammation, rheumatism, and respiratory discomfort, practices that parallel the salicylate-rich chemistry characteristic of many species [13,15,17]. In North American traditional medicine, small quantities of wintergreen-flavoured berries of *G. procumbens* were consumed fresh or prepared as infusions to alleviate headaches, muscular pain, cold symptoms, and digestive discomfort [13,14]. Indigenous communities in Canada and the northeastern United States also prepared poultices or crude extracts from mashed fruits for topical application to joints or swollen tissues, particularly for rheumatic complaints [14,17].

In southern Chile and Patagonia, ethnobotanical surveys report that *G. mucronata*, *G. phillyreifolia*, *G. poeppigii*, and *G. pumila* fruits were valued both as energy-rich wild foods and as components of traditional tonics used to address stomach ailments, fatigue, and convalescence following illness [18,19,20]. People occasionally consumed fruit-based beverages, either fermented or sweetened, as restorative preparations, while decoctions combining fruits and leaves were used for digestive inflammation and febrile conditions [36]. These traditional uses align with contemporary phytochemical and pharmacological findings showing that *Gaultheria* fruits contain polyphenols, including salicylate derivatives with documented antioxidant, anti-inflammatory, and cytoprotective activities [15,16,17]. In Andean regions, people also incorporated the berries into ceremonial foods and seasonal offerings associated with harvest rituals, reflecting their broader cultural significance beyond nutritional and medicinal roles [18,20].

#### 3.1.2. Culinary Application

Beyond their medicinal relevance, *Gaultheria* fruits have historically served as important wild edible resources in regions where the genus is abundant. Ethnobotanical records indicate that species such as *G. erecta*, *G. foliolosa*, *G. insipida*, *G. phillyreifolia*, *G. poeppigii*, *G. pumila*, *G. procumbens*, and *G. shallon* were traditionally harvested for consumption either fresh or dried, and were appreciated for their sweetness, mild acidity, and, in some taxa, a characteristic wintergreen-like aroma [13,14,37,38,39,40,41]. The berries were commonly eaten raw during foraging, served as supplementary or survival foods for Indigenous communities, and incorporated into simple preparations such as porridges, preserves, compotes, and fermented beverages [14,39].

According to Cornucopia II: A Source Book of Edible Plants, fruits of the genus *Gaultheria* are generally edible but of variable and often limited culinary importance. In most species, including *G. procumbens*, people consume the fruits raw in small amounts and value them mainly for their distinctive wintergreen aroma rather than for bulk food use, using them primarily as flavouring agents in sweets or preserves. In contrast, *G. shallon* is the most significant culinary species, producing mild-flavoured fruits eaten fresh, dried, or processed into jams and jellies, and serving as a practical wild food resource. Other species, such as *G. fragrantissima*, *G. hispidula*, *G. pumila*, and *G. trichophylla*, bear edible fruits that are consumed locally or opportunistically but are regarded as minor dietary components with little role in deliberate food processing [42].

In North America, fruits of *G. procumbens* were also used to flavour teas, sweets, and confections, reflecting their natural content of methyl salicylate and related aromatic compounds [13,17,37,43]. In southern Chile and Patagonia, ethnobotanical and ethnographic studies report that *Gaultheria* berries continue to be used in household food processing, particularly for jams, syrups, liqueurs, and desserts, contributing both flavour and seasonal nutritional value [36]. These culinary traditions underscore the long-standing role of *Gaultheria* fruits as culturally significant wild foods that bridge subsistence, flavouring, and local food heritage [44,45].

Recent food science studies have evaluated the suitability of *Gaultheria* fruits for modern food formulations. Raikos et al. (2019) reported that yogurt beverages fortified with *G. shallon* berry extracts contained higher concentrations of total phenolics and anthocyanins than plain yogurt and exhibited increased antioxidant capacity as determined by FRAP assay. During refrigerated storage, anthocyanin levels declined, whereas total phenolic content and antioxidant activity were largely maintained, indicating a degree of stability of *Gaultheria*-derived compounds in dairy matrices [46]. Although commercial cultivation remains limited, the growing interest in wild berries and functional foods suggests that *Gaultheria* fruits have potential for expanded gastronomic use and value-added food development.

#### 3.1.3. Cosmetic Application

Although less documented than culinary or medicinal uses, ethnobotanical sources report occasional topical use of *Gaultheria* fruits, including crushed berries or infused preparations for skin-related purposes. These practices have been tentatively associated with the presence of salicylate derivatives, phenolic compounds, and antioxidant pigments characteristic of the genus [15,16]. Contemporary phytochemical and pharmacognostic investigations support the biochemical basis of these uses, demonstrating that *Gaultheria* fruit extracts possess notable radical-scavenging activity and contain complex phenolic profiles dominated by anthocyanins, flavonoids, phenolic acids, proanthocyanidins, and salicylate-related constituents [17,21]. These compounds are widely recognised for their relevance in dermatological research, particularly for modulating oxidative stress and inflammation, providing a mechanistic context for the occasional topical use of *Gaultheria* fruits reported in traditional knowledge systems.

Recent investigations have further examined the cosmetic relevance of *Gaultheria* fruit constituents, including reported tyrosinase-inhibitory activity, antioxidant effects, and photoprotective mechanisms that mitigate oxidative stress. Varas-Arribasplata et al. (2021) developed a cosmetic formulation incorporating an ethanolic extract of *G. glomerata* fruits as a natural pigment in lipstick, demonstrating acceptable sensory properties, physicochemical stability, microbiological safety, and an estimated shelf-life of approximately 1.5 years under accelerated and ambient storage conditions [47]. This study represents, to date, a validated example of a cosmetic product formulated with *Gaultheria* fruit extract.

Overall, available evidence indicates that *Gaultheria* fruits may be relevant for cosmetic and cosmeceutical research, although commercial applications remain limited. Further studies on standardisation, bioavailability, dermatological safety, and formulation performance are needed to clarify their practical applicability.

#### 3.1.4. Decorative Features

Beyond their culinary and medicinal value, *Gaultheria* fruits are also recognised for their ornamental appeal. Numerous species produce abundant, brightly coloured berries, commonly red, pink, violet, blue, or white, that persist on the plants throughout autumn and winter, contributing to their attractiveness in natural and cultivated landscapes [12,24,48,49]. The glossy, spherical fruits combined with evergreen foliage, particularly in species such as *G. procumbens* and *G. mucronata*, have led to their widespread use in seasonal decorations, including holiday wreaths, garlands, potted arrangements, and table ornaments [14,17,20,25,29,30,50].

Ethnobotanical and ethnographic sources further indicate that, in regions where *Gaultheria* species occur naturally, people traditionally collected branches bearing persistent berries for domestic decoration during winter and festive periods, using them as symbols of vitality and continuity during dormant seasons [14]. The visual persistence of the fruits, together with the aromatic foliage of wintergreen-scented species, has also contributed to growing horticultural interest and their incorporation into ornamental plant trade and garden design [12,48,50]. Although these uses are non-nutritional, they reinforce cultural familiarity with the genus and have indirectly supported sustained interest in *Gaultheria* as a multifunctional group of plants with edible, medicinal, and aesthetic relevance.

#### 3.1.5. Food Reservoir for Wild Animals

Fleshy fruits are common in the genus *Gaultheria* and function primarily as rewards for vertebrate consumers that also disperse seeds. The small, brightly coloured berries grow on low shrubs and groundcovers, making them accessible to birds and mammals across temperate and montane ecosystems. Although their dietary importance varies regionally, *Gaultheria* fruits often contribute seasonally when other fleshy fruits are scarce. This role has been documented in alpine systems, where ericaceous dwarf shrubs, including *Gaultheria* species, can partially compensate for midsummer shortages of fleshy fruits in lower-elevation forests [51].

The best-documented species is *G. shallon* of western North America. Forest ecology syntheses report that numerous birds and mammals eat its late-summer fruits, including band-tailed pigeons, wrentits, ruffed and spruce grouse, squirrels, chipmunks, black bears, and black-tailed deer [52]. Stomach-content analyses of introduced Norway rats on Langara Island, British Columbia, show that *G. shallon* berries occur frequently and in high volume during late spring and early summer, indicating active and opportunistic fruit consumption when berries are locally abundant [53]. In some coastal forests, blue grouse chicks and adults consume large quantities of *G. shallon* berries during mid- to late summer, suggesting local importance during periods of high energetic demand [34,52]. Invertebrate frugivory has also been documented: banana slugs consume *G. shallon* fruits, defecate intact seeds, and can act as short-distance dispersal agents, with some seeds remaining viable after gut passage [54].

In eastern North America, eastern teaberry (*G. procumbens*) produces red fruits that persist into winter [17]. According to the U.S. Forest Service Fire Effects Information System, its berries are eaten by a wide range of birds and mammals, including wild turkey, ruffed grouse, sharp-tailed grouse, northern bobwhite, ring-necked pheasant, and several small mammals [52]. These fruits generally serve as a supplementary food source during periods of low fruit availability. Direct evidence of avian consumption comes from ruffed grouse stomach analyses in Wisconsin, where *G. procumbens* berries constituted up to 90% of wintergreen material in crops [55]. Habitat studies further indicate that eastern teaberry is common in Appalachian grouse habitats [56].

Evidence from southern South America and the tropical Andes shows that *Gaultheria* fruits also support frugivore networks in montane forests. In Chile, seeds of *G. mucronata* are commonly recovered from bird and fox feces and remain viable after gut passage [57,58], while rodents disperse *Gaultheria* seeds in Peruvian montane forests [59]. Germination trials indicate that ingestion does not consistently enhance germination, implying that the primary benefit of fruit consumption is seed transport rather than scarification [58]. Reptiles may also contribute to local dispersal: in New Zealand, geckos and skinks consume *Gaultheria* fruits, pass seeds intact, and deposit them in sheltered microsites that may facilitate establishment [60]. Similar patterns occur in alpine Japan, where *G. pyroloides* fruits are consumed incidentally by Asiatic black bears in response to local fruit abundance [51]. Overall, *Gaultheria* fruits function as seasonal dietary resources for diverse vertebrates and play a consistent role in vertebrate-mediated seed dispersal across the genus’s range.

### 3.2. Nutritional Composition and Nutritional Potential

#### 3.2.1. Proximate Nutritional Composition

Available data on the proximate nutritional composition of *Gaultheria* fruits remain very limited and are currently restricted to only a few species (Table 2 and Table S1). The most comprehensive analysis has been reported for *G. glomerata* fruits, which contained 1.3–1.5% protein, 1.0–1.2% fat, and 6.2–6.3% carbohydrates, while moisture and ash contents ranged from 74.3 to 89.5% and from 1.72 to 1.88%, respectively [61]. These results indicate that fresh fruits are characterised by high water content and relatively low macronutrient proportions. For *G. trichophylla*, only total carbohydrate content has been reported, with values of approximately 0.8–1.0 mg/100 g fresh weight depending on the sampling site [62,63]. To date, the available studies summarised in Table 2 and Table S1 do not provide quantitative data on dietary fibre or energy value, and comparable complete macronutrient profiles for most other edible *Gaultheria* species are lacking. Direct nutritional comparison of *Gaultheria* fruits with well-established edible berries, such as blueberries, cranberries, strawberries, or raspberries, is therefore limited by the scarcity and heterogeneity of available compositional data, including differences in sample preparation, analytical methodology, and reporting units. Consequently, the current evidence is insufficient to determine whether *Gaultheria* fruits offer any nutritional advantages over established edible berries, and standardised comparative studies are required. Moreover, no evidence-based serving size or recommended dietary intake has yet been established for any *Gaultheria* fruit species, owing to the scarcity of compositional, dietary-exposure, and human safety data.

#### 3.2.2. Mineral Elements

Besides organic phytochemicals, which are well known for their health benefits, *Gaultheria* fruits also contain inorganic components of interest, not only from a nutritional perspective but also because they may modulate various biological activities in humans. Remarkably, while much research has focused on the polyphenolic and volatile components of *Gaultheria* berries, these have rarely received systematic attention regarding their mineralogical composition, resulting in an insufficiently detailed characterisation to date. The review describes the macroelements and microelements important for human mineral supply, including potassium, calcium, magnesium, and sodium, as well as iron, zinc, copper, and manganese. These elements are essential for human metabolism and for maintaining efficient antioxidant defence systems, as presented in Table 2 and Table S1.

For example, in *G. glomerata* fruits, atomic absorption spectroscopy revealed potassium as the predominant element (47.3 mg/100 g), followed by calcium, magnesium, sodium, iron, and zinc, highlighting their potential contribution to dietary mineral intake [71]. In Himalayan *G. trichophylla*, flame photometry confirmed the presence of sodium (1.4–1.7 mg/100 g fw) and potassium (1.9–2.2 mg/100 g fw), further supporting the role of these berries as sources of electrolytes [62,63]. Similarly, elemental analysis of *G. trichophylla* fruits showed measurable levels of seven micro- and macroelements, with the highest content observed for magnesium (4.1 mg/100 g dw) [70].

In conclusion, although the available studies indicate that *Gaultheria* fruits contain a diverse range of mineral elements, no systematic comparison across several species has been conducted. Accordingly, the published data provide an invaluable basis for assessing interspecific differences among these fruits and the nutritional and functional roles that minerals play in *Gaultheria* berries.

#### 3.2.3. Vitamin C (Ascorbic Acid) Content

Vitamin C (ascorbic acid) is an essential dietary micronutrient and antioxidant that contributes to the nutritional value of fruits and plays important roles in human redox homeostasis and immune function [99]. Quantitative data on ascorbic acid content are available for several *Gaultheria* species, as described in Table 2 and Table S1. The ascorbic acid content in fruits of the genus *Gaultheria* has been quantitatively evaluated mainly for *G. antarctica*, *G. mucronata*, *G. fragrantissima*, *G. glomerata*, *G. myrsinoides* and *G. phillyreifolia*, using different extraction systems, especially hydroalcoholic extracts, and determined by chromatographic and colorimetric techniques including HPTLC, HPLC-DAD, Tillman’s titration and spectrophotometric assays, revealing a broad range of vitamin C levels from as low as 3.1 mg/100 g fresh weight in *G. phillyreifolia* [83], to 127.4 mg/100 g fresh weight in *G. mucronata* [64,65], with intermediate values reported for *G. antarctica* (85.6 mg/100 g fw) [64,65], *G. fragrantissima* (67.6 mg/100 g fw) [69], *G. glomerata* (13.9–85.2 mg/100 g fw/dw) [72] and *G. myrsinoides* (19.0 mg/100 g fw) [78], indicating that *Gaultheria* berries represent a variable natural source of ascorbic acid that may contribute to their nutritional and antioxidant profile and warrants further investigation in the context of potential food and nutraceutical applications.

### 3.3. Phytochemical Composition

**Figure 3:**
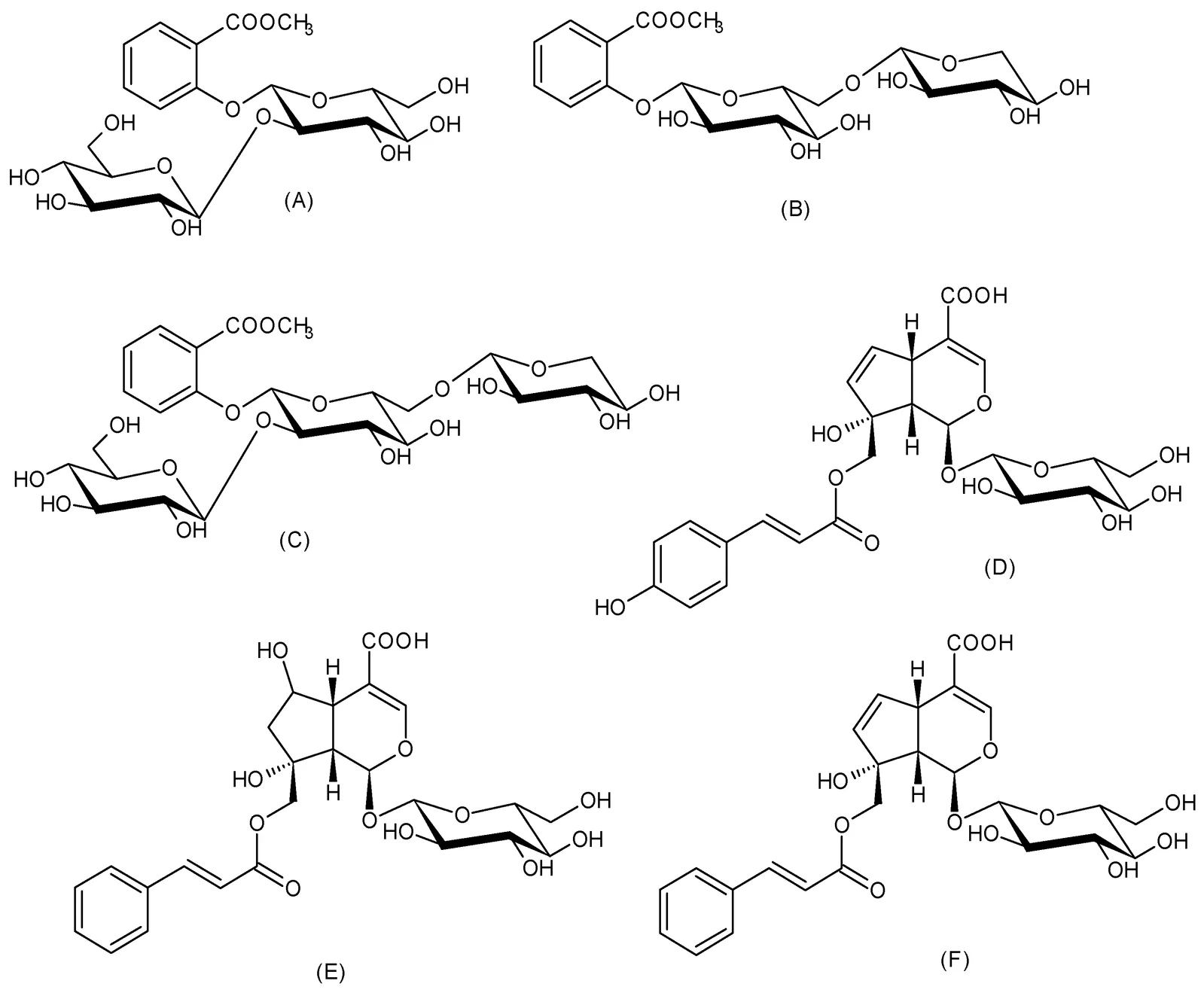
The chemical structures of: (**A**) methyl salicylate 2-*O*-β-D-glucopyranosyl-(1⟶2)-β-D-glucopyranoside (physanguloside A), (**B**) methyl salicylate 2-*O*-β-D-xylopyranosyl-(1⟶6)-β-D-glucopyranoside (gaultherin), and (**C**) methyl salicylate 2-*O*-β-D-glucopyranosyl-(1⟶2)-[*O*-β-D-xylopyranosyl-(1⟶6)]-*O*-β-D-glucopyranoside isolated from *G. procumbens* fruits [85]; and (**D**) monotropein-10-*trans*-*p*-cumarate (vaccinoside), (**E**) 6α-hydroxy-dihydromonotropein-10-*trans*-cinnamate, and (**F**) monotropein-10-*trans*-cinnamate isolated from *G. phillyreifolia* and *G. poeppigii* fruits [80].

The phytochemical profile of *Gaultheria* fruits represents one of their most distinctive and scientifically valuable features. Numerous studies indicate that these berries contain a broad spectrum of secondary metabolites, dominated by polyphenolic compounds such as anthocyanins, flavonoids, catechins (flavan-3-ols), proanthocyanidins, monocaffeoylquinic acids (chlorogenic acid isomers), and hydroxycinnamic acid derivatives, as well as characteristic constituents including salicylate glycosides, iridoids, and volatile constituents [15,16,17,21]. The polyphenolic compounds and iridoids isolated so far from *Gaultheria* fruits are shown in Figure 3. While the qualitative and quantitative composition varies across species, ripeness stages, and environmental conditions, the consistent presence of diverse phenolics is strongly associated with the antioxidant and other biological properties demonstrated for these fruits. The following sections will provide an overview of current knowledge on the main classes of bioactive compounds reported to date in *Gaultheria* fruits (as shown in Table 2 and Table S1), discussing their interspecific variability, analytical results, and the chemical complexity underlying potential health-promoting activity.

### 3.4. Phenols and Polyphenols

Phenolic compounds are among the major secondary metabolites identified in *Gaultheria* fruits and may contribute to the antioxidant, anti-inflammatory, and other biological activities observed experimentally for these fruits. This large family of compounds displays significant structural diversity, ranging from simple phenolic acids to diverse groups such as flavonoids, proanthocyanidins, catechins, and anthocyanins. Moreover, this arsenal also encompasses a wide range of hydroxycinnamic acid derivatives, including the well-known caffeoylquinic acids [15,16,17,21].

#### 3.4.1. Simple Phenolic Acids (Hydroxybenzoic and Hydroxycinnamic Acid Derivatives)

Simple phenolic acids constitute one of the principal groups of low-molecular-weight polyphenols present in fruits of the genus *Gaultheria* (Table 2 and Table S1). They occur mainly as hydroxybenzoic acid derivatives (e.g., protocatechuic, *p*-hydroxybenzoic, vanillic, and gallic acids) and hydroxycinnamic acid derivatives (e.g., caffeic, *p*-coumaric, and ferulic acids), often in free, glycosylated, or ester-bound forms. The qualitative-quantitative profile of simple phenolic acids has so far been explicitly characterised in fruits of *G. procumbens* (and, at trace/derivatised levels, in surface waxes of *G. mucronata*), where hydrophilic extracts of the berries were profiled by UHPLC-PDA-ESI-MS/MS for identification and HPLC-PDA for quantification, revealing that protocatechuic acid and *p*-hydroxybenzoic acid (hydroxybenzoic series) together with vanillic-, sinapic-, protocatechuic- and caffeic-acid derivatives (hydroxycinnamic series) dominate this class. Quantitatively, total simple phenolic acids reached their highest level in the ethyl acetate extract (6.03 mg/g dw), while individual acids ranged (mg/g dw) as follows: protocatechuic acid 0.16–0.43, *p*-hydroxybenzoic acid 0.07–0.22, and other phenolic acid derivatives (sum) trace–~2.5 depending on solvent [17,85]. Additionally, in *G. mucronata* cuticular wax (chloroform and hexane-ethyl acetate 1:1, *v*/*v*; GC-MS after BSTFA silylation), the hydroxycinnamates, i.e., *p*-coumaric acid (0.173 g/100 g dw) and ferulic acid (0.199 g/100 g dw) (total phenolic acids 0.372 g/100 g dw) predominated, underscoring solvent- and matrix-dependent enrichment [76].

This compositional pattern strongly suggested the potential of *Gaultheria* fruits to act as a rich dietary source of bioactive phenolic acids, which in turn are responsible for conferring antioxidant, anti-inflammatory, and antimicrobial properties [100]. The presence of these bioactive compounds supports further investigation of *Gaultheria* fruits as potential functional-food and nutraceutical ingredients, as well as possible sources of compounds for food, pharmaceutical, and cosmetic research. From a functional perspective, phenolic acids may also support the use of *Gaultheria* berries in food systems, including as sources of natural antioxidant or preservative compounds. However, these phytochemical characteristics alone do not establish health benefits associated with fruit consumption.

#### 3.4.2. Quinic Acid Derivatives (Including Monocaffeoylquinic Acid Isomers)

Quinic acid derivatives constitute an important subgroup of phenolic compounds in *Gaultheria* fruits, formed primarily through the esterification of hydroxycinnamic acids—most commonly caffeic, *p*-coumaric, and ferulic acids—with quinic acid. Among them, chlorogenic acid (5-*O*-caffeoylquinic acid) and its positional isomers, such as 3-*O*-caffeoylquinic acid (neochlorogenic acid) and 4-*O*-caffeoylquinic acid (cryptochlorogenic acid), are frequently reported as dominant components (Table 2 and Table S1). Across the genus *Gaultheria*, caffeoylquinic acids (chlorogenic acid isomers) have been qualitatively and quantitatively confirmed mainly in the fruits of *G. mucronata*, *G. phillyreifolia*, *G. poeppigii*, *G. procumbens*, *G. shallon*, *G. tenuifolia*, and colour morphs of *Gaultheria* spp. (pink/white), revealing chlorogenic acid and neochlorogenic acid as the dominant congeners (with occasional cryptochlorogenic acid). Their summed levels span from ~0.7–28.8 mg/100 g fw in *G. phillyreifolia* [65,80,81,82], 3.7–33.9 mg/100 g fw in *G. poeppigii* [65,80,81], 29.1–32.5 mg/100 g fw in *G. tenuifolia* [96], 4.2–42 mg/100 g dw (individual isomers) in *G. shallon* (UHPLC-MS/MS) [93,94], 8.9–19.5 mg/g dw (total) in *G. phillyreifolia* during in vitro digestion [82], and up to ~31.9 mg/g dw (total) in pink fruits versus ~10.2 mg/g dw in white fruits of *G. poeppigii* [82], while solvent fractionation in *G. procumbens* concentrates total caffeoylquinates to 0.35–0.76 mg/g dw per extract [17,85].

From a practical point of view, the high and relatively constant levels of caffeoylquinic acids highlight *Gaultheria* fruits as a naturally rich and valuable source of bioactive compounds known for their significant biological activities. Chlorogenic acid and its isomers are known to interact with pathways involved in glucose and lipid metabolism [101]. Their occurrence in *Gaultheria* berries therefore provides a phytochemical rationale for further investigation of these fruits in metabolic-health-related research; however, the effects of chlorogenic acids reported in other experimental systems cannot be directly extrapolated to consumption of *Gaultheria* berries. Additionally, these compounds contribute to several sensory properties, such as bitterness and astringency, and exhibit some antioxidant activity. *Gaultheria* fruits are thus of interest not only to the phytopharmaceutical industry, cosmetic and food technology, but also to the discovery of natural food preservatives.

#### 3.4.3. Flavonoids

Flavonoids are among the most structurally diverse and biologically significant groups of polyphenols found in *Gaultheria* fruits. They occur as both aglycones and various glycosides, most commonly *O*-glycosides conjugated to glucose, galactose, or rhamnose. Within this group, flavonols, i.e., especially quercetin, myricetin, and kaempferol derivatives, are the most frequently reported constituents. Flavonoids have been comprehensively characterised in fruits of thirteen *Gaultheria* berry species, revealing a genus-wide dominance of quercetin- and myricetin-based glycosides (galactosides, rhamnosides, and glucuronides) with lesser kaempferol and aglycones (quercetin, myricetin, and kaempferol), as described in Table 2 and Table S1. The total flavonols ranged from 46.4–163.6 mg/100 g fw (*G. phillyreifolia*) [65,80,81] to 14.6–71.0 mg/100 g fw (*G. poeppigii*) [65,80,81], 80.33–93.72 mg/100 g fw (*G. tenuifolia*) [96], 104.0 mg/g dw and 18.6–77.1 mg/g dw (non-digested *G. phillyreifolia* and *G. poeppigii*, respectively) [82], with individual leaders in *G. phillyreifolia* and *G. poeppigii* berries such as quercetin rhamnoside (up to 89.4 mg/100 g fw) [65,80,81], and solvent-fractionated *G. procumbens* showing lower totals (0.065–1.17 mg/g dw) but clear enrichment patterns [17,85].

The diversity and abundance of flavonoids increase the phytochemical interest of *Gaultheria* fruits and support further investigation of their potential as food, nutraceutical, and cosmetic ingredients. Nevertheless, their presence alone does not establish disease-preventive or therapeutic efficacy.

#### 3.4.4. Anthocyanins

Anthocyanins are key pigments responsible for the characteristic red, purple, and blue coloration of many *Gaultheria* fruits, and they represent a prominent and biologically valuable subgroup of polyphenols. These compounds occur primarily as glycosides of anthocyanidins, most commonly delphinidin, cyanidin, and malvidin, typically conjugated with glucose, galactose, or arabinose. As summarised in Table 2 and Table S1, anthocyanins are among the most intensively studied phenolic pigments in all *Gaultheria* fruits and several colour morphs (pink, white, red), revealing a consistent dominance of cyanidin- and delphinidin-based glycosides (mainly 3-*O*-galactoside, 3-*O*-arabinoside, 3-*O*-glucoside and pentosides, with minor petunidin, malvidin and peonidin derivatives). The total contents of anthocyanins were from ~0.09–1.23 μmol/g fw in *G. antarctica* and *G. mucronata* [64,65], through ~19–294 mg/100 g fw in *G. glomerata*, *G. phillyreifolia* and *G. poeppigii* [65,73,80,81], to exceptionally high levels of ~626–5942 mg CGE/100 g fw in differently coloured fruits of *G. pumila* [89]. The single pigment, i.e., cyanidin-3-galactoside (up to 158.5 mg/100 g fw), dominated the anthocyanin composition of *Gaultheria* berries [65,80,81,96].

The substantial variation in anthocyanin content among *Gaultheria* fruits indicates that genetic factors and environmental cues interact to regulate the complex pigment biosynthesis pathway. The high anthocyanin content reported in some *Gaultheria* fruits may contribute to their antioxidant properties and supports further investigation of these berries as potential sources of natural colourants and functional ingredients for food, beverage, nutraceutical, and cosmetic applications. Although anthocyanins from other dietary sources have been associated with a range of biological effects, including antioxidant, anti-inflammatory, cardiometabolic, and neuroprotective activities, such effects cannot currently be directly attributed to *Gaultheria* fruit consumption. Further bioavailability, in vivo, and human studies are required to determine whether their anthocyanin-rich profiles translate into clinically relevant health effects.

#### 3.4.5. Free Catechins (Flavan-3-ols) and Proanthocyanidins

Free catechins (flavan-3-ols) and their polymeric forms—proanthocyanidins—constitute another important group of polyphenolic compounds present in *Gaultheria* fruits, as summarised in Table 2 and Table S1. The free flavan-3-ols and their oligomeric proanthocyanidins have been documented mainly in the fruits of *G. procumbens*, *G. shallon*, *G. tenuifolia*, *G. phillyreifolia*, *G. poeppigii*, and *G. mucronata*, revealing (+)-catechin, (−)-epicatechin, gallocatechin, and epigallocatechin as the principal monomers and B-type (and occasional A-type) procyanidin dimers/trimers/tetramers as the dominant oligomers. The total proanthocyanidin contents range from ~7.41–9.59 mg/g dw in *G. procumbens* [17,85], to 52.8–280.7 mg/g dw in *G. shallon* (*n*-BuOH/HCl method) [94], 34.87–131.66 mg/100 g fw in *G. tenuifolia* (sum of catechins+proanthocyanidins, HPLC-DAD) [96], 18.2–69.3 mg CAE/g dw in *G. phillyreifolia* [65,80,81], and 25.3–68.1 mg CAE/g dw in *G. poeppigii* (DMAC assay) [65,80,81], with individual leaders such as (+)-catechin (7.75–580 mg/100 g dw), (–)-epicatechin (27–84 mg/100 g dw) and procyanidin B1/B2 (up to 76 and 55 mg/100 g dw, respectively) [94,96].

This breadth of polymerisation and the high abundance of both monomeric and polymeric flavan-3-ols highlight *Gaultheria* fruits as potentially valuable natural sources of proanthocyanidins, a class of compounds associated with diverse biological activities [102]. Their occurrence provides a phytochemical rationale for further investigation of *Gaultheria* fruits as potential ingredients in functional foods, beverages, and nutraceutical formulations. However, the biological effects attributed to proanthocyanidins in other dietary sources cannot be directly extrapolated to *Gaultheria* fruits, and their potential cardiometabolic, gut microbiota-modulating, or other health-related effects require confirmation in appropriate in vivo and human studies.

#### 3.4.6. Salicylates

Salicylates constitute a distinctive class of secondary metabolites in the genus *Gaultheria*, closely associated with the traditional medicinal use of these plants. These polyphenols have been quantitatively and structurally documented almost exclusively in the fruits of *G. procumbens*, revealing a clear dominance of the glycosidic methyl-salicylate derivatives, including gaultherin, physanguloside A, and 2-*O*-β-D-glucopyranosyl gaultherin, as mentioned in Table 2 and Table S1. The total salicylate levels reached 31.09–121.67 mg/g dw across solvent fractions (HPLC-PDA) and the individual levels spanned (mg/g dw) gaultherin 2.73–98.25 mg/g dw, physanguloside A 6.61–16.09 mg/g dw, and 2-*O*-β-D-glucopyranosyl gaultherin 2.45–27.81 mg/g dw [17,85], while free salicylic acid (after hydrolysis), analysed by GC-MS technique, occurred at ~1.491 μg/g fw [88].

The predominance of salicylate derivatives provides a plausible phytochemical basis for some of the analgesic and anti-inflammatory uses traditionally attributed to wintergreen plants [17,103]. However, the occurrence of these compounds in the fruits should not be interpreted as evidence of analgesic, anti-inflammatory, or antirheumatic efficacy after dietary consumption. *G. procumbens* berries may therefore be regarded as an interesting natural source of salicylate glycosides for further pharmacological and food-related research, while their practical use requires clarification of bioavailability, effective exposure, safety, and dose–response relationships.

#### 3.4.7. Iridoids

Iridoids represent a characteristic group of specialised metabolites in the fruits of several *Gaultheria* species and constitute one of the most distinctive phytochemical features of this genus. As summarised in Table 2 and Table S1, the qualitative and quantitative profiling of iridoids in fruits of the genus *Gaultheria* has so far been performed mainly for *G. antarctica*, *G. mucronata*, *G. phillyreifolia*, *G. poeppigii*, and *G. tenuifolia*, which revealed monotropein-type iridoids esterified with hydroxycinnamic acids as dominant constituents. The total contents of iridoids ranged from as low as 0.12–0.22 mg/g fw in mixed-colour *G. poeppigii* fruits [84], through intermediate levels of about 21.6–22.2 mg/100 g fw in *G. tenuifolia* [96], up to very high concentrations of 359.6–1334.1 mg/100 g fw in *G. phillyreifolia* [65,80,81], with monotropein-10-*trans*-cinnamate and 6α-hydroxy-dihydromonotropein-10-*trans*-cinnamate often being the quantitatively prevailing single compounds (reaching 186.5–834.5 mg/100 g fw and 118.4–452.2 mg/100 g fw, respectively) [65,80,81].

The structural diversity of iridoids and their significance in pharmacology not only reinforces the immense value of *Gaultheria* berries but also sheds light on a variety of other biological activities already reported for these compounds. The iridoid content of these berries is significant and further supports the possibility of using *Gaultheria* fruits in multiple areas, including phytotherapy, nutraceuticals, and pharmaceutical applications [104]. Moreover, it is interesting to note that iridoids, by their capacity to strengthen the plant’s defense and their implication in sensory perception (e.g., bitterness), could be of key importance for consumer acceptance and preference for these berries, also from a dietary perspective.

#### 3.4.8. Total Phenolic Contents

Total phenolic content (TPC) is an important parameter that reflects the antioxidant potential and biological activity of plant-derived raw materials, including the fruits of the genus *Gaultheria*. The spectrophotometric method using the Folin–Ciocalteu reagent is the most commonly applied assay for determining the overall phenolic content in these fruits and their extracts [105]. Researchers have evaluated TPC in fruits of several *Gaultheria* species, most commonly in alcoholic and hydroalcoholic extracts. TPC levels varied widely depending on species, extraction solvent, and expression basis, ranging from very low values of 3.28–3.71 mg GAE/100 g fw in *G. trichophylla* [62,63] through moderate levels such as 80.4 mg GAE/100 g fw in *G. fragrantissima* [69], to high concentrations of 147.5–290.3 mg GAE/g dw in *G. phillyreifolia* [65,80,81], and 189.2 mg GAE/g dw in *G. pumila* [90], with exceptionally high values reported for *G. erecta* (306.4–881.3 mg GAE/g dw) [67] and *G. glomerata* (up to 1804.07 mg GAE/100 g fw or 3503 mg GAE/100 mL extract) [71,73].

These results demonstrated that polar organic solvents such as methanol, ethanol, and their aqueous mixtures are particularly effective in extracting phenolic compounds from *Gaultheria* fruits. In contrast, less polar solvents or aqueous extracts generally yielded lower TPC values, and overall, these findings highlight that fruits of the genus *Gaultheria* represent a rich and diverse source of phenolic compounds. However, further research should focus on optimising extraction techniques, standardising phenolic profiles, evaluating bioavailability and pharmacological efficacy in vivo, and exploring sustainable cultivation and industrial processing strategies to fully exploit the therapeutic and commercial potential of these phenolic-rich fruits.

### 3.5. Terpenoids

Apart from a variety of phenolics, the fruits of species belonging to the genus *Gaultheria* contain an interesting group of terpenoids. These are predominantly represented by the volatile constituents present in the essential oils of these fruits, which not only are responsible for their characteristic and peculiar smell but also account to a great extent for their bioactivity. So far, studies on the qualitative and quantitative composition of the lipophilic fraction in *Gaultheria* fruits have mainly focused on detailed analyses of these volatile essential oil components. Notably, the volatile fractions show a high degree of species specificity, with methyl salicylate and its numerous ester derivatives emerging as the dominant components responsible for the characteristic, highly recognisable “wintergreen” smell associated with the *Gaultheria* genus. Although potentially less abundant than polyphenols, these labile terpenoids offer additional biological potential and constitute a significant phytochemical domain of *Gaultheria* berries. Recent compositional analyses of berry cuticular waxes have demonstrated that fruits of *G. mucronata* contain a diverse spectrum of lipophilic constituents, including triterpenoids such as ursolic acid (4.379 g/100 g extract), α-amyrin (1.958 g/100 g extract), and β-amyrin (0.956 g/100 g extract), as well as phytosterols and other long-chain lipid derivatives identified by GC-MS analysis. These compounds also co-occur with other lipophilic classes, including alkanes, fatty acids, alcohols, aldehydes, tocopherols, and esters, highlighting a complex non-volatile terpenoid and sterol fraction that may contribute to the protective and biological functions of *Gaultheria* fruit surfaces [76]. Nonetheless, to date, this is the only report describing the profiles of non-volatile components (triterpenes and sterols) in the fruits of species in the *Gaultheria* genus.

#### Essential Oil Components

Volatile constituents form a distinctive aspect of the phytochemical profile of *Gaultheria* fruits, contributing not only to their characteristic aroma but also to several biological properties attributed to the genus. As reported in Table 2 and Table S1, the volatile fraction of *Gaultheria* fruits has been explored mainly in *G. fragrantissima* [70], *G. mucronata* [77], *G. procumbens* [17,87], and *G. tenuifolia* [96], where essential oils obtained by hydrodistillation or headspace SPME were qualitatively and semi-quantitatively profiled by GC-MS or GC-FID-MS, revealing a chemotype strongly dominated by methyl salicylate together with variable proportions of mono- and sesquiterpenes and oxygenated aromatics, with methyl salicylate reaching 97.47% of total oil components in *G. procumbens* [17,87] and remaining a major constituent in the other species, while minor volatiles usually occur at ≤0.01–1% of the total essential oil.

The volatile composition of *Gaultheria* fruits may be technologically relevant to flavour, fragrance, and cosmetic applications [106]. Methyl salicylate and associated terpenoids have also been linked to biological activities in experimental studies; however, their occurrence in fruit-derived volatile fractions does not establish therapeutic efficacy or safety. Therefore, potential pharmaceutical or phytotherapeutic applications should be considered exploratory and require dedicated pharmacological, toxicological, and clinical evaluation.

### 3.6. Biological Activities and Critical Assessments of Evidence

The biological properties of *Gaultheria* fruits correlate well with their phytochemical variation, characterised by particular accumulations of polyphenols and unique secondary metabolites, such as salicylate glycosides and iridoids, as previously reviewed [15,16,17,21]. In the last decade, research on these important berries has expanded significantly, revealing a broad spectrum of bioactivities that not only reinforce traditional uses but also demonstrate their possible value and applicability in current health-related developments. Studies using in vitro cell-free assays and cellular models have provided preliminary evidence for a broad range of biological activities, including antioxidant, anti-inflammatory, antimicrobial, antidiabetic, neuroprotective, and cytoprotective effects across different species, as summarised in Table 3. These findings should primarily be regarded as hypothesis-generating because most have not yet been confirmed in animal models or human studies.

#### 3.6.1. Antioxidant Activity

A large and robust body of evidence shows that *Gaultheria* fruits have very high antioxidant potential. Researchers have tested this feature extensively using a broad set of complementary in vitro chemical assays and, more recently, advanced cell-based systems that confirmed the results. From this comprehensive literature, diligently compiled in Table 3, it is evident that *Gaultheria* berries show measurable but highly variable antioxidant capacity across species, extracts, processing conditions, and assay systems.

Fruits of all *Gaultheria* species have been evaluated mainly using polar extracts, with activities determined predominantly by cell-free radical/quenching and reducing assays (DPPH, ABTS/TEAC, ORAC, CUPRAC, FRAP, TBARS, O_2_^•–^, OH^•^, H_2_O_2_) and, for selected Chilean species, by cell models (AAPH-challenged Caco-2 intestinal cells, *f*MLP-stimulated human neutrophils, UVA-irradiated Hs68 dermal fibroblasts) coupled to RT-qPCR or ROS/enzyme readouts. Antioxidant capacity in *Gaultheria* fruits varies widely by species, extract type, and processing method. Crude fresh-fruit extracts from Patagonian species show modest activities (~5.5–28.9 μmol TX/g fw by TEAC/ABTS) [64,84], whereas anthocyanin-rich fractions display markedly higher values, reaching ~305–4435 μmol TX/g dw (TEAC), ~79–4968 μmol TX/g dw (ORAC), ~524–3227 μmol TX/g dw (CUPRAC), and up to ~6019 μmol TX/g dw by FRAP [80,81,84,92]. DPPH responses range from strong potencies in enriched fractions (IC_50_ ~3.6–92.8 μg/mL) to moderate inhibition in crude ethanolic extracts or fresh fruits [68,74,80,81,84]. Fruit maturity, pigmentation, and processing (fresh, frozen, or dried) influence antioxidant activity. Beyond chemical assays, bioaccessible whole-fruit extracts of *G. phillyreifolia* and *G. poeppigii* activate the Keap1-Nrf2/ARE pathway in cell models, inducing antioxidant and cytoprotective genes [83]. At the same time, related species (e.g., *G. procumbens*) suppress intracellular ROS and enhance antioxidant enzyme expression in immune and fibroblast cells [17,85,86].

The relatively high antioxidant capacity reported for several *Gaultheria* fruit extracts and anthocyanin-rich fractions supports their further investigation as sources of antioxidant phytochemicals. Cellular studies additionally provide preliminary evidence of effects on oxidative-stress-related pathways in intestinal and dermal models. However, these findings cannot currently be interpreted as evidence that consuming *Gaultheria* berries prevents metabolic disease, promotes gut health, delays skin ageing, or provides systemic antioxidant protection in humans. Further standardised in vitro studies, followed by bioavailability assessment, in vivo validation, and controlled human studies, are required to determine their physiological relevance.

#### 3.6.2. Anti-Inflammatory Activity

Anti-inflammatory activity constitutes another important pharmacological category; the use of *Gaultheria* fruits as an anti-inflammatory agent is consistent with ethnomedicinal applications and the presence of bioactive ingredients, such as salicylate glycosides and iridoids. It is also possible that the exceedingly diverse profile of activity could be explained in light of the reported anti-inflammatory effects of *Gaultheria* fruits listed in Table 3 and indicated references, mainly for *G. erecta* [67], *G. procumbens* [17,85], and *G. trichophylla* [98], where activity was found to vary appreciably with extraction solvent and assay system.

Fruit extracts of *G. erecta* showed strong anti-inflammatory potential in cell-free assays, with near-complete inhibition of collagenase, elastase, and hyaluronidase at 500 μg/mL, matching or surpassing reference standards [67]. *G. procumbens* fruit extracts exhibited moderate but consistent anti-inflammatory activity, inhibiting hyaluronidase, lipoxygenase, and COX-2, and suppressing pro-inflammatory mediator release in stimulated human neutrophils and dermal fibroblasts, including IL-1β, TNF-α, IL-8, NF-κB, and ICAM-1, alongside inhibition of Erk signalling [17,85]. Additionally, *G. trichophylla* methanolic extracts reduced protein denaturation in an in vitro inflammation model [98].

Taken together, the available in vitro findings suggest that *Gaultheria* fruit extracts may influence multiple inflammation-related targets, including iNOS, COX-2, cytokine expression, ROS generation, NF-κB, and Erk signalling. These observations provide preliminary mechanistic support for further investigation, but they do not establish in vivo anti-inflammatory efficacy. Therefore, dose standardisation, pharmacokinetic assessment, animal validation, and ultimately human studies are required before therapeutic relevance can be established.

#### 3.6.3. Photoprotective Activity

The photoprotective potential of *Gaultheria* fruits has been supported primarily by cellular studies examining UV-induced oxidative and inflammatory damage in skin cells. As summarised in Table 3, photoprotective activity has been reported (albeit only for *G. procumbens* fruits) with an acetone extract that was predominantly studied in UVA-irradiated human Hs68 dermal fibroblasts, where protection assessed was cell viability (MTT/viability assays), intracellular ROS levels, endogenous antioxidant enzyme activities, and DNA damage (comet/tail DNA) [17,86].

Pre-treatment with the fruit acetone extract significantly protected cells against UVA-induced damage by preserving viability, reducing genotoxicity, and enhancing antioxidant defences, including superoxide dismutase and glutathione *S*-transferase activities, while lowering intracellular ROS levels. Reference antioxidants (quercetin and ascorbic acid) showed comparable or stronger protective effects, supporting a mechanistic role of polyphenols and salicylate derivatives in cellular UV defence [17,86].

Taken together, these cellular findings warrant further investigation of *G. procumbens* fruit extracts as candidate ingredients for dermocosmetic formulations [17]. However, their photoprotective efficacy and safety remain to be established in appropriate skin models, in vivo studies, and formulation-specific investigations.

#### 3.6.4. Cytoprotective Activity

Some studies show that fruit extracts of the genus *Gaultheria* can protect gastrointestinal epithelial cells, which may benefit gut health and mucosal defence. The cytoprotective effects of *Gaultheria* fruits have been reported for *G. phillyreifolia* [83], *G. poeppigii* [83], and *G. tenuifolia* [96], using mostly methanol-formic acid (99:1, *v*/*v*) extracts or their anthocyanin or copigment fractions, as well as polyphenol-enriched extracts in assays based on the MTT test of cell viability from human gastric epithelial cells AGS and human intestinal Caco-2 cells, as mentioned in Table 3. In vitro studies indicate that *Gaultheria* fruit extracts exhibit low to moderate cytotoxicity. Whole extracts or fractions of *G. phillyreifolia* and *G. poeppigii* preserved ~63–82% viability in AGS cells at 50 μg/mL, while bioaccessible micellar fractions maintained ~51–58% survival in Caco-2 cells [83]. Likewise, polyphenol-, anthocyanin-, and copigment-enriched fractions from *G. tenuifolia* showed ~55–84% viability in AGS cells at 6.25–25 μg/mL [96].

Although cytoprotective effects have been shown in vitro using epithelial cell lines, not in animals or patients, these results suggest a potential application of *Gaultheria* fruits for gastrointestinal health, mucosal healing, and preventing oxidative or inflammatory injuries in the gut. Future studies should address in vivo gastric lesion models, intestinal permeability assays, gut microbiota interactions, and synergistic effects with dietary constituents. Creating bioavailability profiles and characterising active metabolites will also be essential to translate in vitro findings into useful health-promoting applications.

#### 3.6.5. Antiproliferative (Anticancer-Oriented) Activity

Antiproliferative effects of *Gaultheria* fruits have been reported in a limited number of studies, making this an emerging but promising direction for future investigation. The anticancer-oriented potential has so far been specifically evaluated only for fruits of *G. pumila*, using a methanol-formic acid (98:2, *v*/*v*) extract tested in a panel of human cancer cell lines by the sulforhodamine B (SRB) colorimetric assay, which quantifies cellular protein as a proxy for growth and viability [90]. The six tumour models were examined, i.e., A549 and SW1573 (lung), HBL-100 and T-47D (breast), HeLa (cervix), and WiDr (colon), over a concentration range of 2.5–250 μg/mL, yet the extract showed no marked cytostatic or cytotoxic effect, with both GI_50_ (50% growth inhibition) and TGI (total growth inhibition) values exceeding 250 μg/mL for all lines [90].

Although the existing data remain early-stage and mostly in vitro, the antiproliferative activities, along with the antioxidant, anti-inflammatory, and enzyme-modulating properties already described for *Gaultheria* fruits, underscore their potential as complementary anticancer candidates for drug development. Future advances in mechanistic cancer models, apoptosis pathway mapping, metastatic inhibition assays, and in vivo tumour testing will be required. Cross-species comparisons and fractionation-guided characterisation of biologically active components will be necessary to determine potency, specificity, and clinical implications. Potential interactions with classical chemotherapeutics and dietary supplements could further enhance the therapeutic potential of *Gaultheria* fruit metabolites.

#### 3.6.6. Anti-Diabetic Activity

Recent studies have highlighted *Gaultheria* fruits as potential anti-diabetic agents [111] given their richness in polyphenolics, including flavonoids, anthocyanins, proanthocyanidins, and hydroxycinnamic acid derivatives, as well as iridoids and other glycosides [22]. The anti-diabetic properties of *Gaultheria* fruits have been reported for *G. hispidula* [110], *G. phillyreifolia* [82], and *G. poeppigii* [82], mainly using ethanol-water (80:20, *v*/*v*) or methanol-formic acid (99:1, *v*/*v*) extracts assayed in cell-free α-glucosidase/α-amylase assays and in Caco-2 human intestinal epithelial cell models simulating glucose absorption.

Extracts of *G. hispidula* and *G. phillyreifolia* berries show potent inhibition of intestinal glucose handling. An ethanol-water extract of *G. hispidula* reduced ^3^H-deoxy-D-glucose uptake in Caco-2/15 cells by ~66% after 10 min [110]. Methanol-formic acid extracts of *G. phillyreifolia* strongly inhibited α-glucosidase (IC_50_ = 0.7–2.8 μg/mL) and suppressed glucose transport in digested fractions, accompanied by marked downregulation of glucose transporter and digestion genes (SGLT1, GLUT2, GLUT5, SI) [82]. Similarly, *G. poeppigii* fruit extracts inhibited α-glucosidase (IC_50_ = 2.3–24.9 μg/mL) and reduced glucose uptake in Caco-2 cells, with concomitant decreases in transporter gene expression [82].

Taken together, these findings suggest that the in vitro glucose-modulating effects of *Gaultheria* fruits are mediated by inhibition of carbohydrate-hydrolysing enzymes and down-regulation of intestinal glucose transporters. However, most evidence comes from in vitro systems, and no controlled animal or clinical studies have yet shown physiological relevance. Further studies on bioavailability, metabolite absorption, long-term metabolic effects, and synergies between fruit compounds are required to assess the full potential of *Gaultheria* berries as a natural approach for glycaemic control and type 2 diabetes prevention.

#### 3.6.7. Neuroprotective Activity

The potential relevance of *Gaultheria* fruits to neurodegeneration-related research has received limited attention to date. Fernández-Galleguillos et al. (2021) evaluated a methanol-formic acid (98:2, *v*/*v*) extract of *G. pumila* fruits in a cell-free cholinesterase-inhibition assay [90]. The extract inhibited acetylcholinesterase (AChE) with an IC_50_ of 7.7 μg/mL and butyrylcholinesterase (BChE) with an IC_50_ of 34.5 μg/mL, showing dose-dependent activity, although it was less potent than the reference drug galantamine (IC_50_ = 0.3 and 3.82 μg/mL for AChE and BChE, respectively) [90]. These findings demonstrate cholinesterase-inhibitory activity in vitro but should not, by themselves, be interpreted as evidence of neuroprotective efficacy.

The evidence supporting the potential neuroprotective activity of *Gaultheria* fruits remains at a very preliminary stage and is based primarily on cell-free enzyme-inhibition assays, with limited evidence from neuronal cell models and no validation in appropriate animal or human studies. Nevertheless, the observed cholinesterase-inhibitory activity, together with the presence of phenolic compounds with antioxidant and anti-inflammatory properties, provides a rationale for further investigation of *Gaultheria* fruit extracts in experimental models relevant to neurodegeneration. Future studies should include neuronal cell models, oxidative and inflammatory injury paradigms, identification and pharmacokinetic assessment of potentially brain-accessible metabolites, and subsequent in vivo studies before any neuroprotective or cognitive effects can be substantiated.

#### 3.6.8. Dermatological Activity

*Gaultheria* fruit extracts have shown preliminary in vitro activities of potential dermatological relevance, such as antioxidant activity, anti-inflammatory potential in the skin, and a skin-whitening effect. As shown in Table 3, the dermatological activity of *Gaultheria* fruits has been demonstrated for *G. erecta* [67] and *G. pumila* [90] mainly based on tyrosinase inhibition assays that simulate skin-whitening and anti-hyperpigmentation effects. Methanolic fruit extracts of *G. erecta* (immature, intermediate, and mature berries) inhibited mushroom tyrosinase by 90.0–93.0% at 500 μg/mL, matching or surpassing the activity of *Camellia sinensis* extract and approaching that of kojic acid [67]. In contrast, berries of *G. pumila*, extracted with methanol-formic acid (98:2, *v*/*v*), showed even stronger activity with an IC_50_ of 3.3 μg/mL, which was markedly lower than the reference kojic acid (IC_50_ = 10.0 μg/mL) [90].

Existing results largely come from in vitro experiments that modulate melanogenesis-related enzymes, offering strong prospects for further investigation. Future research opportunities include UV-exposure models, preserving the collagen matrix in wound-healing assays, and testing the dermal penetration and stability of active ingredients. With rising demand for natural cosmetic ingredients, *Gaultheria* berry extracts therefore warrant further investigation as potential botanical sources of compounds relevant to skin-lightening, anti-wrinkle, and photoprotective applications.

#### 3.6.9. Antimicrobial Activity

Fruits of some *Gaultheria* species have attracted attention in recent years for their potential antimicrobial activity, owing to bioactive phytochemicals such as essential oils, which have demonstrated antimicrobial activity in experimental studies. As summarised in Table 3, *Gaultheria* berries and their essential oils have a wide range of antimicrobial potential, such as antibacterial, antifungal, and antiparasitic (antischistosomal) activities, with this activity being attributed chiefly to *G. procumbens* [17,87], *G. trichophylla* [97], and *G. mucronata* [77].

Hydrodistilled fruit essential oil of *G. procumbens* showed antibacterial activity against both reference and clinical strains, with MICs of 8.2–16.4 mg/mL against *Staphylococcus aureus*, *Enterococcus faecalis*, *Escherichia coli*, and *Acinetobacter baumannii* [17,87]. Similarly, methanolic and solvent-partitioned fruit extracts of *G. trichophylla* inhibited *Bacillus subtilis*, *S. aureus*, *E. coli*, and *Pseudomonas aeruginosa*, with MICs ranging from 7.5 to 30 mg/mL, and exhibited moderate antifungal effects against *Helminthosporium solani*, *Fusarium solani*, *Aspergillus flavus*, and *A. fumigatus* [97]. In addition, *G. mucronata* fruit essential oil showed notable in vivo antiparasitic activity, reducing the worm burden of *Schistosoma mansoni* by 67% in mice at 5 mg/kg [77].

Overall, the available studies provide preliminary evidence of antimicrobial and antiparasitic activity in selected *Gaultheria* fruit preparations. However, the evidence remains limited to in vitro antimicrobial assays and, for antiparasitic activity, a restricted in vivo dataset. Further studies are required before applications in food preservation, topical products, or therapeutic settings can be considered.

#### 3.6.10. Critical Assessment of the Level and Strength of Evidence Supporting Biological Activities

Although numerous biological activities have been reported for *Gaultheria* fruits, their potential health relevance should be interpreted according to the experimental level at which the effects were demonstrated. In particular, results from chemical antioxidant assays or isolated enzyme systems provide useful screening information but cannot be directly extrapolated to physiological efficacy, since assay conditions, reactant concentrations, and reaction mechanisms may differ substantially from those in vivo [112]. Recent critical evaluations have emphasised that antioxidant capacity measured by DPPH, ABTS, FRAP, ORAC, or related assays does not necessarily predict biological effects in humans and should therefore be distinguished from evidence from cellular, animal, and clinical studies [113,114]. Moreover, even cell-based experiments have important translational limitations because the stability, metabolism, and effective concentrations of polyphenols under cell-culture conditions may differ markedly from their behaviour after ingestion [115]. More broadly, recent analyses of phytochemical research identify insufficient bioavailability, incomplete mechanistic validation, inadequate experimental models, and the scarcity of controlled human studies as major barriers to clinical translation [116]. Accordingly, the available evidence for the biological activities of *Gaultheria* fruits was critically classified according to the experimental model, presence of appropriate reference controls, reproducibility across species or studies, and availability of cellular, in vivo, or human confirmation (Table 4).

### 3.7. Bioavailability, Bioaccessibility and Translational Limitations

The biological relevance of phytochemicals present in *Gaultheria* fruits cannot be inferred directly from their concentrations in the raw material or from activity demonstrated in cell-free assays. After ingestion, bioactive compounds must be released from the food matrix, remain stable during gastrointestinal digestion, and be absorbed in the intestine before reaching systemic circulation. Presystemic metabolism, distribution, elimination, food-matrix interactions, and gut microbiota composition further affect bioavailability [117,118]. Many dietary phytochemicals may exhibit substantial in vitro activity despite limited or highly variable systemic exposure, while circulating metabolites rather than the parent compounds originally present in foods may ultimately determine their biological effects in vivo [118].

This issue is particularly relevant to polyphenols, which constitute one of the major groups of specialised metabolites in *Gaultheria* berries. Anthocyanins, flavonols, proanthocyanidins, and hydroxycinnamic acid derivatives differ considerably in gastrointestinal stability and absorption. A substantial proportion of ingested polyphenols reaches the colon and is transformed by the gut microbiota into lower-molecular-weight metabolites, resulting in plasma and tissue exposure that may differ markedly from the concentrations of parent compounds used in in vitro experiments [118,119]. Consequently, current approaches increasingly emphasise the need to characterise the full spectrum of absorbed metabolites rather than considering only the native phytochemicals present in fruits [118,119].

Importantly, *Gaultheria*-specific studies demonstrate that gastrointestinal digestion can markedly modify biological activity. For *G. phillyreifolia*, simulated digestion reduced α-glucosidase inhibitory potency from an IC_50_ of 0.7 μg/mL to 2.8 μg/mL, whereas for *G. poeppigii* the corresponding values changed from 2.3–3.1 μg/mL to 12.2–24.9 μg/mL. Nevertheless, bioaccessible fractions obtained after digestion remained active in Caco-2 intestinal cells, where they reduced glucose uptake and modulated the expression of SGLT1, GLUT2, GLUT5, and sucrase-isomaltase [82]. Similarly, digested fractions containing iridoids from *G. phillyreifolia* and *G. poeppigii* exhibited cytoprotective effects in gastric and intestinal epithelial-cell models [83]. However, these experiments do not provide information on systemic absorption, plasma concentrations, tissue distribution, metabolism, or elimination.

Salicylate glycosides also require cautious interpretation. *G. procumbens* fruits contain gaultherin, physanguloside A, and related methyl-salicylate glycosides in substantial amounts [17,85]. Their gastrointestinal transformation and subsequent systemic exposure may differ considerably from the concentrations of the native glycosides present in the fruit. Therefore, a high salicylate content in an extract cannot be equated directly with predictable systemic exposure or anti-inflammatory efficacy. The same limitation applies to terpenoids and other lipophilic constituents, whose oral bioavailability may be restricted by poor aqueous solubility, variable intestinal absorption, and extensive metabolism. These limitations are well recognised for plant-derived compounds, and formulation approaches aimed at improving solubilisation and intestinal absorption have consequently received considerable attention [117,120].

Overall, most antioxidant, anti-inflammatory, antidiabetic, neuroprotective, dermatological, and other effects reported for *Gaultheria* fruits have been demonstrated in chemical or cellular models. Such systems are valuable for identifying biological potential but do not reproduce gastrointestinal degradation, metabolism, plasma-protein binding, tissue distribution, or elimination. Thus, concentrations effective in vitro may not be achievable after realistic dietary consumption, and in vitro effectiveness should not be regarded as equivalent to in vivo efficacy. Poor or variable bioavailability, structural instability, incomplete mechanistic understanding, and insufficiently predictive experimental models are recognised as major barriers to successful clinical translation of phytochemicals [116].

At present, no controlled human intervention studies have established the pharmacokinetics, dose–response relationships, or clinical efficacy of *Gaultheria* berries. Future research should therefore prioritise standardised gastrointestinal digestion, identification of bioaccessible and circulating metabolites, intestinal transport and pharmacokinetic studies, followed by controlled animal and human investigations. Only such evidence will establish whether the biological activities currently observed in vitro translate into meaningful nutritional or health effects in humans [116,118].

### 3.8. Safety and Toxicological Aspects

The safety of *Gaultheria* fruits requires particular consideration because several species contain salicylate derivatives, including methyl salicylate glycosides. Although the long history of traditional use of *G. procumbens* suggests that the fruits and conventional preparations are generally well tolerated, the available toxicological evidence is insufficient to establish species-specific safe daily intake levels for *Gaultheria* berries. Moreover, safety data for whole fruits should not be extrapolated directly to concentrated extracts, salicylate-enriched fractions, supplements, or essential oils, where exposure to individual bioactive compounds may be substantially higher. Previous studies demonstrated good in vitro cytocompatibility of *G. procumbens* fruit and other plant-part extracts, including no significant reduction in human neutrophil viability at 25–150 μg/mL [17]; however, systematic in vivo toxicological and human safety studies remain scarce.

Among the constituents of greatest toxicological relevance are methyl salicylate glycosides, particularly gaultherin, which occurs at substantial levels in *G. procumbens* fruits. Gaultherin is gradually hydrolysed in the gastrointestinal tract and acts as a precursor of salicylate metabolites. Preclinical evidence suggests a more favourable gastrointestinal profile than aspirin, with gradual salicylate release and no direct COX-1 inhibition [103]. Nevertheless, these findings should not be interpreted as evidence that high dietary or supplemental exposure to *Gaultheria*-derived salicylates is risk-free. Salicylates at excessive doses can produce systemic toxicity characterised by gastrointestinal symptoms, tinnitus, acid–base disturbances, neurological manifestations, seizures, pulmonary oedema, and, in severe cases, coma [121]. Furthermore, dietary salicylates may be relevant to susceptible individuals and can interact with non-steroidal anti-inflammatory drugs and anticoagulants such as warfarin [122]. Therefore, particular caution is warranted in individuals with salicylate or aspirin hypersensitivity, including those with aspirin/NSAID-exacerbated respiratory disease [123].

Importantly, the toxicological profile of whole berries and glycosidically bound salicylates must be clearly distinguished from that of wintergreen essential oil, which may contain approximately 97–100% methyl salicylate [17]. Accidental or intentional ingestion of wintergreen oil has caused severe and sometimes fatal salicylate poisoning, with reported manifestations including vomiting, tinnitus, tachypnoea, seizures, altered consciousness, and cardiovascular complications [17]. Consequently, toxicological evidence for concentrated wintergreen oil should not be used to imply equivalent toxicity of normal dietary consumption of *Gaultheria* berries; rather, it illustrates the potential hazard associated with concentrating their methyl salicylate-related constituents.

Natural salicylate glycosides may nevertheless differ substantially from free methyl salicylate in their pharmacokinetic and safety profiles. Gaultherin undergoes gastrointestinal biotransformation and has shown favourable intestinal absorption and no gastric ulcerogenicity in preclinical studies [103]. Similar observations have been reported for salicin-containing preparations, which are generally well tolerated, although mild gastrointestinal symptoms and occasional allergic reactions have been reported, particularly in salicylate-sensitive individuals [124]. These observations support the concept that the chemical form of the salicylate strongly influences exposure and toxicity, but evidence obtained for salicin or gaultherin cannot yet define a safe intake level for *Gaultheria* fruits.

No major safety signals have been documented in relation to traditional dietary use of *Gaultheria* berries; however, the available evidence is insufficient to establish their safety quantitatively or to define species-specific safe intake levels. Species-specific variation in salicylate composition and the lack of controlled human safety studies further limit risk assessment. Future studies should therefore determine salicylate exposure from realistic serving sizes, establish acute and repeated-dose toxicity of standardised fruit preparations, evaluate potential drug–phytochemical interactions and hypersensitivity, and define tolerable intake levels for both healthy and susceptible populations. Until such evidence becomes available, assess the safety of whole berries, concentrated extracts, supplements, and methyl salicylate-rich essential oils separately rather than extrapolating from one product category to another.

### 3.9. Processing, Storage and Technological Aspects

Processing and storage conditions critically determine the phytochemical quality of *Gaultheria* berries and their suitability for functional-food applications. This is particularly relevant to anthocyanins, other polyphenols, and volatile constituents, which may degrade substantially during thermal treatment, drying, fermentation, and prolonged storage. Anthocyanins are especially sensitive to elevated temperature, oxygen, light, pH, and interactions with other food components, and their degradation may cause simultaneous loss of colour and biological functionality [125,126].

Although *Gaultheria*-specific processing studies remain scarce, available evidence confirms that preservation methods can substantially modify fruit phytochemistry. In *G. glomerata*, fresh, frozen, and dried fruits differed considerably in total phenolic and anthocyanin contents, indicating that post-harvest treatment is an important source of variability in the chemical composition of these berries [73]. Similarly, incorporating *G. shallon* berries into a yogurt beverage showed that total phenolic content and antioxidant capacity were relatively well maintained during refrigerated storage, whereas anthocyanin concentrations progressively declined [46]. These observations are consistent with studies of other anthocyanin-rich berries showing that freezing generally preserves phenolic compounds more effectively than conventional thermal drying, while prolonged heating can accelerate anthocyanin degradation. Freeze-drying is consequently regarded as one of the most effective approaches for retaining berry polyphenols, although its industrial application is limited by relatively high processing costs [125].

Fermentation is a more complex process because it may decrease native phenolic compounds or generate metabolites with different bioaccessibility and biological properties. Accordingly, fermented juices, wines, and other *Gaultheria*-based products should not be assumed to have the same phytochemical profile as fresh berries. Similar technological considerations apply to essential oils and other volatile fractions. Heating and drying may promote evaporation, oxidation, or transformation of volatile constituents, potentially modifying the characteristic wintergreen aroma and methyl salicylate-related profile. This may be particularly relevant for species such as *G. procumbens*, whose fruit essential oil is strongly dominated by methyl salicylate [17,87].

From a technological perspective, prioritise mild processing, rapid freezing, freeze-drying, controlled fermentation, protection from oxygen and light, and appropriate low-temperature storage when developing *Gaultheria*-based products. Encapsulation and protective food matrices may offer additional strategies to improve anthocyanin stability during processing and storage [125,126]. However, systematic comparative studies specifically evaluating fresh, frozen, thermally processed, dried, and fermented *Gaultheria* fruits are still lacking. Future research should therefore monitor individual anthocyanins, total phenolics, salicylate derivatives, and volatile constituents throughout processing and shelf life to identify conditions that best balance phytochemical retention, sensory quality, safety, and technological feasibility.

### 3.10. Commercial Applications and Regulatory Considerations

Despite their long-standing ethnobotanical use and favourable phytochemical profile, the commercial exploitation of *Gaultheria* fruits remains limited. Current applications are mainly local or small-scale and include fresh or dried berries, jams, jellies, syrups, fermented beverages, liqueurs, confectionery flavourings, and other traditional products. *G. shallon* appears to have the strongest culinary potential, whereas *G. procumbens* fruits are valued mainly for their characteristic wintergreen flavour. Experimental food applications have also been reported, including a yogurt beverage fortified with *G. shallon* berries, which showed increased phenolic and anthocyanin contents and antioxidant capacity, although anthocyanins decreased during refrigerated storage [46]. These observations align with broader trends in berry-based functional foods, where technological stability and preservation of bioactive compounds remain key challenges [2].

The high anthocyanin content of some *Gaultheria* species also supports their potential use as natural colourant-rich ingredients, although anthocyanins are sensitive to environmental and processing factors, including pH, temperature, oxygen, and storage conditions [126,127]. Cosmetic applications are also possible: an ethanolic extract of *G. glomerata* fruits has been incorporated experimentally into lipstick, showing acceptable physicochemical stability, microbiological quality, and sensory properties [47].

Commercialisation, however, cannot be based solely on traditional use or in vitro activity. In the European Union, regulatory requirements depend on the specific species, plant part, preparation, intended use, and claims. Ingredients without a documented history of significant food consumption in the EU before 15 May 1997 may require assessment under Regulation (EU) 2015/2283 on novel foods [128]. Importantly, the regulatory status of a whole fruit cannot automatically be extrapolated to concentrated extracts or purified fractions.

Health and therapeutic claims require particular caution. Health claims made on foods must comply with (EC) No 1924/2006 [129]. Therefore, antioxidant, anti-inflammatory, antidiabetic, or neuroprotective effects demonstrated mainly in cell-free or cellular models cannot currently justify therapeutic claims for *Gaultheria*-based foods or supplements. This is particularly important because evidence on in vivo efficacy, bioavailability, safety, and human clinical effectiveness remains limited.

Regulatory pathways also differ substantially for cosmetic and medicinal applications. In the European Union, cosmetic products containing *Gaultheria*-derived ingredients must comply with Regulation (EC) No 1223/2009 on cosmetic products, including requirements concerning product safety, ingredient assessment, manufacturing, labelling, and market responsibility [130]. If *Gaultheria* fruits, extracts, or their constituents were intended to be marketed as medicinal products, they would fall within the framework of Directive 2001/83/EC on medicinal products for human use; plant-derived preparations may additionally be subject to the specific provisions applicable to herbal and traditional herbal medicinal products [131]. Consequently, the regulatory status of a *Gaultheria*-based preparation depends not only on its botanical source and composition but also on its intended use, formulation, claims, and product category.

Finally, commercial development will require rigorous botanical authentication and compositional standardisation. Current approaches to botanical safety emphasise careful identification of plant material, sourcing, chemical characterisation, and extract reproducibility before biological and toxicological evaluation [132]. For *Gaultheria*, particular attention should be given to polyphenols, including anthocyanins, methyl salicylate, and salicylate glycosides because their concentrations may influence both functionality and safety. Overall, *Gaultheria* berries should currently be regarded as an emerging rather than established functional-food resource, with the most realistic near-term applications in conventional berry products, fermented foods, powders, natural colourants, and selected cosmetic formulations. Nutraceutical and medicinal applications require further safety, bioavailability, dose–response, and human clinical studies.

## 4. Research Gaps and Future Perspectives

Future research on *Gaultheria* fruits should progress from descriptive phytochemical and in vitro studies toward a stepwise translational framework integrating food science, pharmacology, toxicology, and regulatory assessment. Based on the current evidence, eight research priorities can be identified.

### 4.1. Phytochemical Standardisation and Metabolomic Profiling

A priority is the comprehensive chemical characterisation of fruits from different *Gaultheria* species, geographical origins, maturity stages, and harvest seasons. Untargeted and targeted LC–MS/MS, GC–MS, and metabolomic approaches should be combined with quantitative determination of anthocyanins, flavonols, proanthocyanidins, hydroxycinnamic acids, iridoids, salicylate glycosides, and volatile constituents. Such studies are necessary to identify species-specific chemical markers and establish reproducible compositional standards for future food, nutraceutical, or medicinal preparations.

### 4.2. Bioaccessibility, Metabolism, and Pharmacokinetics

The biological relevance of compounds detected in berries should be verified using simulated gastrointestinal digestion, intestinal transport models, and metabolite profiling. Particular attention should be given to anthocyanins, iridoids, salicylate glycosides, and other compounds whose in vivo exposure may differ substantially from their concentrations in raw fruits. Subsequent pharmacokinetic studies should determine absorption, biotransformation, systemic exposure, and elimination of the major bioactive constituents and their metabolites.

### 4.3. Mechanistic Cellular Studies

Future in vitro experiments should move beyond screening assays and single-concentration designs toward concentration–response studies using physiologically relevant cellular models. Mechanistic investigations should evaluate specific molecular targets and signalling pathways related to inflammation, oxidative stress, glucose metabolism, neuronal function, and intestinal homeostasis. Standardised positive controls, adequate biological replication, and validated endpoints should be incorporated to improve comparability and reproducibility.

### 4.4. Animal Validation of Biological Effects

Only those effects supported by reproducible chemical and cellular evidence should progress to well-designed animal studies. Priority could be given to metabolic, inflammatory, gastrointestinal, and oxidative-stress-related models, provided that exposure levels reflect realistic dietary or pharmacological doses. Such studies should include pharmacokinetic measurements, dose–response relationships, and appropriate toxicological endpoints rather than relying exclusively on efficacy outcomes.

### 4.5. Human Intervention and Clinical Studies

Controlled human studies represent the essential next step before health-promoting or therapeutic claims can be considered. Initial trials should focus on well-characterised berry preparations and measurable nutritional or physiological endpoints, such as postprandial glucose responses, inflammatory biomarkers, vascular function, or antioxidant status. Larger clinical studies should follow only after safety, bioavailability, and biologically relevant exposure have been demonstrated.

### 4.6. Safety Assessment and Dose Determination

The establishment of safe intake levels is particularly important because some *Gaultheria* species contain substantial amounts of salicylate glycosides and methyl salicylate-related compounds. Future studies should distinguish between traditional consumption of whole berries and exposure to concentrated extracts, powders, supplements, or isolated fractions. Acute and repeated-dose toxicity, potential drug interactions, hypersensitivity risk, and species-specific variation should be evaluated before recommending regular or concentrated intake.

### 4.7. Processing, Formulation, and Food-Product Development

Technological studies should determine how freezing, drying, heating, fermentation, encapsulation, and storage affect anthocyanins, total phenolics, iridoids, salicylate derivatives, and volatile constituents. Product development should prioritise realistic food formats, including juices, fermented beverages, berry powders, preserves, and fortified foods, while balancing phytochemical retention, sensory quality, microbiological safety, and consumer acceptability. Encapsulation and other stabilisation strategies may be useful where they improve stability or bioaccessibility, but these approaches should be evaluated experimentally rather than assumed to confer functional benefit.

### 4.8. Regulatory and Commercial Translation

Translation into commercial products requires simultaneous evaluation of botanical identity, compositional standardisation, safety, technological feasibility, and regulatory status. The intended product category should be defined early, since requirements differ substantially among conventional foods, food supplements, cosmetics, and medicinal products. Regulatory assessment should also consider whether the specific species, plant part, and preparation have an established history of food use. Development strategies at the food–medicine interface should therefore proceed cautiously and should not rely on pharmacological claims until supported by appropriate preclinical and clinical evidence.

Overall, the future development of *Gaultheria* berries should follow a sequential evidence-based pathway: chemical standardisation → bioaccessibility and pharmacokinetics → mechanistic validation → animal studies → human intervention → safety and dose establishment → technological optimisation → regulatory and commercial translation. Currently, the strongest justification supports further investigation of these berries as potential food ingredients and sources of bioactive compounds rather than as established therapeutic agents. Their eventual use in functional foods, nutraceuticals, or phytopharmaceutical preparations will depend on generating robust, standardised, and clinically relevant evidence.

## 5. Conclusions

*Gaultheria* fruits represent a chemically diverse and comparatively underexplored group of edible berries. Available phytochemical studies show polyphenols, including anthocyanins, flavonoids, proanthocyanidins, and hydroxycinnamic acid derivatives, along with distinctive salicylate glycosides, iridoids, and volatile constituents. Experimental studies have reported antioxidant, anti-inflammatory, antimicrobial, antidiabetic, neuroprotective, cytoprotective, and other biological activities; however, the evidence is predominantly derived from cell-free and cellular models and should therefore be considered preliminary with respect to human health effects.

Considerable interspecific variation, differences in extraction and analytical methodologies, and the limited availability of directly comparable nutritional data currently restrict conclusions regarding the relative nutritional or biological value of individual species. Moreover, bioavailability, pharmacokinetics, effective dietary exposure, long-term safety, and clinical efficacy remain insufficiently characterised, and no specific health-promoting or therapeutic effects of *Gaultheria* berry consumption are currently established in humans.

Consequently, *Gaultheria* berries should presently be regarded as promising candidates for further phytochemical, nutritional, and food-technological investigation rather than as established functional or therapeutic products. Future research should prioritise compositional standardisation, bioaccessibility and pharmacokinetic studies, mechanistically robust experimental models, safety and dose assessment, and controlled human interventions. Only after such evidence becomes available will it be possible to determine whether their promising phytochemical and in vitro properties can be translated into substantiated nutritional, functional-food, or health-related applications.

## Figures and Tables

**Table 1:** Botanical characteristics of selected *Gaultheria* fruits.

Plant	Main Botanical Characteristics	Area of Occurrence/Distribution	Fruit Colour	Fruit Size (Diameter)	References
*Gaultheria antarctica* Hook.f.	Evergreen shrub; leaves small, simple, ovate to lanceolate, coriaceous and glabrous, with serrate margins and a shortly mucronate apex; flowers solitary or in small cymes, campanulate and 5-merous.	native to southern Chile and southern Argentina and found also on the Falkland Islands	red to purple	up to ~10 mm	[12,23,24,25]
*Gaultheria erecta* Vent.	Erect to spreading evergreen shrub, occasionally epiphytic; leaves relatively large, coriaceous, ovate to elliptic, finely serrate and often glandular-punctate; flowers numerous in axillary racemes, with an urceolate to cylindric-urceolate corolla.	from Mexico through Central America (Guatemala, Honduras, Nicaragua, Costa Rica, Panama) into northern South America (Colombia, Venezuela) and down through the Andes into Ecuador, Peru and Bolivia, with extensions into Brazil and northern Argentina	blue-black	7–12(15) mm	[12,23,24,26,27]
*Gaultheria fragrantissima* Wall.	Robust evergreen shrub or small tree; leaves alternate, lanceolate to ovate, coriaceous, gland-dotted, with crenate-serrate margins; fragrant, bell-shaped flowers arranged in dense axillary racemes.	native to southern and southeastern Asia, from the Indian subcontinent (including India, Nepal and Sri Lanka) eastward through Tibet and south-central China and into Myanmar, Vietnam, Peninsular Malaysia, Sumatra, Java and Bali	blue-purple	4–6 mm	[12,23,24,26,28]
*Gaultheria glomerata* (Cav.) Sleumer	Evergreen shrub with simple, coriaceous leaves; flowers small and urceolate to campanulate, borne in compact axillary clusters; fruiting calyx becomes fleshy and surrounds the true capsular fruit.	western South America (Colombia, Ecuador, Venezuela, Peru and Bolivia)	blue-black	6–9 mm	[12,23,24,26,27]
*Gaultheria hispidula* (L.) Muhl. ex Bigelow	Low, creeping, stoloniferous, mat-forming evergreen subshrub; stems densely strigose; leaves small, elliptic to oval, ciliate and often hairy beneath; flowers solitary and axillary, with a campanulate corolla.	native to northern and eastern United States and Canada, historically ranging from northern Canada southward to North Carolina	white	~4–6 mm	[12,23,24,26]
*Gaultheria mucronata* (L.f.) Hook. & Arn.	Compact, strongly suckering evergreen shrub, usually dioecious; leaves small, alternate, coriaceous, glossy, ovate to lanceolate, with a conspicuous mucronate apex; flowers solitary in the upper leaf axils, urceolate to bell-shaped.	southern Andes of South America, especially Chile and Argentina	white, pink, red, or lilac	~12 mm	[12,20,23,24,25,26,27,29,30,31]
*Gaultheria myrsinoides* Kunth	Evergreen shrub with densely arranged, shortly petiolate, oblong-ovate coriaceous leaves with bristly serrate margins; flowers solitary and axillary, nodding, with an ovoid-urceolate corolla.	Mexico and Central America (Guatemala, Honduras, Nicaragua, Costa Rica, Panama) and extending into parts of South America (Colombia, Venezuela, Ecuador, Peru, Bolivia, Argentina)	red, pink, white, dark purple, or purplish	up to ~15 mm	[12,23,24,26]
*Gaultheria phillyreifolia* (Pers.) Sleumer	Dense evergreen shrub, commonly up to about 1–2.5 m tall; leaves small, coriaceous, ovate to obovate-lanceolate, serrate and sharply pointed; flowers solitary or few-flowered, axillary and urceolate.	Chile and Argentina (southern South America)	pink, white, red, brown, or dark purple	5–10 mm	[12,23,24,25,26]
*Gaultheria poeppigii* DC.	Evergreen shrub; leaves simple, coriaceous and relatively small, with toothed margins; flowers predominantly solitary or arranged in small axillary cymes, with a typical urceolate corolla; fruit is a capsule associated with a fleshy calyx.	South America (Andean regions of Chile and/or Peru)	pink, white, red, brown, or dark purple	5–10 mm	[12,23,24,25,26]
*Gaultheria procumbens* L.	Low, creeping, rhizomatous evergreen subshrub forming ground-covering mats; leaves simple, alternate, leathery and aromatic; flowers small, solitary or few together in the leaf axils, pendulous and urceolate.	eastern Canada and the eastern United States, from Newfoundland and Québec west to Manitoba and south to the Appalachian and Mid-Atlantic states	red (outer), white (inner)	8–15 mm	[12,23,24,26]
*Gaultheria pumila* (L.f.) D.J.Middleton	Dwarf, creeping to erect evergreen shrub spreading by underground stems shortly; leaves very small, elliptic to oblong-lanceolate, coriaceous and mostly entire or weakly crenate; flowers solitary and axillary.	southern South America (Chile and Argentina)	white, pink, or red	usually 6–7 mm (up to ~12 mm)	[12,23,24,25,26,32,33]
*Gaultheria shallon* Pursh	Evergreen, suckering shrub forming dense thickets; leaves large, alternate, ovate to elliptic, thick, leathery and finely serrate; flowers pendulous, urceolate, borne in terminal or axillary racemes.	Pacific Northwest of North America, from Alaska south along the west coast through British Columbia, Washington, Oregon and northern California	purple to black	6–10 mm	[12,23,24,26,34,35]
*Gaultheria tenuifolia* (Phil.) Sleumer	Erect evergreen shrub with angular, glabrous branchlets; leaves narrow, linear-elliptic to oblong-elliptic, coriaceous, glossy, mucronate and finely toothed; dioecious, with solitary, pendulous axillary flowers.	southern South America (Chile, Argentina) and possibly neighbouring Andean regions	reddish-brown	4–5 mm	[12,23,24,25,26]
*Gaultheria trichophylla* Royle	Dwarf, prostrate, densely branched evergreen shrub spreading by underground shoots; leaves small, elliptic to elliptic-lanceolate, leathery and characteristically long-ciliate at the margins; flowers solitary and axillary, campanulate.	southwestern to central Asia, including the Himalayas (e.g., India, Nepal, Pakistan)	sky blue (outer), white-pinkish (inner)	8–11 mm	[12,23,24,26]

**Table 2:** Comparative overview of the major bioactive compound groups quantified in *Gaultheria* fruits.

*Gaultheria* Species	TPC	Total Anthocyanins	Total Flavonoids	Total Proanthocyanidins	Total Salicylates	Total Iridoids	Main Phytochemical Distinction	References
*G. antarctica* Hook.f.	n.r.	0.19 μmol/g fw	Flavonols: 0.05 μmol/g fw	n.r.	n.r.	Coumaroyl-iridoid: 0.26 μmol/g fw	Moderate anthocyanin and iridoid occurrence; data expressed in molar units	[64,65,66]
*G. erecta* Vent.	306.4–881.3 mg GAE/g dw	n.r.	Flavonoids: 90.7 mg QE/g dw	n.r.	n.r.	n.r.	Among the highest reported TPC values	[67,68]
*G. fragrantissima* Wall.	80.4 mg GAE/100 g fw	2.47 mg CGE/100 g fw	Flavonoids: 94.3 mg QE/100 g fw	n.r.	n.r.	n.r.	Moderate phenolic/flavonoid content	[69,70]
*G. glomerata* (Cav.) Sleumer	344.4–1804.1 mg GAE/100 g fw †	19.2–112.9 mg CGE/100 g fw †	n.r.	n.r.	n.r.	n.r.	Very high TPC; anthocyanin-rich	[61,71,72,73,74,75]
*G. mucronata* (L.f.) Hook. & Arn.	n.r.	1.23 μmol/g fw	Flavonols: 0.32 μmol/g fw	n.r.	n.r.	Coumaroyl-iridoid: 0.64 μmol/g fw	Relatively high anthocyanin and coumaroyl-iridoid levels; additionally rich in cuticular triterpenoids	[64,65,66,76,77]
*G. myrsinoides* Kunth	6.08 μg GAE/mg dw extract	1655.2 mg CGE/100 g dw	detected ¶	n.r.	n.r.	n.r.	High anthocyanin content on a dry-weight basis; limited quantitative phytochemical data available	[74,78,79]
*G. phillyreifolia* (Pers.) Sleumer	147.5–290.3 mg GAE/g dw	32.3–293.8 mg/100 g fw	Flavonols: 46.4–163.6 mg/100 g fw	18.2–69.3 mg CAE/g dw	n.r.	359.6–1334.1 mg/100 g fw	Outstanding source of iridoids; high flavonols and anthocyanins	[65,80,81,82,83]
*G. poeppigii* DC.	160.9–284.4 mg GAE/g dw	17.7–60.3 mg/100 g fw	Flavonols: 14.6–71.0 mg/100 g fw	25.3–68.1 mg CAE/g dw	n.r.	70.8–484.2 mg/100 g fw	Iridoid-rich, but lower than *G. phillyreifolia*	[65,80,81,82,83,84]
*G. procumbens* L.	17.26–79.71 mg GAE/g dw	n.r.	Flavonols: 0.065–1.17 mg/g dw	7.41–9.59 mg/g dw	31.09–121.67 mg/g dw	n.r.	Unique and richest documented source of salicylate glycosides, especially gaultherin	[17,85,86,87,88]
*G. pumila* (L.f.) D.J.Middleton	189.2 mg GAE/g dw	626.2–5942 mg CGE/100 g fw	Flavonoids: 51.8 mg QE/g dw	n.r.	n.r.	detected ¶	Exceptionally anthocyanin-rich in some fruit colour forms	[89,90]
*G. shallon* Pursh	658–975 mg GAE/100 g fw	134–256 mg CGE/100 g fw	detected ¶	52.8–280.7 mg/g dw	n.r.	n.r.	High TPC, anthocyanins and particularly proanthocyanidins	[91,92,93,94,95]
*G. tenuifolia* (Phil.) Sleumer	n.r.	quantified #	Flavonoids: 80.33–93.72 mg/100 g fw	34.87–131.66 mg/100 g fw	n.r.	21.65–22.22 mg/100 g fw	Rich in flavonoids and proanthocyanidins; lower iridoids	[96]
*G. trichophylla* Royle	3.28–3.71 mg GAE/100 g fw	n.r.	Flavonols: 0.86–1.03 mg CAE/100 g fw	n.r.	n.r.	n.r.	Relatively low reported phenolic/flavonoid contents	[62,63,70,97,98]

CAE, catechin equivalents; CGE, cyanidin-3-glucoside equivalents; dw, dry weight; fw, fresh weight; GAE, gallic acid equivalents; n.r., not reported quantitatively; QE, quercetin equivalents; TPC, total phenolic content. † Values depend strongly on sample processing and analytical protocol; fresh, frozen and dried *G. glomerata* fruits showed substantial differences in TPC and anthocyanin content. ¶ Compounds were identified, but directly comparable total values are unavailable. # Detailed quantitative data are available in Supplementary Table S1.

**Table 3:** The biological activity of *Gaultheria* fruits.

Plant	Method/Activity Results	Positive Control	References
antioxidant activity			
*G. antarctica* Hook.f.	Methanol-formic acid (97:3, *v*/*v*) Activity expressed in μmol TX/g fw of the fruits: TEAC (ABTS assay; Trolox equivalents): 20.3 μmol/g fw	Trolox—standard curve TEAC: *Fuchsia magellanica* 10.4 μmol TX/g fw	[64]
	Methanol-water (97:3, *v*/*v*) after SPE Activity expressed in μmol TX/g fw of the fruits: CUPRAC (Trolox equivalents): 21.92 μmol/g fw	Trolox—standard curve CUPRAC: *Fuchsia magellanica* 8.61 μmol TX/g fw	[66]
*G. erecta* Vent.	Methanolic extract (different maturation states: immature, intermediate, mature fruits) Activity expressed in μmol/g dw of the extract: TEAC (ABTS assay; Trolox equivalents): 1049.4–2029.9 μmol TX/g dw; ORAC (Trolox equivalents): 385.6–1668.4 μmol TX/g dw	Trolox—standard curve Methanol extract TEAC: *Camelia sinensis* 2014.7 μmol TX/g dw; ORAC: *Camelia sinensis* 1417.7 μmol TX/g dw	[67]
	Ethanolic extract Activity expressed as % DPPH inhibition or mg/L of the extract: DPPH: 16.38% (extract concentration 100 mg/L), IC_50_ = 203 mg/L	DPPH; AA: 86.25% (100 mg/L), IC_50_ = 52 mg/L; GA: 94.11% (100 mg/L), IC_50_ = 43 mg/L	[68]
*G. fragrantissima* Wall.	Methanol-water (70:30, *v*/*v*) Activity expressed in μg/mL of the extract: DPPH: IC_50_ = 240.2 μg/mL	DPPH: *Flueggea leucopyrus* IC_50_ = 76.8 μg/mL	[69,70]
*G. glomerata* (Cav.) Sleumer	Ethanol-1% citric acid Activity expressed in mg/100 mL of the extract: ABTS: 5730 mg AAE/100 mL; DPPH: 4872 mg AAE/100 mL	Ascorbic acid—standard curve	[71]
	Functional beverage combining fruit juice of *G. glomerata* with stem juice of *Oxalis tuberosa* Activity expressed in μmol/100 g of the fresh juice: TEAC (ABTS assay; Trolox equivalents): 76.92–89.56 μmol TX/100 g	Trolox—standard curve	[61]
	Methanolic extract acidified with 0.01% HCl Activity expressed in μmol/g dw of the extract: DPPH (Trolox equivalents): 38.39 μmol TX/g dw (fresh), 35.10 μmol TX/g dw (frozen), 36.81 μmol TX/g dw (dried)	Trolox—standard curve	[73]
*G. hispidula* (L.) Muhl. ex Bigelow	Ethanol-water (80:20, *v*/*v*) Activity expressed in ppm of the extract: DPPH: IC_50_ = 25.44 ppm	DPPH; AA: IC_50_ = 3.84 ppm; QU: IC_50_ = 4.42 ppm; CA: IC_50_ = 5.56 ppm; ECA: IC_50_ = 5.94 ppm	[107]
*G. mucronata* (L.f.) Hook. & Arn.	Methanol-formic acid (97:3, *v*/*v*) Activity expressed in μmol/g fw of the fruits: TEAC (ABTS assay; Trolox equivalents): 28.9 μmol TX/g fw	Trolox—standard curve TEAC: *Fuchsia magellanica* 10.4 μmol TX/g fw	[64]
	Methanol-water (97:3, *v*/*v*) after SPE Activity expressed in μmol/g fw of the fruits: CUPRAC (Trolox equivalents): 41.43 μmol TX/g fw	Trolox—standard curve CUPRAC: *Fuchsia magellanica* 8.61 μmol TX/g fw	[66]
*G. myrsinoides* Kunth	Ethanol-water (70:30, *v*/*v*) acidified with citric acid (pH = 3.3) Activity expressed as % DPPH inhibition or μg/mL of the extract: DPPH: 46.5% (extract concentration 100 μg/mL), IC_50_ = 220.3 μg/mL	DPPH; TX: IC_50_ = 36.6 μg/mL	[74]
	Extract unknown Activity expressed in μg/mL of the extract: DPPH: IC_50_ = 85.09 μg/mL	DPPH; AA: IC_50_ = 40.15 μg/mL	[78]
	Ethanolic extract Activity expressed in mmol/g or mg/g dw of the extract: DPPH (Trolox equivalents): IC_50_ = 4.10 mg/g dw, 0.706 mmol TX/g dw; DPPH (Trolox equivalents): 17.93 mmol TX/g dw	Trolox—standard curve	[79]
*G. phillyreifolia* (Pers.) Sleumer	Methanol-formic acid (99:1, *v*/*v*) (different colours of the fruits, and time and place of collection) Activity expressed in μg/mL or μmol/g dw of the extract: DPPH: anthocyanin fraction (3.6–79.0 μg/mL), copigment fraction (9.7–26.5 μg/mL); FRAP: anthocyanin fraction (349.4–6019.6 μmol TX/g dw), copigment fraction (1579.5–2118.9 μmol TX/g dw); TEAC (ABTS assay): anthocyanin fraction (487.1–4435.3 μmol TX/g dw), copigment fraction (1186.9–2014.4 μmol TX/g dw); CUPRAC: anthocyanin fraction (524.4–3226.6 μmol TX/g dw), copigment fraction (1072.5–1552.9 μmol TX/g dw); ORAC: anthocyanin fraction (597.9–2742.4 μmol TX/g dw), copigment fraction (1050.6–4403.4 μmol TX/g dw)	DPPH; QU: 7.8 μg/mL; FRAP; QU: 1077.2 μmol TX/g dw; TEAC (ABTS assay); QU: 8157.9 μmol TX/g dw; CUPRAC; QU: 27,526.1 μmol TX/g dw; ORAC; QU: 22,561.6 μmol TX/g dw	[80,81]
	Methanol-formic acid (99:1, *v*/*v*) RT-qPCR, activity expressed as fold-change relative to untreated cells (set to 1.0) post-AAPH treatment; Caco-2 human intestinal epithelial cells Bioaccessible (in vitro digested) whole fruit extracts (concentration 200 μg/mL): Antioxidant genes involved in the Keap1-Mrf2/ARE pathway: NFE2L2 (Nrf2; ~5760-fold), KEAP1 (~1.8-fold), HMOX1 (~25–30-fold), SOD1 (~1.5-fold), SOD2 (~1.8-fold), CAT (~1.6-fold), GPX1 (~35-fold), GPX-2 (~3.5–4.0-fold), GPX-3 (~2.5-fold), GPX-4 (~3.5–4.0-fold), GSR (~1.8-fold). RT-qPCR, activity expressed as fold-change relative to untreated cells (set to 1.0) post-AAPH treatment; Caco-2 human intestinal epithelial cells Bioaccessible (in vitro digested) whole fruit extracts (concentration 200 μg/mL): Antioxidant enzymes and proteins involved in the Keap1-Mrf2/ARE pathway: Nrf2 (~2.5-fold), KEAP1 (~0.7-fold), HO-1 (~1.8-fold), SOD1 (~1.5-fold), CAT (~1.6-fold), GPX1 (~1.7-fold)	RT-qPCR, activity expressed as fold-change relative to untreated cells (set to 1.0) post-AAPH treatment Caco-2 human intestinal epithelial cells Cyanidin-3-*O*-β-arabinoside (100 μM) Antioxidant genes involved in the Keap1-Mrf2/ARE pathway: NFE2L2 (Nrf2; ~1500–1900-fold), KEAP1 (~2.0-fold), HMOX1 (~40-fold), SOD1 (~2.0-fold), SOD2 (~2.2-fold), CAT (~2.8–3.0-fold), GPX1 (~105-fold), GPX-2 (~4.0-fold), GPX-3 (~5.0-fold), GPX-4 (~8–10-fold), GSR (~2.5-fold). Cyanidin-3-*O*-β-arabinoside (100 μM) Antioxidant enzymes and proteins involved in the Keap1-Mrf2/ARE pathway: Nrf2 (~3.5-fold), KEAP1 (~0.6-fold), HO-1 (~2.5-fold), SOD1 (~2.0-fold), CAT (~2.8-fold), GPX1 (~3.0-fold)	[83]
*G. poeppigii* DC.	Methanol-formic acid (99:1, *v*/*v*) (different colours of the fruits, and time and place of collection) Activity expressed in μg/mL or μmol/g dw of the extract: DPPH: anthocyanin fraction (11.2–71.6 μg/mL), copigment fraction (16.4–32.7 μg/mL); FRAP: anthocyanin fraction (113.3–1445.5 μmol TX/g dw), copigment fraction (1455.9–2049.1 μmol TX/g dw); TEAC (ABTS assay): anthocyanin fraction (305.8–1080.4 μmol TX/g dw), copigment fraction (1329.6–1952.1 μmol TX/g dw); CUPRAC: anthocyanin fraction (355.2–907.8 μmol TX/g dw), copigment fraction (862.7–1638.6 μmol TX/g dw); ORAC: anthocyanin fraction (79.2–786.4 μmol TX/g dw), copigment fraction (1220.5–4967.8 μmol TX/g dw)	DPPH; QU: 7.8 μg/mL; FRAP; QU: 1077.2 μmol TX/g dw; TEAC (ABTS assay); QU: 8157.9 μmol TX/g dw; CUPRAC; QU: 27,526.1 μmol TX/g dw; ORAC; QU: 22,561.6 μmol TX/g dw	[80,81]
	Methanol-formic acid (99:1, *v*/*v*) RT-qPCR, activity expressed as fold-change relative to untreated cells (set to 1.0) post-AAPH treatment; Caco-2 human intestinal epithelial cells Bioaccessible (in vitro digested) whole fruit extracts (concentration 200 μg/mL): Antioxidant genes involved in the Keap1-Mrf2/ARE pathway: NFE2L2 (Nrf2; ~5300-fold), KEAP1 (~1.7-fold), HMOX1 (~20–25-fold), SOD1 (~1.4-fold), SOD2 (~2.0-fold), CAT (~1.8-fold), GPX1 (~36-fold), GPX-2 (~3.0–3.5-fold), GPX-3 (~2.2-fold), GPX-4 (~3.0–3.5-fold), GSR (~1.6-fold). RT-qPCR, activity expressed as fold-change relative to untreated cells (set to 1.0) post-AAPH treatment; Caco-2 human intestinal epithelial cells Bioaccessible (in vitro digested) whole fruit extracts (concentration 200 μg/mL): Antioxidant enzymes and proteins involved in the Keap1-Mrf2/ARE pathway: Nrf2 (~2.2-fold), KEAP1 (~0.8-fold), HO-1 (~1.6-fold), SOD1 (~1.4-fold), CAT (~1.7-fold), GPX1 (~1.6-fold)	RT-qPCR, activity expressed as fold-change relative to untreated cells (set to 1.0) post-AAPH treatment Caco-2 human intestinal epithelial cells Cyanidin-3-*O*-β-arabinoside (100 μM) Antioxidant genes involved in the Keap1-Mrf2/ARE pathway: NFE2L2 (Nrf2; ~1500–1900-fold), KEAP1 (~2.0-fold), HMOX1 (~40-fold), SOD1 (~2.0-fold), SOD2 (~2.2-fold), CAT (~2.8–3.0-fold), GPX1 (~105-fold), GPX-2 (~4.0-fold), GPX-3 (~5.0-fold), GPX-4 (~8–10-fold), GSR (~2.5-fold). Cyanidin-3-*O*-β-arabinoside (100 μM) Antioxidant enzymes and proteins involved in the Keap1-Mrf2/ARE pathway: Nrf2 (~3.5-fold), KEAP1 (~0.6-fold), HO-1 (~2.5-fold), SOD1 (~2.0-fold), CAT (~2.8-fold), GPX1 (~3.0-fold)	[83]
	Methanol-water (75:25, *v*/*v*) (different colours of the fruits, and time and place of collection) Activity expressed in μg/mL or μmol TX/g fw of the fruits TEAC (ABTS assay; Trolox equivalents): 5.53–18.72 μmol TX/g fw; CUPRAC (Trolox equivalents): 20.82–113.41 μmol TX/g fw; DPPH (Trolox equivalents): 3.71–10.22 μmol TX/g fw	Trolox—standard curve	[84]
*G. procumbens* L.	Activity results expressed in μg/mL or mmol Fe^2+^/g of the extract: DPPH; methanol-water (75:25, *v*/*v*): EC_50_ = 40.40 μg/mL, *n*-butanol extract: 237.00 μg/mL, acetone extract: 44.59 μg/mL, water extract: 75.46 μg/mL; FRAP; methanol-water (75:25, *v*/*v*): 1.65 mmol Fe^2+^/g, *n*-butanol extract: 0.93 mmol Fe^2+^/g, acetone extract: 1.75 mmol Fe^2+^/g, water extract: 0.99 mmol Fe^2+^/g; TBARS; methanol-water (75:25, *v*/*v*): IC_50_ = 56.67 μg/mL, *n*-butanol extract: 251.68 μg/mL, acetone extract: 37.41 μg/mL, water extract: 71.84 μg/mL; O_2_^•–^; methanol-water (75:25, *v*/*v*): SC_50_ = 333.41 μg/mL, *n*-butanol extract: 877.63 μg/mL, acetone extract: 175.33 μg/mL, water extract: 322.94 μg/mL; OH^•^; methanol-water (75:25, *v*/*v*): SC_50_ = 824.04 μg/mL, *n*-butanol extract: 676.29 μg/mL, acetone extract: 863.95 μg/mL, water extract: 1150.67 μg/mL; H_2_O_2_; methanol-water (75:25, *v*/*v*): SC_50_ = 332.15 μg/mL, *n*-butanol extract: 497.95 μg/mL, acetone extract: 166.36 μg/mL, water extract: 422.95 μg/mL. *f*MLP-stimulated human neutrophils model: ROS; acetone extract: level of ROS 83.8% (extract concentration 25 μg/mL), 72.1% (50 μg/mL), 55.2% (100 μg/mL), 35.5% (150 μg/mL)	DPPH; QU: EC_50_ = 1.65 μg/mL, TX: 4.31 μg/mL; FRAP; QU: 47.09 mmol Fe^2+^/g, TX: 11.89 mmol Fe^2+^/g; TBARS; QU: IC_50_ = 1.78 μg/mL, TX: 4.68 μg/mL; O_2_^•–^; QU: SC_50_ = 7.58 μg/mL, TX: 135.24 μg/mL; OH^•^; QU: SC_50_ = 42.48 μg/mL, TX: 165.45 μg/mL; H_2_O_2_; QU: SC_50_ = 7.52 μg/mL, TX: 15.87 μg/mL. *f*MLP-stimulated human neutrophils model: ROS; QU: 17.8% (25 μM)	[17,85]
	Aqueous citric acid extract (pH = 2.0) (different harvest times: October 1–15, 2006; October 2–30, 2006; March 3–15, 2007) Activity expressed in % of radical scavenging: DPPH: 85.88–89.28% (extract concentration 1 mg/mL)	DPPH: AA: ~95% (0.1 mg/mL)	[108]
	Activity expressed in % of radical scavenging: DPPH; 0.1 M aqueous citric acid extract: 89.2% (extract concentration 0.8 mg/mL), liquid methanol-water extract (80:20, *v*/*v*): 92.3% (0.8 mg/mL); ABTS; 0.1 M aqueous citric acid extract: 47.7% (0.8 mg/mL), liquid methanol-water extract (80:20, *v*/*v*): 46.2% (0.8 mg/mL)	–	[109]
	UVA-irradiated human Hs68 dermal fibroblasts ROS; acetone extract: level of ROS 95.5% (extract concentration 5 μg/mL), 90.9% (10 μg/mL), 78.3% (25 μg/mL); Superoxide dismutase; acetone extract: % of enzyme activity 102.6% (extract concentration 5 μg/mL), 110.3% (10 μg/mL), 120.9% (25 μg/mL); Glutathione *S*-transferase; acetone extract: % of enzyme activity 107.6% (extract concentration 5 μg/mL), 116.9% (10 μg/mL), 127.8% (25 μg/mL)	ROS; QU: 49.3% (25 μg/mL); AA: 58.6% (25 μg/mL); Superoxide dismutase; QU: 141.2% (25 μg/mL); AA: 148.2% (25 μg/mL); Glutathione *S*-transferase; QU: 113.9% (25 μg/mL); AA: 119.9% (25 μg/mL)	[86]
*G. pumila* (L.f.) D.J.Middleton	Methanol-formic acid (98:2, *v*/*v*) Activity expressed in μg/mL or μmol TX/g dw of the extract: DPPH: IC_50_ = 92.8 μg/mL; FRAP (Trolox equivalents): 134.1 μmol TX/g dw; ORAC (Trolox equivalents): 4251.6 μmol TX/g dw	DPPH: GA: IC_50_ = 0.55 μg/mL; Trolox—standard curve	[90]
*G. shallon* Pursh	Ethyl acetate fraction of methanolic extract Activity expressed in μg/mL dw of the extract: DPPH: IC_50_ = 14.76 μg/mL	DPPH; AA: IC_50_ = 18.53 μg/mL	[91]
	Acetonitrile-0.2% formic acid (different harvest times: 2012, 2013, 2014) Activity expressed in μmol Fe^2+^/100 g fw of the fruits: FRAP: 63.048–100.815 μmol Fe^2+^/100 g fw	Ferrous ion—standard curve	[92]
	Acetonitrile-0.2% formic acid Activity expressed in μmol Fe^3+^ equivalents: FRAP: 101.225 μmol Fe^3+^ equivalents	Ferrous ion—standard curve	[93]
	Methanolic extract (different stages of fruit development: immature and mature berries) Activity expressed in mmol/100 g dw of the fruits: ABTS (Trolox equivalents): ~36–106 mmol TX/100 g dw	Methanolic extract Mature blueberry fruits ABTS (Trolox equivalents): ~13 mmol TX/100 g dw	[94]
*G. trichophylla* Royle	Methanol-water (80:20, *v*/*v*) Activity expressed in mmol/100 g fw of the fruits: DPPH (Ascorbic acid equivalents): 2.56 mmol AAE/100 g fw; FRAP (Ascorbic acid equivalents): 3.99 mmol AAE/100 g fw; ABTS (Ascorbic acid equivalents): 4.35 mmol AAE/100 g fw	Ascorbic acid—standard curve	[62]
	Methanolic extract Activity results expressed in μg/mL of the extract: DPPH: IC_50_ = 23.81 μg/mL	DPPH; AA: IC_50_ = 136.20 μg/mL	[98]
	Methanolic extract Activity results expressed in μg/mL of the extract: DPPH: IC_50_ = 81.2 μg/mL; FRAP: IC_50_ = 11.2 μg/mL	–	[70]
anti-inflammatory activity			
*G. procumbens* L.	Activity expressed in μg/mL of the extract: Lipoxygenase inhibition; acetone extract: IC_50_ = 644.79 μg/mL; methanol-water (75:25, *v*/*v*): IC_50_ = 743.61 μg/mL; *n*-butanol extract: IC_50_ = 850.42 μg/mL; water extract: IC_50_ = 837.96 μg/mL; Cyclooxygenase-2; acetone extract: IC_50_ = 152.89 μg/mL; methanol-water (75:25, *v*/*v*): IC_50_ = 230.83 μg/mL; *n*-butanol extract: IC_50_ = 224.08 μg/mL; water extract: IC_50_ = 713.36 μg/mL	Lipoxygenase; IND: IC_50_ = 92.60 μg/mL; DEX: 118.14 μg/mL; Cyclooxygenase-2; IND: IC_50_ = 178.40 μg/mL; DEX: 507.63 μg/mL	[17,85]
	LPS/*f*MLP+cytochalasin B-stimulated human neutrophils IL-8; acetone extract: % of inhibition 0% (extract concentration 25 μg/mL), 5.3% (50 μg/mL), 13.8% (100 μg/mL), 36.2% (150 μg/mL); IL-1β; acetone extract: 28.0% (25 μg/mL), 44.7% (50 μg/mL), 63.9% (100 μg/mL), 79.7% (150 μg/mL); TNF-α; acetone extract: 12.5% (25 μg/mL), 34.0% (50 μg/mL), 46.6% (100 μg/mL), 55.8% (150 μg/mL); MMP-9; acetone extract: 0.6% (25 μg/mL), 5.2% (50 μg/mL), 8.5% (100 μg/mL), 17.9% (150 μg/mL); ELA-2; acetone extract: 72.5% (25 μg/mL), 76.4% (50 μg/mL), 77.1% (100 μg/mL), 81.4% (150 μg/mL)	LPS/*f*MLP+cytochalasin B-stimulated human neutrophils: IL-8; DEX: 55.8% (25 μM); IL-1β; DEX: 51.8% (25 μM); TNF-α; DEX: 68.8% (25 μM); MMP-9; DEX: 26.9% (25 μM); ELA-2; QU: 39.3% (25 μM)	[17,85]
	LPS-stimulated human Hs68 dermal fibroblasts IL-8; acetone extract: % of inhibition 6.6% (extract concentration 5 μg/mL), 63.4% (10 μg/mL), 84.8% (25 μg/mL), 88.1% (50 μg/mL); ICAM-1; acetone extract: % of inhibition 28.4% (extract concentration 5 μg/mL), 29.1% (10 μg/mL), 32.1% (25 μg/mL), 30.5% (50 μg/mL); NF-κB; acetone extract: % of inhibition 14.5% (extract concentration 5 μg/mL), 21.8% (10 μg/mL), 29.1% (25 μg/mL), 38.7% (50 μg/mL); phosphorylation of Erk kinase (pErk); acetone extract: suppression of the LPS-induced Erk kinase activation (25–50 μg/mL)	IL-8; DEX: 92.7% (5 μg/mL), 95.9% (10 μg/mL), 97.9% (25 μg/mL), 99.2% (50 μg/mL); ICAM-1; DEX: 91.4% (5 μg/mL), 99.1% (10 μg/mL), 99.1% (25 μg/mL), 99.1% (50 μg/mL); NF-κB; DEX: 91.1% (5 μg/mL), 92.0% (10 μg/mL), 94.3% (25 μg/mL), 97.8% (50 μg/mL); phosphorylation of Erk kinase (pErk); DEX: suppression of the LPS-induced Erk kinase activation (25–50 μg/mL)	[17,86]
*G. trichophylla* Royle	Methanolic extract Inhibition of protein (bovine serum albumin) denaturation: IC_50_ = 120.98 μg/mL	Aspirin; IC_50_ = 65.09 μg/mL	[98]
anti-diabetic activity			
*G. hispidula* (L.) Muhl. ex Bigelow	The Authors do not specify exactly what part of the plant was used in the study, but it was most likely the fruit. Ethanol-water (80:20, *v*/*v*) Inhibition of intestinal glucose uptake (radiolabeled ^3^H-deoxy-D-glucose uptake assay); Caco-2/15 human intestinal cells 10 min co-incubation: 34% glucose uptake (~66% inhibition vs. control) 6 h pre-incubation: 12% uptake (no inhibition) Pre-incubation + co-incubation: not determined	Control (100% uptake)	[110]
*G. phillyreifolia* (Pers.) Sleumer	Methanol-formic acid (99:1, *v*/*v*) Activity expressed in μg/mL for the non-digested and the end of the in vitro intestinal digestion extract: α-glucosidase: non-digested (IC_50_ = 0.7 μg/mL), digested (IC_50_ = 2.8 μg/mL) extracts; α-amylase (% inhibition at 100 μg/mL): non-digested (inactive), digested (inactive) extracts. Glucose uptake (fmol/cell/min) RT-qPCR, activity expressed as fold-change relative to mRNA expression in untreated cells (set to 1.0) Caco-2 cell model (human intestinal epithelial cells) Bioaccessible fraction after in vitro gastrointestinal digestion (extract concentration 200 μg/mL): 2-deoxyglucose uptake: ~6.2 fmol/cell/min SCLC5A1 (SGLT1; relative mRNA expression): ~0.10; SCLC2A2 (GLUT2; relative mRNA expression): ~0.05; SCLC2A5 (GLUT5; relative mRNA expression): ~0.30; SI (sucrase-isomaltase; relative mRNA expression): ~0.03	Acarbose; α-glucosidase: IC_50_ = 137.0 μg/mL; α-amylase (% inhibition at 100 μg/mL): 28.5%. Caco-2 cells: 2-deoxyglucose uptake: QU ~ 7.3 fmol/cell/min SCLC5A1 (SGLT1; relative mRNA expression): QU ~ 0.40; SCLC2A2 (GLUT2; relative mRNA expression): ~0.20; SCLC2A5 (GLUT5; relative mRNA expression): ~0.55; SI (sucrase-isomaltase; relative mRNA expression): ~0.25	[82]
*G. poeppigii* DC.	Methanol-formic acid (99:1, *v*/*v*) Activity expressed in μg/mL of the non-digested and at the end of the in vitro intestinal digestion extract: α-glucosidase: non-digested (pink fruits IC_50_ = 3.1 μg/mL; white fruits IC_50_ = 2.3 μg/mL), digested (pink fruits IC_50_ = 12.2 μg/mL; white fruits IC_50_ = 24.9 μg/mL) extracts; α-amylase (% inhibition at 100 μg/mL): non-digested (pink fruits: inactive; white fruits: inactive), digested (pink fruits: 12.1%; white fruits: inactive) extracts. Glucose uptake (fmol/cell/min) RT-qPCR, activity expressed as fold-change relative to mRNA expression in untreated cells (set to 1.0) Caco-2 cell model (human intestinal epithelial cells) Bioaccessible fraction after in vitro gastrointestinal digestion (extract concentration 200 μg/mL): 2-deoxyglucose uptake: pink fruits (~6.98 fmol/cell/min), white fruits (~6.5 fmol/cell/min) SCLC5A1 (SGLT1; relative mRNA expression): pink fruits (~0.25), white fruits (~0.62); SCLC2A2 (GLUT2; relative mRNA expression): pink fruits (~0.35), white fruits (~0.55); SCLC2A5 (GLUT5; relative mRNA expression): pink fruits (~0.45), white fruits (~0.72); SI (sucrase-isomaltase; relative mRNA expression): pink fruits (~0.18), white fruits (~0.45)	Acarbose; α-glucosidase: IC_50_ = 137.0 μg/mL; α-amylase (% inhibition at 100 μg/mL): 28.5%. Caco-2 cells: 2-deoxyglucose uptake: QU ~ 7.3 fmol/cell/min SCLC5A1 (SGLT1; relative mRNA expression): QU ~ 0.40; SCLC2A2 (GLUT2; relative mRNA expression): ~0.20; SCLC2A5 (GLUT5; relative mRNA expression): ~0.55; SI (sucrase-isomaltase; relative mRNA expression): ~0.25	[82]
cytocompatibility and cytoprotective activity			
*G. phillyreifolia* (Pers.) Sleumer	Methanol-formic acid (99:1, *v*/*v*) MTT viability assay, activity expressed as % cell survival relative to untreated control (100%) Human gastric epithelial cells (AGS) Non-digested extracts or fractions (extract concentrations: 13–50 μg/mL; maximum effect reported at 50 μg/mL): whole extract (73%), copigment fraction (73%), anthocyanin fraction (63%). MTT viability assay, activity expressed as % cell survival relative to untreated control (100%) Caco-2 human intestinal epithelial cells Bioaccessible (micellar) fraction of the digested whole fruit extracts (extract concentration 200 μg/mL): Plant extract: 51%	MTT viability assay Human gastric epithelial cells (AGS) Cyanidin-3-*O*-β-arabinoside (100 μM): 74%. MTT viability assay, activity expressed as % cell survival relative to untreated control (100%) Caco-2 human intestinal epithelial cells Monotropein (10 μM): 55%	[83]
*G. poeppigii* DC.	Methanol-formic acid (99:1, *v*/*v*) MTT viability assay, activity expressed as % cell survival relative to untreated control (100%) Human gastric epithelial cells (AGS) non-digested extracts or fractions (extract concentrations: 13–50 μg/mL; maximum effect reported at 50 μg/mL): whole extract (73%), copigment fraction (82%), anthocyanin fraction (no significant cytoprotective effect at 50 μg/mL). MTT viability assay, activity expressed as % cell survival relative to untreated control (100%) Caco-2 human intestinal epithelial cells Bioaccessible (micellar) fraction of the digested whole fruit extracts (extract concentration 200 μg/mL): Plant extract: 58%	MTT viability assay Human gastric epithelial cells (AGS) Cyanidin-3-*O*-β-arabinoside (100 μM): 74%. MTT viability assay, activity expressed as % cell survival relative to untreated control (100%) Caco-2 human intestinal epithelial cells Monotropein (10 μM): 55%	[83]
*G. procumbens* L.	Acetone extract Viability (membrane integrity), propidium iodide staining (flow cytometry), activity expressed as % of propidium iodide-positive (PI(+) cells; Human neutrophils Acetone extract: 2.2% PI(+) cells (extract concentration 25 μg/mL), 2.8% PI(+) cells (50 μg/mL), 3.2% PI(+) cells (100 μg/mL), 3.6% PI(+) cells (150 μg/mL)	Viability (membrane integrity) Positive control; Triton X-100: 98.6% PI(+) cells Negative control (untreated cells): 4.0% PI(+) cells	[85]
*G. tenuifolia* (Phil.) Sleumer	Polyphenol-enriched extract, anthocyanin fraction, copigment fraction MTT viability assay, activity expressed as % cell survival relative to untreated control (100%) Human gastric epithelial cells (AGS) Polyphenol-enriched extract: ~62% (extract concentration 6.25 μg/mL), ~68% (12.5 μg/mL), ~75% (25 μg/mL); anthocyanin fraction: ~55% (6.25 μg/mL), ~55% (12.5 μg/mL), ~56% (25 μg/mL); copigment fraction: ~68% (6.25 μg/mL), ~78% (12.5 μg/mL), ~84.1% (25 μg/mL)	MTT viability assay Human gastric epithelial cells (AGS) Cyanidin-3-*O*-β-arabinoside: ~70% (50 μg/mL), ~80% (100 μg/mL)	[96]
*G. trichophylla* Royle	Methanolic extract Brine shrimp lethality assay (*Artemia salina*) Concentration range: 1–750 µg/mL LC_50_ = 50 µg/mL	–	[97]
preliminary toxicity/safety assessment			
*G. erecta* Vent.	Ethanolic extract McLaughlin brine shrimp lethality assay Activity expressed as LC_50_ values (ppm): LC_50_ > 1000 ppm (non-toxic)	McLaughlin brine shrimp lethality assay *Bejaria resinosa* leaf extract: LC_50_ = 22 ppm	[68]
photoprotective activity			
*G. procumbens* L.	UVA-irradiated human Hs68 dermal fibroblasts cell viability after UVA irradiation; acetone extract: % of viable cells 99.5% (extract concentration 5 μg/mL), 105.3% (10 μg/mL), 109.3% (25 μg/mL); DNA damage; acetone extract: % of tail DNA 63.4% (5 μg/mL), 49.7% (10 μg/mL), 38.6% (25 μg/mL), 33.9% (50 μg/mL)	Cell viability after UVA irradiation; QU: 115.6% (25 μg/mL); AA: 120.4% (25 μg/mL); DNA damage; QU: 27.2% (25 μg/mL); AA: 33.9% (25 μg/mL)	[17,86]
antibacterial activity			
*G. procumbens* L.	Essential oil (hydrodistillation) Microdilution method Activity expressed as MIC (Minimum Inhibitory Concentration) values in mg/mL: *Staphylococcus aureus* (MIC = 13.5 mg/mL); *Enterococus faecalis* (15.3 mg/mL); *Escherichia coli* (8.8 mg/mL); *Acinetobacter baumanii* (8.2 mg/mL); Clinical strains and environmental bacteria Microdilution method Activity expressed as MIC (Minimum Inhibitory Concentration) values in mg/mL: *Staphylococcus aureus* (pus, MIC = 14.1 mg/mL); *Staphylococcus aureus* (pharynx, 13.2 mg/mL); *Enterococcus faecalis* (urine, 15.5 mg/mL); *Enterococcus faecalis* (respiratory, 16.4 mg/mL); *Escherichia coli* (wound, 9.7 mg/mL); *Escherichia coli* (bronchia, 8.8 mg/mL); *Acinetobacter baumanii* (sputum, 8.8 mg/mL); *Acinetobacter baumanii* (sink, 8.5 mg/mL)	Gentamicin; *S. aureus* (MIC = 0.016 mg/mL); *E. faecalis* (MIC = 0.064 mg/mL); *E. coli* (MIC = 0.004 mg/mL); *A. baumanii* (MIC = 0.032 mg/mL)	[17,87]
*G. trichophylla* Royle	Agar well diffusion and MIC determination Activity expressed as zone of inhibition (extract concentrations 7.5, 15, 30 mg/mL) or MIC (mg/mL) values: *Bacillus subtilis*: methanolic extract (MIC = 15 mg/mL), ethyl acetate extract (7.5 mg/mL), *n*-hexane extract (>30 mg/mL), chloroform extract (30 mg/mL), *n*-butanol extract (7.5 mg/mL), water extract (15 mg/mL); *Staphylococcus aureus*: methanolic extract (7.5 mg/mL), ethyl acetate extract (7.5 mg/mL), *n*-hexane extract (7.5 mg/mL), chloroform extract (7.5 mg/mL), *n*-butanol extract (7.5 mg/mL), water extract (7.5 mg/mL); *Escherichia coli*: methanolic extract (15 mg/mL), ethyl acetate extract (7.5 mg/mL), *n*-hexane extract (30 mg/mL), chloroform extract (7.5 mg/mL), *n*-butanol extract (7.5 mg/mL), water extract (7.5 mg/mL); *Pseudomonas aeruginosa*: methanolic extract (7.5mg/mL), ethyl acetate extract (7.5 mg/mL), *n*-hexane extract (7.5 mg/mL), chloroform extract (7.5 mg/mL), *n*-butanol extract (7.5 mg/mL), water extract (7.5 mg/mL)	Roxithromycin (1 mg/mL), Cefixime (1 mg/mL) MIC ≤ 1 mg/mL	[97]
antifungal activity			
*G. trichophylla* Royle	Agar tube dilution method Concentrations tested: 5, 10, 15 mg/mL Activity expressed as % of growth inhibition: *Helminthosporium solani*: methanolic extract (71%), ethyl acetate extract (58.5%), *n*-hexane extract (48.5%), chloroform extract (57%), *n*-butanol extract (53%), water extract (68%); *Fusarium solani*: methanolic extract (58%), ethyl acetate extract (72.5%), *n*-hexane extract (70%), chloroform extract (67.5%), *n*-butanol extract (65.5%), water extract (72%); *Aspergillus flavus*: methanolic extract (78%), ethyl acetate extract (29%), *n*-hexane extract (35%), chloroform extract (37%), *n*-butanol extract (37%), water extract (30.5%); *Aspergillus fumigatus*: methanolic extract (59%), ethyl acetate extract (61.5%), *n*-hexane extract (53.5%), chloroform extract (44%), *n*-butanol extract (55%), water extract (53%)	Terbinafine; *Helminthosporium solani* (86.33%), *Fusarium solani* (77.3%), *Aspergillus flavus* (84%), *Aspergillus fumigatus* (86.66%)	[97]
antischistosomal activity			
*G. mucronata* (L.f.) Hook. & Arn.	Essential oil (hydrodistillation) In vivo mouse infection model; mice infected with *Schistosoma mansoni* Dose of essential oil: 5 mg/kg Effect: 67% reduction in worm burden, 9/10 oogram alteration (severe disruption of parasite egg development)	The results were compared against untreated infected control animals	[77]
dermatological activity			
*G. erecta* Vent.	Methanolic extract (different maturation states: immature, intermediate, mature fruits) Activity expressed in μg/mL of the extract: Collagenase inhibition: 100–100% (extract concentration 500 μg/mL); Elastase inhibition: 96.6–99.9% (500 μg/mL); Hyaluronidase inhibition:78.0–100% (500 μg/mL); Tyrosinase inhibition: 90.0–93.0% (extract concentration 500 μg/mL)	Collagenase inhibition: oleanolic acid 92.9% (250 μM); *Camelia sinensis* 100% (extract concentration 500 μg/mL); Elastase inhibition: oleanolic acid 80.4% (500 μM); *Camelia sinensis* 66.2% (500 μg/mL); Hyaluronidase inhibition: EGCG 69.3% (250 μM); *Camelia sinensis* 2.2% (500 μg/mL); Tyrosinase inhibition: Kojic acid 98.5% (500 μM); *Camelia sinensis* 81.5% (extract concentration 500 μg/mL)	[67]
*G. procumbens* L.	Activity expressed in μg/mL of the extract: Hyaluronidase inhibition; acetone extract: IC_50_ = 28.39 μg/mL; methanol-water (75:25, *v*/*v*): IC_50_ = 32.72 μg/mL; *n*-butanol extract: IC_50_ = 49.30 μg/mL; water extract: IC_50_ = 39.34 μg/mL	Hyaluronidase inhibition; IND: IC_50_ = 12.77 μg/mL; DEX: 14.18 μg/mL	[17,85]
*G. pumila* (L.f.) D.J.Middleton	Methanol-formic acid (98:2, *v*/*v*) Activity expressed in μg/mL of the extract: Tyrosinase inhibition: IC_50_ = 3.3 μg/mL	Tyrosinase inhibition; Kojic acid: IC_50_ = 10.0 μg/mL	[90]
neuroprotective activity			
*G. pumila* (L.f.) D.J.Middleton	Methanol-formic acid (98:2, *v*/*v*) Activity expressed in μg/mL of the extract: Acetylcholinesterase inhibition: IC_50_ = 7.7 μg/mL; Butyrylcholinesterase inhibition: IC_50_ = 34.5 μg/mL	Acetylcholinesterase inhibition; galanthamine: IC_50_ = 0.3 μg/mL; Butyrylcholinesterase inhibition; galantamine: IC_50_ = 3.82 μg/mL	[90]
antiproliferative activity			
*G. pumila* (L.f.) D.J.Middleton	Methanol-formic acid (98:2, *v*/*v*) Sulforhodamine B (SRB) assay Concentrations tested: 2.5–250 μg/mL Cell lines: A549 (lung), HBL-100 (breast), HeLa (cervix), SW1573 (lung), T-47D (breast), WiDr (colon) Activity expressed as GI_50_ (50% growth inhibition) and TGI (total growth inhibition) in μg/mL of the extract: For all six cancer cell lines: GI_50_ > 250 µg/mL, TGI > 250 µg/mL	DMSO = negative control (no cytotoxic reference drug used)	[90]

AA: ascorbic acid; CA: (+)-catechin; DEX: dexamethasone; ECA: (–)-epicatechin; EGCG: epigallocatechin gallate; GA: gallic acid; IND: indomethacin; QU: quercetin; TX: Trolox^®^ (6-hydroxy-2,5,7,8-tetramethylchroman-2-carboxylic acid); ORAC: Oxygen Radical Absorbance Capacity; TEAC: Trolox Equivalent Antioxidant Activity; CUPRAC: Cupric Reducing Antioxidant Capacity; DPPH: DPPH^•^ radical scavenging test; ABTS: ABTS^•+^ radical cation scavenging test; FRAP: antioxidant activity expressed in millimoles of Fe^2+^ ions/g of tested compound (Ferric Reducing Antioxidant Power); TBARS: linoleic acid oxidation inhibition test that measures the level of thiobarbituric acid reactive substances produced; O_2_^•–^: superoxide radical anion scavenging test; OH^•^: hydroxyl radical scavenging test; H_2_O_2_: hydrogen peroxide reduction test; ROS: Reactive Oxygen Species; IL: interleukin; TNF-α: tumour necrosis factor-α (Tumour Necrosis Factor-α); MMP-9: matrix metalloproteinase-9; ELA-2: human elastase-2; ICAM-1: Intercellular Adhesion Molecule 1; NF-κB: Nuclear Factor kappa-light-chain-enhancer of activated B cells; Erk: Extracellular signal-regulated kinase; LPS: bacterial lipopolysaccharide obtained from *Escherichia coli* O111:B4; *f*MLP: *N*-formyl-L-methionyl-L-leucyl-L-phenylalanine; IC_50_: concentration of a tested extract or reference substance, expressed in μg/mL or mg/mL, which reduces the enzyme activity or inhibits the oxidation of linolenic acid by 50% (*Inhibitory Concentration*); dw: dry weight; fw: fresh weight.

**Table 4:** Critical assessment of the level and strength of evidence supporting biological activities of *Gaultheria* fruits.

Activity	Evidence Type	Model	Main Finding	Positive Control	Major Methodological Limitations	Evidence Strength
Antioxidant	Primarily in vitro cell-free; selected cellular studies	DPPH, ABTS/TEAC, FRAP, ORAC, CUPRAC, TBARS and ROS-scavenging assays; AAPH-treated Caco-2 cells; stimulated human neutrophils; UVA-exposed Hs68 fibroblasts	Antioxidant effects were repeatedly demonstrated across several species; enriched phenolic/anthocyanin fractions generally showed the strongest activity; selected extracts also reduced cellular oxidative stress and modulated antioxidant defence pathways	Trolox, quercetin, ascorbic acid, gallic acid and other assay-specific standards; absent in some studies	Predominance of non-physiological chemical assays; substantial heterogeneity of extraction procedures, units and assay conditions; some studies without positive controls; no direct in vivo or human confirmation specifically for antioxidant effects of the fruits	Moderate
Anti-inflammatory	In vitro enzyme-based and cellular	Hyaluronidase, lipoxygenase and COX-related assays; stimulated human neutrophils and Hs68 dermal fibroblasts	Selected extracts, particularly from *G. procumbens*, inhibited inflammatory enzymes and reduced ROS and inflammatory mediators/signalling, including IL-1β, TNF-α, IL-8, NF-κB, ICAM-1 and Erk-related responses	Dexamethasone, indomethacin and other assay-dependent references; appropriate controls present in principal cellular studies	Predominantly in vitro evidence; different models and endpoints between studies; limited replication across species; lack of pharmacokinetic, animal and human validation	Moderate
Photoprotective	In vitro cellular	UVA-irradiated human Hs68 dermal fibroblasts	*G. procumbens* fruit extract preserved cell viability, decreased ROS and DNA damage, and supported endogenous antioxidant defences following UVA exposure	Quercetin and ascorbic acid	Evidence essentially restricted to one species and one cellular model; no skin penetration, ex vivo skin, animal or clinical photoprotection studies	Low–moderate
Cytoprotective/gastrointestinal protection	In vitro cellular, including simulated gastrointestinal digestion	AGS gastric epithelial and Caco-2 intestinal cells; non-digested extracts/fractions and bioaccessible micellar fractions	Extracts/fractions of *G. phillyreifolia*, *G. poeppigii* and *G. tenuifolia* showed preservation of epithelial-cell viability; digested fractions enabled assessment under more physiologically relevant gastrointestinal conditions	Selected isolated compounds/reference conditions; not consistently accompanied by a pharmacological positive control	MTT-based endpoints provide limited mechanistic information; relatively high experimental concentrations; absence of animal gastric/intestinal injury models and human studies	Low–moderate
Anti-diabetic/glucose-modulating	In vitro enzyme-based and cellular; some simulated gastrointestinal digestion	α-Glucosidase/α-amylase inhibition; glucose uptake and transporter-expression studies in Caco-2/Caco-2/15 cells	*G. phillyreifolia* and *G. poeppigii* extracts strongly inhibited α-glucosidase and reduced intestinal glucose uptake and expression of glucose-transport-related genes; *G. hispidula* also reduced glucose uptake	Acarbose; quercetin in selected cellular experiments	No controlled animal or human glycaemic studies; intestinal cell lines do not reproduce whole-body glucose homeostasis; uncertainty regarding achievable dietary concentrations and systemic exposure	Moderate
Neuroprotective/cholinesterase inhibitory	In vitro cell-free enzyme screening	Acetylcholinesterase and butyrylcholinesterase inhibition	*G. pumila* extract inhibited AChE (IC_50_ 7.7 μg/mL) and BChE (IC_50_ 34.5 μg/mL)	Galantamine	Evidence derived from a single species and isolated enzyme assays; extract less potent than reference drug; no neuronal-cell, brain-exposure, animal or clinical evidence	Low
Dermatological/anti-tyrosinase and extracellular-matrix enzyme inhibition	In vitro enzyme-based	Mushroom tyrosinase; collagenase, elastase and hyaluronidase inhibition	Strong tyrosinase inhibition was reported for *G. pumila* and *G. erecta*; *G. erecta* also showed marked collagenase, elastase, and hyaluronidase inhibition	Kojic acid, *Camellia sinensis* extract, oleanolic acid, EGCG	Mostly isolated enzyme assays; some results obtained at a single relatively high extract concentration; lack of human melanocyte/skin-equivalent, dermal penetration, and clinical studies	Low
Antiproliferative/anticancer-oriented	In vitro cellular	SRB assay in A549, HBL-100, HeLa, SW1573, T-47D and WiDr tumour cell lines	*G. pumila* extract showed no marked antiproliferative activity; GI_50_ and TGI were >250 μg/mL in all six cancer cell lines	DMSO negative control; no cytotoxic reference drug	Only one species investigated; no active positive control; no meaningful antiproliferative effect within tested concentrations; no mechanistic, selectivity, animal, or clinical evidence	Very low/insufficient evidence for activity
Antibacterial/antifungal	In vitro microbiological	Broth microdilution and fungal growth-inhibition assays	*G. procumbens* fruit essential oil and *G. trichophylla* extracts inhibited selected Gram-positive, Gram-negative, and fungal strains, although effective MICs were generally in the mg/mL range	Antibiotic/antifungal references in selected assays; inconsistent across studies	Relatively high MICs; limited number of studies/species; heterogeneous extracts; limited data on bactericidal activity, time-kill kinetics, biofilms or resistance; no infection-model validation	Low
Antiparasitic/antischistosomal	In vivo animal	*Schistosoma mansoni*-infected mice	*G. mucronata* fruit essential oil at 5 mg/kg reduced worm burden by approximately 67% and markedly altered parasite egg development	Untreated infected animals; no standard antischistosomal drug reported as comparator	Single animal study; small and incompletely replicated evidence base; absence of dose–response, pharmacokinetic and confirmatory studies; no human evidence	Moderate, but preliminary

## Data Availability

The data presented in this study are available on request from the corresponding author.

## Supplementary Materials

The following supporting information can be downloaded at https://www.mdpi.com/article/10.3390/nu18183015/s1. Table S1: The chemical composition of *Gaultheria* fruits.

## Abbreviations

The following abbreviations are used in this manuscript:

AA	ascorbic acid
AAE	ascorbic acid equivalents
ABTS	ABTS^•+^ radical cation scavenging test
CA	(+)-catechin
CAE	(+)-catechin equivalents
CGE	cyanidin-3-glucoside equivalents
CUPRAC	Cupric Reducing Antioxidant Capacity
CYE	cyanidin chloride equivalents
DEX	dexamethasone
DMAC	4-dimethylaminocinnamaldehyde assay
DPPH	DPPH^•^ radical scavenging test
dw	dry weight
ECA	(–)-epicatechin
EGCG	epigallocatechin gallate
ELA-2	human elastase-2
Erk	Extracellular signal-regulated kinase
*f*MLP	*N*-formyl-L-methionyl-L-leucyl-L-phenylalanine
FRAP	antioxidant activity expressed in millimoles of Fe^2+^ ions/g of tested compound (Ferric Reducing Antioxidant Power)
fw	fresh weight
GA	gallic acid
H_2_O_2_	hydrogen peroxide
ICAM-1	Intercellular Adhesion Molecule 1
IND	indomethacin
IL	interleukin
LPS	bacterial lipopolysaccharide from *Escherichia coli* O55:B5
MMP-9	matrix metalloproteinase-9
NF-κB	Nuclear Factor kappa-light-chain-enhancer of activated B cells
O_2_^•–^	superoxide anion radical
^•^OH	hydroxyl radical
ORAC	Oxygen Radical Absorbance Capacity
QE	quercetin equivalents
QU	quercetin
ROS	Reactive Oxygen Species
TAE	tannic acid equivalents
TBARS	linoleic acid oxidation inhibition test that measures the level of thiobarbituric acid reactive substances produced
TEAC	Trolox Equivalent Antioxidant Activity
TNF-α	tumour necrosis factor-α
TPC	total phenolic content expressed in gallic acid equivalents (mg GAE/g)
TX	Trolox^®^ (6-hydroxy-2,5,7,8-tetramethylchroman-2-carboxylic acid)
