# Supplementary material for: Gaultheria Berries as Potential Functional Foods: Phytochemical Composition, Health-Promoting Properties, and Nutritional Potential
Source: Nutrients. 2026 Sep 15;18(18):3015. doi: 10.3390/nu18183015 (PMC13609934; doi:10.3390/nu18183015)
Supplement: Supplementary file 1 [file nutrients-18-03015-s001.zip › nutrients-4502061-supplementary.pdf]

Supplementary Materials

Gaultheria Berries as Potential Functional Foods: Phytochemical Composition, Health-Promoting Properties, and Nutritional Potential

Piotr Michel

Department of Pharmacognosy, Faculty of Pharmacy, Medical University of Lodz, Muszyńskiego 1, 90-151 Lodz, Poland; piotr.michel@umed.lodz.pl; Tel.: +48-426779169

Table S1. The chemical composition of *Gaultheria* fruits.

Plant	Compound	Method	Extract/Content	References
G. antarctica Hook.f.	Anthocyanins: delphinidin-3-galactoside, delphinidin-pentoside, cyanidin-3-galactoside, cyanidin-3-glucoside, cyanidin-pentoside	HPLC-DAD-ESI-MS/MS<br>Spectrophotometric method<br>HPTLC	Methanol-formic acid (97:3, *v/v*)<br>Content in μmol/g or mg/100 g fw of the fruits:<br>total anthocyanins (HPLC-DAD): 0.19 μmol/g fw;<br>monomeric anthocyanins (pH-differential method): 0.09 μmol CGE/g fw;<br>ascorbic acid (HPTLC): 85.6 mg/100 g fw.<br><br>Content (HPLC-DAD) in μmol/g fw of the fruits: cyanidin-pentoside (0.05 μmol/g fw), delphinidin-3-galactoside (0.06 μmol/g fw), cyanidin-3-galactoside+delphinidin-pentosied (0.09 μmol/g fw), total anthocyanins (0.19 μmol/g fw)	[1,2]
	Flavonoids: myricetin hexoside, myricetin pentoside, quercetin 3-galactoside, quercetin pentoside, quercetin 3-rhamnoside; Coumaroyl-iridoid	HPLC-DAD-ESI-MS/MS	Methanol-water (97:3, *v/v*) after SPE<br>Content in μmol/g fw of the fruits:<br>hydroxycinnamic acid derivatives (HPLC-DAD): n.d.;<br>total flavonols (HPLC-DAD): 0.05 μmol/g fw;<br>coumaroyl-iridoid (HPLC-DAD): 0.26 μmol/g fw	[3]
G. erecta Vent.	–	Spectrophotometric method	Methanolic extract (different maturation states: immature, intermediate, mature fruits)<br>Content in mg/g dw of the extract:<br>TPC (Folin–Ciocalteu assay): 306.4-881.3 mg GAE/g dw	[4]
	Colour-precipitation reactions: flavonoids, anthocyanins, quinones, tannins, coumarins, terpenes, steroids	Spectrophotometric methods	Ethanolic extract<br>Content in mg/g dw of the extract:<br>TPC (Folin–Ciocalteu assay): 105.6 mg GAE/g dw;<br>total flavonoids ($AlCl_3$ colorimetric assay): 90.7 mg QE/g dw	[5]
G. fragrantissima Wall.	–	Spectrophotometric methods	Methanol-water (70:30, *v/v*)<br>Content in mg/100 g fw of the fruits:<br>TPC (Folin–Ciocalteu assay): 80.4 mg GAE/100 g fw;<br>total anthocyanins (pH-differential method): 2.47 mg CGE/100 g fw;<br>total flavonoids ($AlCl_3$ colorimetric assay): 94.3 mg QE/100 g fw;<br>ascorbic acid (colorimetric assay with Fe^{3+} reduction): 67.6 mg AAE/100 g fw	[6]
	Essential oil components: methyl salicylate, linalool, α-terpineol, β-caryophyllene, α-	GC-MS	Essential oil (hydrodistillation)	[7]

	pinene, limonene, germacrene D, β-phellandrene, camphene, camphor			
G. glomerata (Cav.) Sleumer	–	Spectrophotometric methods<br>2,6-dichloroindophenol titration method	Ethanolic extract<br>Content in mg/100 g fw or dw of the fruits:<br>TPC (Folin–Ciocalteu assay): 344.37 mg GAE/100 g fw, 2117.91 mg GAE/100 g dw.<br><br>Ethanol-water (80:20, *v*/*v*)<br>Content in mg/100 g fw or dw of the fruits:<br>total monomeric anthocyanins (pH-differential method): 112.88 mg CGE/100 g fw, 694.20 mg/100 g dw;<br>ascorbic acid: 13.87 mg AAE/100 g fw, 85.20 mg AAE/100 g dw	[8]
	Anthocyanins: delphinidin, cyanidin	HPLC-DAD<br>Spectrophotometric method	Methanolic extract acidified with 0.01% HCl<br>Content in mg/100 g fw of the fruits:<br>TPC (Folin–Ciocalteu assay): 1804.07 mg GAE/100 g fw (fresh), 1585.49 mg GAE/100 g fw (frozen), 820.73 mg GAE/100 g fw (dried);<br>total anthocyanins (pH-differential method): 19.15 mg CGE/100 g fw (fresh), 21.71 mg CGE/100 g fw (frozen), 12.92 mg CGE/100 g fw (dried)	[9]
	Colour-precipitation reactions: flavonoids, cardiotonic glycosides, phenols and/or tannins, anthocyanins, coumarins	Spectrophotometric method	Ethanol-water (70:30, *v*/*v*) acidified with citric acid (pH = 3.3)<br>Content in mg/100 g dw of the fruits:<br>total anthocyanins (pH-differential method): 2340 mg CGE/100 g dw	[10]
	–	Spectrophotometric method	Ethanol-1% citric acid<br>Content in mg/L of the extract:<br>total anthocyanins (pH-differential method): 224.14 mg CGE/L	[11]
	Methanol-1% hydrochloric acid<br>Anthocyanins: cyanidin-3-galactoside, peonidin-3-galactoside, malvidin-3-glucoside, delphinidin-3-glucoside, delphinidin-3-galactoside, cyanidin-3-glucoside, cyanidin-3-arabinoside, peonidin-3-arabinoside	HPLC-DAD<br>Spectrophotometric methods<br>Atomic Absorption Spectroscopy (AAS)	Ethanol-1% citric acid<br>Content in mg/100 mL of the extract:<br>TPC (Folin–Ciocalteu assay): 3503 mg GAE/100 mL;<br>total anthocyanins (pH-differential method): 879.0 mg CGE/100 mL;<br>AAS; calcium: 3.86 mg/100 g, iron: 0.29 mg/100 g, magnesium: 0.69 mg/100 g, potassium: 47.25 mg/100 g, sodium: 2.22 mg/100 g, zinc: 0.15 mg/100 g.<br><br>Methanol-1% hydrochloric acid<br>HPLC-DAD (relative content, % of total anthocyanins): cyanidin-3-galactoside (22.0%); peonidin-3-galactoside (21.0%), malvidin-3-	[12]

			glucoside (6.4%), delphinidin-3-glucoside (6.8%), delphinidin-3-galactoside (2.7%), cyanidin-3-glucoside (1.0%), cyanidin-3-arabinoside (0.3%), peonidin-3-arabinoside (1.0%)	
	Proteins (Kjeldahl nitrogen determination): 1.29-1.49%; Fat (Soxhlet extraction): 0.99-1.17%; Carbohydrates (by difference): 6.17-6.34%; Moisture (oven-drying to constant weight): 74.3-89.5%; Ash (incineration in a muffle furnace): 1.72-1.88%	Spectrophotometric method	Functional beverage combining fruit juice of *G. glomerata* with stem juice of *Oxalis tuberosa* (different formulations: B0-B3)<br>Content in mg/100 g of the fresh juice:<br>TPC (Folin–Ciocalteu assay): 1783-1825 mg GAE/100 g	[13]
G. mucronata (L.f.) Hook. & Arn.	<u>Anthocyanins</u>: delphinidin-3-galactoside, cyanidin-3-lathyroside, delphinidin-pentoside, cyanidin-3-galactoside, cyanidin-3-glucoside, cyanidin-pentoside, cyanidin-dipentoside	HPLC-DAD-ESI-MS/MS<br>Spectrophotometric method<br>HPTLC	Methanol-formic acid (97:3, *v/v*)<br>Content in μmol/g or mg/100 g fw of the fruits:<br>total anthocyanins (HPLC-DAD): 1.23 μmol/g fw;<br>monomeric anthocyanins (pH-differential method): 0.83 μmol CGE/g fw;<br>ascorbic acid (HPTLC): 127.4 mg/100 g fw.<br><br>Content (HPLC-DAD) in μmol/g fw of the fruits: cyanidin-pentoside (0.77 μmol/g fw), delphinidin-pentosied+cyanidin-3-galactoside (0.46 μmol/g fw), total anthocyanins (1.23 μmol/g fw)	[1,2]
	<u>Caffeoylquinic acid isomers</u>: 3-caffeoylquinic acid, 5-caffeoylquinic acid, caffeoylquinic acid;<br><u>Coumaroyl-iridoid</u>;<br><u>Flavonoids</u>: quercetin glucuronide, quercetin xylopentoside, quercetin 3-rhamnoside	HPLC-DAD-ESI-MS/MS	Methanol-water (97:3, *v/v*) after SPE<br>Content in μmol/g or mg/100 g fw of the fruits:<br>hydroxycinnamic acid derivatives (HPLC-DAD): 0.57 μmol/g fw;<br>total flavonols (HPLC-DAD): 0.32 μmol/g fw;<br>coumaroyl-iridoid (HPLC-DAD): 0.64 μmol/g fw	[3]
	<u>Phenolic compounds</u>: *p*-coumaric acid, ferulic acid;<br><u>Terpenoids</u>: ursolic acid, α-amyrin, β-amyrin	GC-MS after BSTFA silylation	Cuticular (surface) wax; chloroform and hexane-ethyl acetate (1:1, *v/v*)<br>Content in g/100 g dw of the wax extract:<br><u>Phenolic compounds</u>: *p*-coumaric acid (0.173 g/100 g dw), ferulic acid (0.199 g/100 g dw), syringic acid (nd), methyl caffeate (nd), total phenolic acids (0.372 g/100 g dw);<br><u>Terpenoids</u>: ursolic acid (4.379 g/100 g dw), α-amyrin (1.958 g/100 g dw), β-amyrin (0.956 g/100 g dw), betulinic acid (nd), total terpenoids (7.804 g/100 g dw)	[14]
	<u>Essential oil components</u>: *o*-cimene, cadinene, farnesene, germacrene, germacrol, bisabolol, apiol, estragole, carveol, thymol, menthofuran, chrysanthenone epoxide, chrysomelidial, 2-rossalene, precapnelladiene	GC-MS	Essential oil (hydrodistillation)	[15]

G. myrsinoides Kunth	Colour-precipitation reactions: flavonoids, cardiotonic glycosides, phenols and/or tannins, anthocyanins, coumarins	Spectrophotometric method	Ethanol-water (70:30, *v*/*v*) acidified with citric acid (pH = 3.3)<br>Content in mg/100 g dw of the fruits:<br>total anthocyanins (pH-differential method): 1655.2 mg CGE/100 g dw	[10]
	–	Tillman's method (titration assay)	Extract unknown<br>Content in mg/100 g fw of the fruits:<br>ascorbic acid: 19.02 mg/100 g fw	[16]
	Colour-precipitation reactions: tannins, flavonoids, triterpenes, and/or steroids	Spectrophotometric method	Ethanolic extract<br>Content in µg/mg dw of the extract:<br>TPC (Folin–Ciocalteu assay): 6.08 µg GAE/mg dw	[17]
G. phillyreifolia (Pers.) Sleumer	Anthocyanins: delphinidin-3-galactoside, cyanidin-dihexoside, cyanidin-3-galactoside, delphinidin-3-arabinoside, cyanidin-hexoside, cyanidin-3-arabinoside;<br>Caffeoylquinic acid isomers: 5-caffeoylquinic acid, 3-caffeoylquinic acid;<br>Quinic acid isomers: 5-*p*-coumaroylquinic acid;<br>Proanthocyanidins: procyanidin A-type trimer, procyanidin B-type dimer 1, procyanidin B-type dimer 2;<br>Simple phenolic acids: sinapic acid hexoside, sinapic acid hexoside derivative;<br>Flavonoids: myricetin hexoside, myricetin pentoside 1, myricetin pentoside 2, myricetin rhamnoside, quercetin 3-galactoside, quercetin pentoside 1, quercetin arabinoside, quercetin rhamnoside, isorhamnetin rhamnoside, kaempferol rhamnoside, quercetin;<br>Iridoids: *p*-coumaroyl monotropein isomer 1, monotropein-10-*trans*-*p*-coumarate (vaccinoside), *p*-coumaroyl dihydromonotropein isomer 1, *p*-coumaroyl dihydromonotropein isomer 2, 6*α*-hydroxy-dihydromonotropein-10-*trans*-cinnamate, monotropein-10-*trans*-cinnamate, dihydromonotropein cinnamate;<br>Other compounds: coumaroyl hexoside, caffeoylshikimic acid.	HPLC-DAD-ESI-MS^n<br>Spectrophotometric methods<br>isolation; CCC, HR-MS, ^1H and ^{13}C NMR	Methanol-formic acid (99:1, *v*/*v*) (different colours of the fruits, and time and place of collection)<br>Content in mg/g dw of the extract:<br>TPC (Folin–Ciocalteu assay): 147.5-290.3 mg GAE/g dw;<br>total flavonoids ($AlCl_3$ colorimetric assay): 97.2-184.4 mg CAE/g dw;<br>total anthocyanins (pH-differential method): 26.2-87.5 mg CGE/g dw;<br>total proanthocyanidins (DMAC assay): 18.2-69.3 mg CAE/g dw;<br><br>Content of individual compounds in mg/100 g fw of the fruits:<br>Anthocyanins (HPLC-DAD): delphinidin-3-galactoside (2.2-49.5 mg/100 g fw), cyanidin-3-galactoside (4.0-158.5 mg/100 g fw), delphinidin-3-arabinoside (8.6-48.5 mg/100 g fw), cyanidin-3-galactoside (17.4-131.2 mg/100 g fw), total anthocyanins (32.3-293.8 mg/100 g fw);<br>Hydroxycinnamic acids (HPLC-DAD): 5-caffeoylquinic acid (1.6-3.3 mg/100 g fw), 3-caffeoylquinic acid (0.7-16.6 mg/100 g fw), caffeoylshikimic acid (6.3-25.1 mg/100 g fw), total hydroxycinnamic acids (0.7-28.8 mg/100 g fw);<br>Flavonols (HPLC-DAD): myricetin hexoside (4.0-8.2 mg/100 g fw), myricetin rhamnoside (1.1-17.5 mg/100 g fw), quercetin galactoside (14.9-40.8 mg/100 g fw), quercetin glucuronide (5.4-24.6 mg/100 g fw), quercetin pentoside (4.8-6.4 mg/100 g fw), quercetin arabinoside (2.9-8.7 mg/100 g fw), quercetin rhamnoside (8.9-89.4 mg/100 g fw), quercetin (1.7-12.1 mg/100 g fw), total flavonols (46.4-163.6 mg/100 g fw);<br>Coumaroyl iridoids (HPLC-DAD): *p*-coumaroyl monotropein (3.1-7.4 mg/100 g fw), monotropein 10-*trans*-*p*-coumarate (vaccinoside; 14.3-108.2 mg/100 g fw), 6*α*-hydroxy-	[2,18,19]

	Isolation and structure elucidation: monotropein-10-*trans*-*p*-coumarate (vaccinoside), 6*α*-hydroxy-dihydromonotropein-10-*trans*-cinnamate, monotropein-10-*trans*-cinnamate		dihydromonotropein-10-*trans*-cinnamate (118.4-452.2 mg/100 g fw), monotropein-10-*trans*-cinnamate (186.5-834.5 mg/100 g fw), total iridoids (359.6-1334.1 mg/100 g fw)	
	Simple phenolic acids: protocatechuic acid hexoside;<br>Caffeoylquinic acid isomers: 5-caffeoylquinic acid, 3-caffeoylquinic acid;<br>Anthocyanins: cyanidin-3-*O*-galactoside, cyanidin-3-*O*-arabinoside;<br>Flavonoids: myricetin hexoside, quercetin 3-*O*-galactoside, quercetin 3-*O*-glucuronide, quercetin 3-*O*-glucoside, myricetin glucuronide, quercetin pentoside, quercetin 3-*O*-arabinoside, kaempferol rhamnoside, quercetin;<br>Iridoids: caffeoyl monotropein, coumaroyl monotropein isomer, monotropein 10-*trans*-*p*-coumarate, 6*α*-hydroxy-dihydromonotropein-10-*trans*-cinnamate, monotropein-10-*trans*-cinnamate, dihydromonotropein cinnamate;<br>Other compounds: 5-*O*-caffeoylshikimic acid	UHPLC-DAD-ESI-MS<br>HPLC-DAD	Methanol-formic acid (99:1, *v*/*v*)<br>Content in mg/g dw of the non-digested and after in vitro oral, gastric, and intestinal digestion extract:<br>Anthocyanins (HPLC-DAD): cyanidin-3-*O*-galactoside (non-digested: 31.3 mg/g dw; other: 0.3-29.9 mg/g dw), cyanidin-3-*O*-arabinoside (non-digested: 32.2 mg/g dw; other: 0.4-30.6 mg/g dw), total anthocyanins (non-digested: 63.5 mg/g dw; other: 0.7-60.5 mg/g dw);<br>Hydroxycinnamic acids (HPLC-DAD): 5-*O*-caffeoylquinic acid (non-digested: 2.9 mg/g dw; other: 0.8-2.1 mg/g dw), 3-*O*-caffeoylquinic acid (non-digested: 4.3 mg/g dw; other: 2.1-4.2 mg/g dw), 5-*O*-caffeoylshikimic acid (non-digested: 12.3 mg/g dw; other: 6.0-11.9 mg/g dw), total hydroxycinnamic acids (non-digested: 19.5 mg/g dw; other: 8.9-18.2 mg/g dw);<br>Flavonols (HPLC-DAD): myricetin hexoside (non-digested: 2.5 mg/g dw; other: 1.0-2.3 mg/g dw), quercetin 3-*O*-galactoside (non-digested: 33.9 mg/g dw; other: 18.0-37.1 mg/g dw), quercetin 3-*O*-glucuronide (non-digested: 0.9 mg/g dw; other: 0.7-1.1 mg/g dw), quercetin 3-*O*-glucoside (non-digested: 1.3 mg/g dw; other: 0.6-1.6 mg/g dw), quercetin 3-*O*-arabinoside (non-digested: 2.3 mg/g dw; other: 1.2-2.6 mg/g dw), quercetin 3-*O*-rhamnoside (non-digested: 60.5 mg/g dw; other: 32.3-65.5 mg/g dw), quercetin (non-digested: 2.6 mg/g dw; other: 1.6-6.0 mg/g dw), total flavonols (non-digested: 104.0 mg/g dw; other: 55.4-116.0 mg/g dw);<br>Monotropein iridoids (HPLC-DAD): monotropein 10-*trans*-*p*-coumarate (non-digested: 32.7 mg/g dw; other: 22.2-31.3 mg/g dw), 6*α*-hydroxy-dihydromonotropein-10-*trans*-cinnamate (non-digested: 179.0 mg/g dw; other: 110.0-185.0 mg/g dw), monotropein-10-*trans*-cinnamate (non-digested: 404.0 mg/g dw; other: 222.0-400.0 mg/g dw), dihydromonotropein cinnamate (non-digested: 8.1 mg/g dw; other: 1.6-8.6 mg/g dw), total monotropein iridoids (non-digested: 624.0 mg/g dw; other: 356.0-625.0 mg/g dw)	[20]
	–	HPLC-DAD<br>HPLC-FL	Methanol-formic acid (99:1, *v*/*v*)<br>Content in μg or mg/100 g fw of the fruits:	[21]

			Carotenoids (HPLC-DAD):<br>lutein (55.69 μg/100 g fw); zeaxanthin (3.76 μg/100 g fw); β-cryptoxanthin (0.21 μg/100 g fw); lycopene (0.74 μg/100 g fw); α-carotene (0.19 μg/100 g fw); β-carotene (13.32 μg/100 g fw); total carotenoids (73.91 μg/100 g fw);<br>Tocochromanols (HPLC-FL):<br>α-tocopherol (0.12 mg/100 g fw); γ-tocopherol (0.036 mg/100 g fw); δ-tocopherol (0.006 mg/100 g fw); α-tocotrienol (nd); β-tocotrienol (nd); γ-tocotrienol (0.107 mg/100 g fw); δ-tocotrienol (0.01 mg/100 g fw); total tocochromanols (0.279 mg/100 g fw);<br>ascorbic acid (HPLC-DAD): 3.08 mg/100 g fw	
G. poeppigii DC.	<u>Anthocyanins</u>: delphinidin-3-galactoside, cyanidin-3-galactoside, delphinidin-3-arabinoside, cyanidin-3-arabinoside;<br><u>Caffeoylquinic acid isomers</u>: 5-caffeoylquinic acid, 3-caffeoylquinic acid, caffeoylquinic acid isomer;<br><u>Proanthocyanidins</u>: procyanidin B-type trimer 1;<br><u>Simple phenolic acids</u>: dihydroxybenzoic acid hexoside, sinapic acid hexoside, sinapic acid hexoside derivative;<br><u>Flavonoids</u>: myricetin glucuronide, myricetin hexoside, myricetin pentoside 1, myricetin pentoside 2, quercetin 3-galactoside, quercetin rhamnoside, kaempferol rhamnoside, quercetin;<br><u>Iridoids</u>: *p*-coumaroyl monotropein isomer 1, monotropein-10-*trans*-*p*-coumarate (vaccinoside), *p*-coumaroyl dihydromonotropein isomer 1, *p*-coumaroyl dihydromonotropein isomer 2, 6α-hydroxy-dihydromonotropein-10-*trans*-cinnamate, *p*-coumaroyl monotropein derivative 1, *p*-coumaroyl monotropein derivative 2, monotropein-10-*trans*-cinnamate;<br><u>Other compounds</u>: coumaroyl hexoside, caffeoylshikimic acid.<br><u>Isolation and structure elucidation</u>: monotropein-10-*trans*-*p*-coumarate	HPLC-DAD-ESI-MS^n<br>Spectrophotometric methods<br>isolation; CCC, HR-MS, ^1H and ^{13}C NMR	Methanol-formic acid (99:1, *v/v*) (different colours of the fruits, and time and place of collection)<br>Content in mg/g dw of the extract:<br>TPC (Folin–Ciocalteu assay): 160.9-284.4 mg GAE/g dw;<br>total flavonoids ($AlCl_3$ colorimetric assay): 77.3-174.6 mg CAE/g dw;<br>total anthocyanins (pH-differential method): 2.2-20.4 mg CGE/g dw;<br>total proanthocyanidins (DMAC assay): 25.3-68.1 mg CAE/g dw.<br><br>Content of individual compounds in mg/100 g fw of the fruits:<br><u>Anthocyanins</u> (HPLC-DAD): delphinidin-3-galactoside (1.4-3.9 mg/100 g fw), cyanidin-3-galactoside (4.0-9.5 mg/100 g fw), delphinidin-3-arabinoside (2.8-13.6 mg/100 g fw), cyanidin-3-galactoside (9.4-35.7 mg/100 g fw), total anthocyanins (17.7-60.3 mg/100 g fw);<br><u>Hydroxycinnamic acids</u> (HPLC-DAD): 5-caffeoylquinic acid (2.5-9.0 mg/100 g fw), 3-caffeoylquinic acid (1.3-24.9 mg/100 g fw), caffeoylshikimic acid (2.0-4.2 mg/100 g fw), total hydroxycinnamic acids (3.7-33.9 mg/100 g fw);<br><u>Flavonols</u> (HPLC-DAD): myricetin hexoside (0.3-4.7 mg/100 g fw), myricetin rhamnoside (0.6-1.5 mg/100 g fw), quercetin galactoside (8.0-18.9 mg/100 g fw), quercetin glucuronide (0.3-5.9 mg/100 g fw), quercetin pentoside (2.4-7.2 mg/100 g fw), quercetin arabinoside (1.8-22.5 mg/100 g fw), quercetin rhamnoside (1.7-15.4 mg/100 g fw), quercetin (0.4-8.2 mg/100 g fw), total flavonols (14.6-71.0 mg/100 g fw);<br><u>Coumaroyl iridoids</u> (HPLC-DAD): *p*-coumaroyl monotropein (0.7-54.8 mg/100 g fw), monotropein 10-*trans*-*p*-coumarate	[2,18,19]

	(vaccinoside), 6*α*-hydroxy-dihydromonotropein-10-*trans*-cinnamate, monotropein-10-*trans*-cinnamate.		(vaccinoside; 6.6-395.4 mg/100 g fw), 6*α*-hydroxy-dihydromonotropein-10-*trans*-cinnamate (9.1-46.4 mg/100 g fw), monotropein-10-*trans*-cinnamate (7.3-72.4 mg/100 g fw), total iridoids (70.8-484.2 mg/100 g fw)	
	Pink fruits:<br>Simple phenolic acids: protocatechuic acid hexoside, sinapoyl hexoside;<br>Caffeoylquinic acid isomers: 5-caffeoylquinic acid, 3-caffeoylquinic acid;<br>Anthocyanins: cyanidin-3-*O*-galactoside, cyanidin-3-*O*-arabinoside;<br>Flavonoids: myricetin hexoside, quercetin 3-*O*-galactoside, quercetin pentoside, quercetin 3-*O*-arabinoside, quercetin 3-*O*-rhamnoside, isorhamnetin hexoside, kaempferol pentoside, kaempferol 3-*O*-glucoside, isorhamnetin rhamnoside, kaempferol rhamnoside, quercetin;<br>Iridoids: caffeoyl monotropein, coumaroyl monotropein isomer, monotropein 10-*trans-p*-coumarate, 6*α*-hydroxy-dihydromonotropein-10-*trans*-cinnamate, monotropein-10-*trans*-cinnamate, dihydromonotropein cinnamate;<br>Other compounds: coumaroyl hexoside, coumaroylquinic acid isomer 1, 5-*O*-caffeoylshikimic acid, coumaroylquinic acid isomer 2<br><br>White fruits:<br>Simple phenolic acids: protocatechuic acid hexoside, sinapoyl hexoside;<br>Caffeoylquinic acid isomers: 5-caffeoylquinic acid, 3-caffeoylquinic acid;<br>Anthocyanins: -;<br>Flavonoids: quercetin 3-*O*-galactoside, quercetin 3 *O*-glucuronide, quercetin 3-*O*-glucoside, myricetin glucuronide, quercetin pentoside, quercetin 3-*O*-arabinoside, quercetin 3-*O*-rhamnoside, kaempferol	UHPLC-DAD-ESI-MS<br>HPLC-DAD	Methanol-formic acid (99:1, *v/v*)<br>Content in mg/g dw of the non-digested and after in vitro oral, gastric, and intestinal digestion extract:<br>Anthocyanins (HPLC-DAD): cyanidin-3-*O*-galactoside (pink fruits; non-digested: 5.9 mg/g dw; other: 2.2-2.2 mg/g dw; white fruits; non-digested: - mg/g dw; other: - mg/g dw), cyanidin-3-*O*-arabinoside (pink fruits; non-digested: 19.2 mg/g dw; other: 11.5-11.6 mg/g dw; white fruits; non-digested: - mg/g dw; other: - mg/g dw), total anthocyanins (pink fruits; non-digested: 25.1 mg/g dw; other: 13.7-13.8 mg/g dw; white fruits; non-digested: - mg/g dw; other: - mg/g dw);<br>Hydroxycinnamic acids (HPLC-DAD): 5-*O*-caffeoylquinic acid (pink fruits; non-digested: 1.3 mg/g dw; other: 1.2-1.3 mg/g dw; white fruits; non-digested: 1.4 mg/g dw; other: - mg/g dw), 3-*O*-caffeoylquinic acid (pink fruits; non-digested: 25.0 mg/g dw; other: 11.2-24.1 mg/g dw; white fruits; non-digested: 2.3 mg/g dw; other: 1.1-1.8 mg/g dw), 5-*O*-caffeoylshikimic acid (pink fruits; non-digested: 5.6 mg/g dw; other: 4.0-5.5 mg/g dw; white fruits; non-digested: 6.5 mg/g dw; other: 1.4-6.5 mg/g dw), total hydroxycinnamic acids (pink fruits; non-digested: 31.9 mg/g dw; other: 16.3-30.9 mg/g dw; white fruits; non-digested: 10.2 mg/g dw; other: 2.5-8.2 mg/g dw);<br>Flavonols (HPLC-DAD): myricetin hexoside (pink fruits; non-digested: 1.3 mg/g dw; other: 0.4-1.6 mg/g dw; white fruits; non-digested: - mg/g dw; other: - mg/g dw), quercetin 3-*O*-galactoside (pink fruits; non-digested: 32.3 mg/g dw; other: 18.6-35.6 mg/g dw; white fruits; non-digested: 1.1 mg/g dw; other: 4.1-6.6 mg/g dw), quercetin 3-*O*-glucuronide (pink fruits; non-digested: - mg/g dw; other: - mg/g dw; white fruits; non-digested: - mg/g dw; other: - mg/g dw), quercetin 3-*O*-glucoside (pink fruits; non-digested: 7.6 mg/g dw; other: 4.0-7.8 mg/g dw; white fruits; non-digested: 5.1 mg/g dw; other: 0.7-8.4 mg/g dw), quercetin 3-*O*-arabinoside (pink fruits; non-digested: 17.5 mg/g dw; other: 10.5-20.4 mg/g dw; white fruits; non-digested: 6.4 mg/g dw; other: 0.9-6.4 mg/g dw), quercetin 3-*O*-rhamnoside (pink fruits; non-digested: 11.5 mg/g dw; other: 6.7-11.8 mg/g dw; white fruits;	[20]

	pentoside, kaempferol rhamnoside, quercetin;<br><u>Iridoids</u>: caffeoyl monotropein, coumaroyl monotropein isomer, monotropein 10-*trans-p*-coumarate, 6*α*-hydroxy-dihydromonotropein-10-*trans*-cinnamate, monotropein-10-*trans*-cinnamate, dihydromonotropein cinnamate;<br><u>Other compounds</u>: coumaroyl hexoside, coumaroylquinic acid isomer 1, 5-*O*-caffeoylshikimic acid, coumaroylquinic acid isomer 2		non-digested: 4.5 mg/g dw; other: 0.4-4.5 mg/g dw), quercetin (pink fruits; non-digested: 6.9 mg/g dw; other: 2.9-9.4 mg/g dw; white fruits; non-digested: 1.5 mg/g dw; other: 0.2-1.0 mg/g dw), total flavonols (pink fruits; non-digested: 77.1 mg/g dw; other: 43.1-86.6 mg/g dw; white fruits; non-digested: 18.6 mg/g dw; other: 8.2-18.6 mg/g dw);<br><u>Monotropein iridoids</u> (HPLC-DAD): coumaroyl monotropein isomer (pink fruits; non-digested: 38.6 mg/g dw; other: 25.2-38.9 mg/g dw; white fruits; non-digested: - mg/g dw; other: - mg/g dw), monotropein 10-*trans-p*-coumarate (pink fruits; non-digested: 215 mg/g dw; other: 143-241 mg/g dw; white fruits; non-digested: 28.5 mg/g dw; other: 8.6-18.6 mg/g dw), 6*α*-hydroxy-dihydromonotropein-10-*trans*-cinnamate (pink fruits; non-digested: 15.6 mg/g dw; other: 13.0-19.4 mg/g dw; white fruits; non-digested: 7.1 mg/g dw; other: 6.2-12.5 mg/g dw), monotropein-10-*trans*-cinnamate (pink fruits; non-digested: 3.0 mg/g dw; other: 2.1-3.2 mg/g dw; white fruits; non-digested: 48.7 mg/g dw; other: 30.8-39.8 mg/g dw), dihydromonotropein cinnamate (pink fruits; non-digested: - mg/g dw; other: - mg/g dw; white fruits; non-digested: 4.3 mg/g dw; other: 3.0-3.5 mg/g dw), total monotropein iridoids (pink fruits; non-digested: 272 mg/g dw; other: 183-309 mg/g dw; white fruits; non-digested: 88.6 mg/g dw; other: 49.0-70.7 mg/g dw)	
	<u>Anthocyanins</u>: delphinidin-3-galactoside, cyanidin-3-galactoside, delphinidin-arabinoside, cyanidin-arabinoside;<br><u>Flavonoids</u>: myricetin rhamnoside, quercetin hexoside, quercetin glucuronide, quercetin pentoside, quercetin hexoside, quercetin rhamnoside;<br><u>Iridoids</u>: coumaroyl-iridoid;<br><u>Others</u>: caffeoylglucaric acid	HPLC-DAD<br>Spectrophotometric method	Methanol-water (75:25, *v/v*) (different colours of the fruits, and time and place of collection)<br>Content in mg/g fw of the fruits:<br>TPC (Folin–Ciocalteu assay): 2.28-6.06 mg GAE/g fw;<br>total anthocyanins (HPLC-DAD): ~0.00-0.51 mg CGE/g fw;<br>total flavonols (HPLC-DAD): 0.21-0.41 mg QE/g fw;<br>total iridoids (HPLC-DAD): 0.12-0.22 mg/g fw;<br>ascorbic acid (HPLC-DAD): 10.29-14.26 mg/g fw	[22]
	–	HPLC-DAD<br>HPLC-FL	Methanol-formic acid (99:1, *v/v*)<br>Content in μg or mg/100 g fw of the fruits:<br>Carotenoids (HPLC-DAD):<br>lutein (pink fruits: 19.54 μg/100 g fw; white fruits: 9.15 μg/100 g fw); zeaxanthin (pink fruits: 1.57 μg/100 g fw; white fruits: 2.07 μg/100 g fw); β-cryptoxanthin (pink fruits: 0.13 μg/100 g fw; white fruits: 1.24 μg/100 g fw); lycopene (pink fruits: 0.41 μg/100 g fw; white fruits: 0.30 μg/100 g fw); *α*-carotene (pink fruits: 0.27 μg/100	[21]

			g fw; white fruits: 0.19 μg/100 g fw); β-carotene (pink fruits: 15.19 μg/100 g fw; white fruits: 3.65 μg/100 g fw); total carotenoids (pink fruits: 37.11 μg/100 g fw; white fruits: 16.62 μg/100 g fw); Tocochromanols (HPLC-FL): α-tocopherol (pink fruits: 0.28 mg/100 g fw; white fruits: 0.148 mg/100 g fw); γ-tocopherol (pink fruits: 0.038 mg/100 g fw; white fruits: 0.021 mg/100 g fw); δ-tocopherol (pink fruits: 0.003 mg/100 g fw; white fruits: 0.004 mg/100 g fw); α-tocotrienol (pink fruits: nd; white fruits: nd); β-tocotrienol (pink fruits: nd; white fruits: 0.006 mg/100 g fw); γ-tocotrienol (pink fruits: 0.114 mg/100 g fw; white fruits: 0.11 mg/100 g fw); δ-tocotrienol (pink fruits: 0.012 mg/100 g fw; white fruits: 0.01 mg/100 g fw); total tocochromanols (pink fruits: 0.447 mg/100 g fw; white fruits: 0.299 mg/100 g fw); ascorbic acid (HPLC-DAD): pink fruits: 24.88 mg/100 g fw; white fruits: 3.33 mg/100 g fw	
G. procumbens L.	Salicylates: gaultherin, physanguloside, 2-*O*-β-D-glucopyranosyl gaultherin; Simple phenolic acids: *p*-hydroxybenzoic acid, protocatechuic acid, derivatives of vanillic, sinapinic, protocatechuic, and caffeic acids; Caffeoylquinic acid isomers: chlorogenic acid, neochlorogenic acid; Quinic acid derivatives: 3-*O*-*p*-coumaroylquinic acid derivative, caffeoylquinic acid derivative; Flavonoids: (aglycones) quercetin, kaempferol; (glycosides) hyperoside, isoquercitrin, miquelianina, quercetin hexosyl-rhamnoside, quercitrin, kaempferol 3-*O*-glucuronide; Catechins: (+)-catechin, (–)-epicatechin; Procyanidins: A-type dimers, B-type dimers, A-type trimers, B-type trimers, procyanidin B2, cinnamtannin B1, procyanidin C1	UHPLC-PDA-ESI-MS/MS HPLC-PDA Spectrophotometric methods isolation; HR-ESI-MS, ^{1}H NMR; ^{13}C NMR; 2D NMR; identification of aglycone (GC-MS) and sugars (acid hydrolysis, HPLC-PDA)	Content in mg/g dw of the extract: Methanol-water (75:25, *v*/*v*): TPC (Folin–Ciocalteu assay): 79.71 mg GAE/g dw; total proanthocyanidins (*n*-BuOH/HCl method): 62.39 mg CYE/g dw; total salicylates (HPLC-PDA): 83.66 mg/g dw; total simple phenolic acids (HPLC-PDA): 2.25 mg/g dw; total flavonoids (HPLC-PDA): 1.17 mg/g dw; total proanthocyanidins (HPLC-PDA): 7.41 mg/g dw; Ethyl acetate extract: TPC (Folin–Ciocalteu assay): 17.26 mg GAE/g dw; total proanthocyanidins (*n*-BuOH/HCl method): 0.58 mg CYE/g dw; total salicylates (HPLC-PDA): 109.28 mg/g dw; total simple phenolic acids (HPLC-PDA): 6.03 mg/g dw; total flavonoids (HPLC-PDA): 0.065 mg/g dw; total proanthocyanidins (HPLC-PDA): –; *n*-Butanol extract: TPC (Folin–Ciocalteu assay): 23.46 mg GAE/g dw; total proanthocyanidins (*n*-BuOH/HCl method): 2.92 mg CYE/g dw; total salicylates (HPLC-PDA): 63.77 mg/g dw; total simple phenolic acids (HPLC-PDA): 2.80 mg/g dw; total flavonoids (HPLC-PDA): 0.66 mg/g dw; total proanthocyanidins (HPLC-PDA): –; Acetone extract: TPC (Folin–Ciocalteu assay): 56.05 mg GAE/g dw; total proanthocyanidins (*n*-BuOH/HCl method): 41.26 mg CYE/g dw;	[23–25]

total salicylates (HPLC-PDA): 121.67 mg/g dw; total simple phenolic acids (HPLC-PDA): 3.23 mg/g dw; total flavonoids (HPLC-PDA): 0.74 mg/g dw; total proanthocyanidins (HPLC-PDA): 9.59 mg/g dw;
Water extract:
TPC (Folin–Ciocalteu assay): 46.62 mg GAE/g dw; total proanthocyanidins (*n*-BuOH/HCl method): 30.01 mg CYE/g dw; total salicylates (HPLC-PDA): 31.09 mg/g dw; total simple phenolic acids (HPLC-PDA): 3.60 mg/g dw; total flavonoids (HPLC-PDA): 0.33 mg/g dw; total proanthocyanidins (HPLC-PDA): 1.24 mg/g dw;

Content of individual compounds in mg/g dw of the extract:
Methanol-water (75:25, *v*/*v*):
gaultherin (48.89 mg/g dw); physanguloside (12.45 mg/g dw); 2-*O*-β-D-glucopyranosyl gaultherin (22.31 mg/g dw); protocatechuic acid (0.16 mg/g dw); *p*-hydroxybenzoic acid (0.08 mg/g dw); chlorogenic acid (0.37 mg/g dw); quercetin (0.14 mg/g dw); miquelianina (0.95 mg/g dw); (–)-epicatechin (2.36 mg/g dw); procyanidin B2 (2.86 mg/g dw), cinnamtannin B1 (0.89 mg/g dw);
Ethyl acetate extract: gaultherin (98.25 mg/g dw); physanguloside (8.58 mg/g dw); 2-*O*-β-D-glucopyranosyl gaultherin (2.45 mg/g dw); protocatechuic acid (0.35 mg/g dw); *p*-hydroxybenzoic acid (0.22 mg/g dw); chlorogenic acid (0.59 mg/g dw); quercetin (0.031 mg/g dw); miquelianina (nd); (–)-epicatechin (nd); procyanidin B2 (nd), cinnamtannin B1 (nd);
n-Butanol extract: gaultherin (29.35 mg/g dw); physanguloside (6.61 mg/g dw); 2-*O*-β-D-glucopyranosyl gaultherin (27.81 mg/g dw); protocatechuic acid (0.43 mg/g dw); *p*-hydroxybenzoic acid (0.07 mg/g dw); chlorogenic acid (0.35 mg/g dw); quercetin (0.52 mg/g dw); miquelianina (nd); (–)-epicatechin (nd); procyanidin B2 (nd), cinnamtannin B1 (nd);
Acetone extract: gaultherin (93.63 mg/g dw); physanguloside (16.09 mg/g dw); 2-*O*-β-D-glucopyranosyl gaultherin (11.95 mg/g dw); protocatechuic acid (0.30 mg/g dw); *p*-hydroxybenzoic acid (0.21 mg/g dw); chlorogenic acid (0.76 mg/g dw); quercetin (0.16 mg/g dw); miquelianina (0.52 mg/g dw); (–)-epicatechin (1.57 mg/g dw); procyanidin B2 (2.28 mg/g dw), cinnamtannin B1 (3.48 mg/g dw);

			Water extract: gaultherin (2.73 mg/g dw); physanguloside (7.77 mg/g dw); 2-*O*-β-D-glucopyranosyl gaultherin (20.59 mg/g dw); protocatechuic acid (0.27 mg/g dw); *p*-hydroxybenzoic acid (0.09 mg/g dw); chlorogenic acid (0.40 mg/g dw); quercetin (0.021 mg/g dw); miquelianina (0.29 mg/g dw); (–)-epicatechin (0.22 mg/g dw); procyanidin B2 (0.34 mg/g dw), cinnamtannin B1 (0.68 mg/g dw)	
	Essential oil components (relative contents expressed as a % of the total oil components): pent-4-enal (0.01%), hexenal (0.03%), furfural (0.03%), 4-methylhex-5-en-2-ol (0.02), heptan-2-ol (0.07%), benzaldehyde (0.01%), 2-pentylfuran (0.01%), limonene (0.01%), benzyl alcohol (0.55%), octan-1-ol (0.01%), heptanoic acid (0.37%), nonanal (0.01%), linalool (0.01%), naphthalene (0.01%), octanoic acid (0.08%), isopinocarveol (0.01%), methyl salicylate (97.47%), dec-3-en-1-ol (0.01%), nonanoic acid (0.01%), *p*-cymen-7-ol (0.06), eugenol (0.02%), 2,6-dihydroxybenzoic acid methyl ester (0.02%), isoeugenol (0.01%), 3-hydroxyphenylacetic acid (0.39%)	GC-FID-MS	Essential oil (hydrodistillation)	[23,26]
	Ethanolic extract<br>Salicylates: gaultherin	GC-MS<br>LC-PDA-ESI-MS<br>FAB⁺ MS	Methanolic extract after NaOH hydrolysis and SPE<br>Content in μg/g fw of the fruits:<br>total salicylic acid (stable-isotope dilution GC-MS): 1.491 μg/g fw	[27]
G. pumila (L.f.) D.J.Middleton	–	Spectrophotometric method	Ethanolic extract acidified (pH = 4.0)<br>Content in mg/100 g fw of the fruits:<br>total anthocyanins (pH-differential method): pepper white fruits (626.2 mg CGE/100 g fw); round white fruits (~800-1200 mg CGE/100 g fw); pink fruits (3854 mg CGE/100 g fw); red fruits (5942 mg CGE/100 g fw);<br><br>Aqueous acidic extract with ethanol precipitation<br>Pectin content in % fw of the fruits: pepper white fruits (~1.2%); round white fruits (~2.8-3.0%); pink fruits (~1.4%); red fruits (~12%)	[28]
	<u>Anthocyanins</u>: delphinidine-3-*O*-arabinoside, delphinidine-3-*O*-galactoside, cyanidin-3-*O*-galactoside, cyanidin-3-*O*-arabinoside, peonidin-3-*O*-glucoside;	UHPLC-DAD-Orbitrap-MS<br>Spectrophotometric methods	Methanol-formic acid (98:2, *v*/*v*)<br>Content in mg/g dw of the extract:<br>TPC (Folin–Ciocalteu assay): 189.2 mg GAE/g;<br>total flavonoids ($AlCl_3$ colorimetric assay): 51.8 mg QE/g;<br>total anthocyanins (pH-differential method): 47.3 mg CGE/g	[29]

	Flavonoids: myricetin *O*-hexoside, myricetin *O*-rhamnoside, quercetin 3-*O*-riboside, quercetin 3-*O*-arabinoside, quercetin 3-*O*-xyloside, quercitrin, quercetin; luteolin 7-glucuronide, eupatorine, pongapin;<br>Phenolic acids: vaccihein A, coumaric acid glucoside, 5-*O*-caffeoylshikimic acid, caffeic acid derivative, caffeoyl-feruloyl-tartaric acid;<br>Caffeoylquinic acid isomers: chlorogenic acid, caffeoylquinic acid derivative;<br>Proanthocyanidins: procyanidin B-type dimer;<br>Iridoids and secoiridoids: unedide, *p*-coumaroyl monotropein isomer, nuzhenal B;<br>Diterpenes and sesquiterpenes: enmenol, scupolin I, dictamnoside M;<br>Fatty acids and lipid derivatives: pinellic acid, octadecanedioic acid, 2-hydroxyeicosanoic acid;<br>Others: uralenneoside, methyl gallate derivative			
G. shallon Pursh	Catechins: (+)-catechin, (–)-epicatechin	RP-TLC (detection: vanillin-sulfuric acid reagent)<br>RP-HPLC	Ethyl acetate fraction of the methanolic extract	[30]
	–	Spectrophotometric methods	Acetonitrile-0.2% formic acid (different harvest times: 2012, 2013, 2014)<br>Content in mg/100 g fw of the fruits:<br>TPC (Folin–Ciocalteu assay): 658-968 mg GAE/100 g fw;<br>total anthocyanins (pH-differential method): 134-219 mg CGE/100 g fw	[31]
	Acetonitrile-0.2% formic acid<br>Anthocyanins: delphinidin-pentose-hexose, cyanidin-pentose-hexose, cyanidin-dipentose;<br>Caffeoylquinic acid isomers: chlorogenic acid;<br>Proanthocyanidins: procyanidin B-type trimers, procyanidin B-type tetramers;<br>Flavonoids: myricetin hexoside, myricetin pentoside, myricetin rhamnoside, myricetin	LC-PDA-ESI-MS/MS<br>Spectrophotometric methods	Acetonitrile-0.2% formic acid<br>Content in mg/100 g fw of the fruits:<br>TPC (Folin–Ciocalteu assay): 975 mg GAE/100 g fw;<br>total anthocyanins (pH-differential method): 256 mg CGE/100 g fw;<br><br>Content in % of total anthocyanins (LC-MS):<br>cyanidin-pentoside (8.4% of total anthocyanins), delphinidin-pentoside (24.4%), cyanidin-hexoside (7.6%), delphinidin-	[32]

	glucuronide, quercetin hexoside, quercetin pentoside, quercetin rhamnoside, quercetin glucuronide.<br><br>Wine prepared from fruits<br><u>Anthocyanins</u>: delphinidin-pentose-hexose, delphinidin-hexose, cyanidin-pentose-hexose, delphinidin-dipentose, cyanidin-dipentose, pyroanthocyanin (vitisin A-type derivative);<br><u>Phenolic acids</u>: gallic acid, dihydroxybenzoic acid;<br><u>Proanthocyanidins</u>: procyanidin B-type dimers, galloylated procyanidin, procyanidin B-type trimer, procyanidin B-type tetramer;<br><u>Caffeoylquinic acid isomers</u>: chlorogenic acid;<br><u>Catechins</u>: (–)-epicatechin;<br><u>Flavonoids</u>: myricetin pentoside, myricetin rhamnoside, myricetin glucuronide, myricetin, quercetin pentoside, quercetin rhamnoside, quercetin glucuronide, quercetin;<br><u>Others</u>: phloroglucinaldehyde		hexoside (23.9%), cyanidin-dipentoside (5.8%), delphinidin-dipentoside (5.6%), cyanidin-pentose-hexose (sambubioside-like; 7.1%), delphinidin-pentose-hexose (sambubioside-like; 17.2%)	
	Methanol-water (80:20, *v*/*v*)<br><u>Anthocyanins</u>: cyanidin-3-*O*-lathyroside, cyanidin-3-*O*-arabinoside, cyanidin-3-*O*-galactoside, cyanidin-3-*O*-sambucoside, cyanidin-3-*O*-glucoside, delphinidin-3-*O*-arabinoside, delphinidin-3-*O*-galactoside, delphinidin-3-*O*-glucoside, petunidin-3-*O*-glycoside, malvidin-3-*O*-glycoside, peonidin-3-*O*-glycoside;<br><u>Flavan-3-ols</u>: (+)-catechin, (–)-epicatechin, gallocatechin, epigallocatechin;<br><u>Procyanidins</u>: procyanidin B1, procyanidin B2, procyanidin B4, procyanidin B3, procyanidin A2;	UHPLC-MS/MS<br>Spectrophotometric methods	Methanolic extract (different stages of fruit development: immature and mature berries)<br>Content in mg/g dw of the fruits:<br>total proanthocyanidins (*n*-BuOH/HCl assay): 52.8-280.7 mg/g dw;<br>total anthocyanins (pH-differential method): ~110-~1600 mg CGE/100 g dw.<br><br>Methanol-water (80:20, *v*/*v*) (different stages of fruit development: immature and mature berries)<br>Content of individual compounds (UHPLC-MS/MS) in mg/100 g dw of the fruits:<br><u>Anthocyanin</u>s: cyanidin-3-*O*-lathyroside (18-57 mg/100 g dw), cyanidin-3-*O*-arabinoside (39-83 mg/100 g dw), cyanidin-3-*O*-galactoside (37-130 mg/100 g dw), cyanidin-3-*O*-sambucoside (4.5-9.9 mg/100 g dw), cyanidin-3-*O*-glucoside (9-12 mg/100 g dw),	[33]

	Caffeoylquinic acid isomers: chlorogenic acid, neochlorogenic acid, cryptochlorogenic acid;<br>Flavonoids: quercetin 3-*O*-rhamnoside, quercetin 3-*O*-galactoside, quercetin 3-*O*-glucuronide, quercetin 3-*O*-glucoside, quercetin 3-*O*-sulfate,<br>myricetin, myricitrin, apigenin, kaempferol 3-*O*-rutinoside, rutin, luteolin 7-*O*-glucoside, naringenin, naringenin 7-*O*-glucoside, isorhamnetin 3-*O*-rutinoside		delphinidin-3-*O*-arabinoside (400-700 mg/100 g dw), delphinidin-3-*O*-galactoside (770-2600 mg/100 g dw), delphinidin-3-*O*-glucoside (130-280 mg/100 g dw), petunidin-3-*O*-glycoside (0.3-3.6 mg/100 g dw), malvidin-3-*O*-glycoside (nd), peonidin-3-*O*-glycoside (nd);<br>Flavan-3-ols: (+)-catechin (150-580 mg/100 g dw), (–)-epicatechin (27-84 mg/100 g dw), gallocatechin (21-49 mg/100 g dw), epigallocatechin (2.3-5.5 mg/100 g dw);<br>Proanthocyanidins: procyanidin B1 (29-76 mg/100 g dw), procyanidin B2 + B4 (14-55 mg/100 g dw), procyanidin B3 (8-41 mg/100 g dw), procyanidin A2 (2.1-10 mg/100 g dw);<br>Caffeoylquinic acid isomers: chlorogenic acid (4.2-6.4 mg/100 g dw), neochlorogenic acid (23-42 mg/100 g dw), cryptochlorogenic acid (0.04-0.2 mg/100 g dw);<br>Flavonoids: quercetin 3-*O*-rhamnoside (5.8-17 mg/100 g dw), quercetin 3-*O*-galactoside (19-35 mg/100 g dw), quercetin 3-*O*-glucuronide (24-46 mg/100 g dw), quercetin 3-*O*-glucoside (nd), quercetin 3-*O*-sulfate (2-8 mg/100 g dw), myricetin (0.1-4.1 mg/100 g dw), myricitrin (6-17 mg/100 g dw), apigenin (0.03-0.06 mg/100 g dw), kaempferol 3-*O*-rutinoside (nd), rutin (nd), luteolin 7-*O*-glucoside (0.32-0.6 mg/100 g dw), naringenin (0.1-0.37 mg/100 g dw), naringenin 7-*O*-glucoside (3-36 mg/100 g dw), isorhamnetin 3-*O*-rutinoside (nd)	
	–	Spectrophotometric method	Ethanolic extract<br>Content in mol/L dw of the extract:<br>TPC (Folin–Ciocalteu assay): 13.62 mol GAE/L dw	[34]
G. tenuifolia (Phil.) Sleumer	Anthocyanins: cyanidin-3-*O*-β-galactoside, cyanidin-3-*O*-β-arabinoside;<br>Catechins: (+)-catechin;<br>Proanthocyanidins: B-type proanthocyanidin dimer 1 ((epi)C-(epi)C-gallate), B-type procyanidin dimer 2 ((epi)C-(epi)C), B-type procyanidin dimer 3 ((epi)C-(epi)C), B-type procyanidin dimer 4 ((epi)C-(epi)C), B-type procyanidin dimer 5 ((epi)C-(epi)C), B-type procyanidin dimer 6 ((epi)C-(epi)C), B-type procyanidin dimer 7 ((epi)C-(epi)C), B-type procyanidin dimer 8 ((epi)C-(epi)C), B-type procyanidin trimer 1 ((epi)C-(epi)C-(epi)C), B-type procyanidin trimer 2 ((epi)C-(epi)C-	HPLC-DAD-ESI-MS^n<br>HS-SPME-GC-MS	Methanol-1% formic acid (different collection years: 2018, 2019)<br>Content of individual compounds (HPLC-DAD) in mg/100 g fw of the fruits:<br>Anthocyanins: cyanidin-3-galactoside (3.12-5.46 mg/100 g fw), cyanidin-3-arabinoside (35.82-48.09 mg/100 g fw), total anthocyanins (41.28-51.21 mg/100 g fw);<br>Hydroxycinnamic acids: 3-*O*-caffeoylquinic acid (7.56-9.00 mg/100 g fw), 5-*O*-caffeoylshikimic acid (20.12-24.94 mg/100 g fw), total hydroxycinnamic acids (29.12-32.50 mg/100 g fw);<br>Catechins and proanthocyanidins: (epi)C-(epi)C dimer 4 (5.47-17.79 mg/100 g fw), (+)-catechin (7.75-24.46 mg/100 g fw), (epi)C-(epi)C dimer 5 (4.52-28.09 mg/100 g fw), (epi)C-A-(epi)C-(epi)C trimer 2 (7.44-25.99 mg/100 g fw), (epi)C-(epi)C-(epi)C trimer 4	[35]

	(epi)C), B-type procyanidin trimer 3 ((epi)C-(epi)C-(epi)C), B-type procyanidin trimer 4 ((epi)C-(epi)C-(epi)C), B-type procyanidin trimer 5 ((epi)C-(epi)C-(epi)C), B-type procyanidin trimer 6 ((epi)C-(epi)C-(epi)C), B-type procyanidin trimer 7 ((epi)C-(epi)C-(epi)C), A-type procyanidin trimer 1 ((epi)C-A-(epi)C-(epi)C), A-type procyanidin trimer 2 ((epi)C-A-(epi)C-(epi)C), A-type procyanidin trimer 3 ((epi)C-(epi)C-A-(epi)C), A-type prodelphinidin dimer ((epi)GC-(epi)C);<br>Flavonoids: irigenin hexoside, quercetin 3-O-β-glucuronide, quercetin 3-*O*-β-rutinoside, quercetin 3-*O*-β-glucoside, quercetin 3-*O*-β-arabinoside, quercetin 3-*O*-β-rhamnoside, quercetin acetylhexoside 1, quercetin acetylhexoside 2, quercetin, kaempferol 3-*O*-β-glucoside, kaempferol glucuronide, kaempferol rutinoside, kaempferol pentoside;<br>Iridoids: swertiamarin, methoxygeniposidic acid, 6α-hydroxy-dihydromonotropein-10-*trans*-cinnamate, monotropein-10-*trans*-cinnamate, dihydromonotropein cinnamate;<br>Caffeoyl acids: 3-*O*-caffeoylquinic acid, 5-*O*-caffeoylshikimic acid.<br><br>Essential oil components (GC-MS): dimethyl sulphide, 3-methylbutanal, 2-methylbutyraldehyde, hexanal, trans-2-hexenal, trans-2-hexenol, 1-hexanol, 2-heptanone, 2-heptanol, benzaldehyde, 6-methyl-5-hepten-2-one, 2,4-heptadienal, p-cymene, linalool oxide, methyl benzoate, linalool, 2-phenylethanol, ethyl benzoate, α-terpineol, methyl salicylate, anethole, γ-dodecalactone		(9.69-35.33 mg/100 g fw), total catechins and proanthocyanidins (34.87-131.66 mg/100 g fw);<br>Flavonoids: irigenin hexoside (0.74-1.08 mg/100 g fw), quercetin 3-*O*-β-glucuronide + quercetin 3-*O*-β-rutinoside (59.72-76.10 mg/100 g fw), quercetin 3-*O*-β-glucoside (0.52-0.76 mg/100 g fw), quercetin 3-*O*-β-arabinoside (6.63-7.03 mg/100 g fw), kaempferol 3-*O*-β-glucoside (0.54-2.18 mg/100 g fw), quercetin 3-*O*-β-rhamnoside (8.20-8.29 mg/100 g fw), quercetin (0.99-1.27 mg/100 g fw), total flavonoids (80.33-93.72 mg/100 g fw);<br>Iridoids: swertiamarin (5.47-8.18 mg/100 g fw), methoxygeniposidic acid (3.82-8.35 mg/100 g fw), 6α-hydroxy-dihydromonotropein-10-*trans*-cinnamate (0.58-8.73 mg/100 g fw), monotropein-10-*trans*-cinnamate (1.73-2.54 mg/100 g fw), dihydromonotropein cinnamate (1.90-2.57 mg/100 g fw), total iridoids (21.65-22.22 mg/100 g fw)	
G. trichophylla Royle	–	Spectrophotometric methods<br>Flame photometry	Methanol-water (80:20, *v/v*) (different sampling sites)<br>Content in mg/100 g fw of the fruits:<br>TPC (Folin–Ciocalteu assay): ~3.28-3.71 mg GAE/100 g fw;<br>total tannins (Folin-Dennis method): ~2.15-2.62 mg TAE/100 g fw;	[36,37]

			total flavonoids ($AlCl_3$ colorimetric assay): ~1.42-1.75 mg QE/100 g fw;<br>total flavonols ($AlCl_3$-sodium acetate colorimetric assay): ~0.86-1.03 mg CAE/100 g fw.<br><br>Content of nutrients:<br>total carbohydrates (spectrophotometry): ~0.78-1.02 mg/100 g fw;<br>sodium (flame photometry): ~1.35-1.70 mg/100 g fw;<br>potassium (flame photometry): ~1.85-2.17 mg/100 g fw	
	Colour-precipitation reactions: glycosides, flavonoids, saponins, tannins, phenolics, terpenoids, alkaloids	Spectrophotometric methods<br>Atomic Absorption Spectrophotometry (AAS)	*n*-hexane, chloroform, ethyl acetate, *n*-butanol, methanolic, water extracts<br>Content in mg/g or μg/g dw of the extract:<br>TPC (Folin–Ciocalteu assay): *n*-hexane (14.17 mg GAE/g dw), chloroform (~10 mg GAE/g dw), ethyl acetate (~11 mg GAE/g dw), *n*-butanol (~5 mg GAE/g dw), methanolic (13.54 mg GAE/g dw), water (3.27 mg GAE/g dw);<br>total flavonoids ($AlCl_3$ colorimetric assay): *n*-hexane (26.8 mg QE/g dw), chloroform (~24 mg QE/g dw), ethyl acetate (~21 mg QE/g dw), *n*-butanol (~9 mg QE/g dw), methanolic (26.9 mg QE/g dw), water (8.08 mg QE/g dw);<br>Elemental analysis (AAS): sodium (3.2418 μg/g dw), magnesium (1.9301 μg/g dw), calcium (1.8350 μg/g dw), potassium (0.0107 μg/g dw), copper (~0.65 μg/g dw), chromium (~0.20 μg/g dw), nickel (~0.05 μg/g dw), manganese (~0.04 μg/g dw), cobalt (~0.10 μg/g dw), lead (~0.40 μg/g dw), cadmium (~0.20 μg/g dw), iron (~1.80 μg/g dw); all trace/heavy metals were below WHO permissible limits	[38]
	Colour-precipitation reactions: flavonoids, saponins, tannins, phenolics, terpenoids, alkaloids	Spectrophotometric methods<br>GC-MS	Methanolic extract<br>Content in mg/g dw of the extract:<br>TPC (Folin–Ciocalteu assay): 29.76-138.88 mg GAE/g dw;<br>total flavonoids ($AlCl_3$ colorimetric assay): 13.34-53.96 mg QE/g dw.<br><br>Content of individual compounds (GC-MS): 2,3-dihydro-3,5-dihydroxy-6-methyl-4H-pyran-4-one (15.64%), hexadecanoic acid (palmitic acid, 15.19%), Octadec-9-enoic acid (oleic acid, 13.78%), 9,12-Octadecadienoic acid (linoleic acid, 11.04%), 5-hydroxymethylfurfural (7.81%), 4H-pyran-4-one, 3-hydroxy-2-methyl (7.17%), 4-oxopentanoic acid (4.07%), 4-methyl-6-	[39]

			(tetrahydropyran-2-yloxy)hex-4-enal (3.06%), 2-decenal (2.82%), octadecanoic acid (stearic acid, 2.50%)	
–		Spectrophotometric methods<br>Atomic Absorption Spectrophotometry (AAS)	Methanolic extract<br>Content in g/100 g dw of the fruits:<br>TPC (Folin–Ciocalteu assay): 1.75 g GAE/100 g dw;<br>total tannins (Folin-Dennis method): 2.62 mg TAE/g dw;<br>total flavonoids ($AlCl_3$ colorimetric assay): 4.13 g QE/100 g dw;<br>total flavonols ($AlCl_3$-sodium acetate colorimetric assay): 0.012 mg CAE/100 g dw;<br>Elemental analysis (AAS): magnesium (4.115 mg/100 g dw), calcium (0.2191 mg/100 g dw), iron (0.089 mg/100 g dw), copper 0.97 mg/100 g dw), manganese (0.0118 mg/100 g dw), sodium (0.3186 mg/100 g dw), zinc (0.0128 mg/100 g dw)	[7]

AAE: ascorbic acid equivalents; CAE: (+)-catechin equivalent; CGE: cyanidin-3-glucoside equivalents; CYE: cyanidin chloride equivalents; QE: quercetin equivalents; TAE: tannic acid equivalents; TPC: total phenolic content expressed in gallic acid equivalents (mg GAE/g); DMAC: 4-dimethylaminocinnamaldehyde assay; dw: dry weight; fw: fresh weight; nd – not detected.

References

1. Ruiz, A.; Hermosín-Gutiérrez, I.; Vergara, C.; von Baer, D.; Zapata, M.; Hitschfeld, A.; Obando, L.; Mardones, C. Anthocyanin profiles in south Patagonian wild berries by HPLC-DAD-ESI-MS/MS. *Food Res. Int.* **2013**, *51*, 706–713, doi:10.1016/j.foodres.2013.01.043.
2. Bouyahya, A.; Omari, N. El; EL Hachlafi, N.; Jemly, M. El; Hakkour, M.; Balahbib, A.; El Menyiy, N.; Bakrim, S.; Naceiri Mrabti, H.; Khouchlaa, A.; et al. Chemical compounds of berry-derived polyphenols and their effects on gut microbiota, inflammation, and cancer. *Molecules* **2022**, *27*, 3286, doi:10.3390/molecules27103286.
3. Ruiz, A.; Bustamante, L.; Vergara, C.; von Baer, D.; Hermosín-Gutiérrez, I.; Obando, L.; Mardones, C. Hydroxycinnamic acids and flavonols in native edible berries of South Patagonia. *Food Chem.* **2015**, *167*, 84–90, doi:10.1016/j.foodchem.2014.06.052.
4. Bravo, K.; Alzate, F.; Osorio, E. Fruits of selected wild and cultivated Andean plants as sources of potential compounds with antioxidant and anti-aging activity. *Ind. Crops Prod.* **2016**, *85*, 341–352, doi:10.1016/j.indcrop.2015.12.074.
5. Plazas González, E.A. Preliminary phytochemical screening, antioxidant, and toxic activity evaluation of six species of colombian Ericaeas. *Rev. Cuba. Plantas Med.* **2015**, *20*, 182–199.
6. Karuppusamy, S.; Muthuraja, G.; Rajasekaran, K.M. Antioxidant activity of selected lesser known edible fruits from Western Ghats of India. *Indian J. Nat. Prod. Resour.* **2011**, *2*, 174–178.
7. Barman, M.; Mishra, P. Comprehensive review on ethnobotanical, nutritional and functional properties of wild edible fruits of North Eastern Himalaya, India. *J. Food Sci. Technol.* **2025**, *62*, 1407–1424, doi:10.1007/s13197-025-06351-7.
8. Espinoza, M.; Gómez, E.; Quispe, S.; Sánchez-González, J.A.; León-Vargas, J. Physicochemical and nutraceutical characterization of sirimbache fruit (*Gaultheria glomerata* (Cav.) Sleumer). *Sci. Agropecu.* **2017**, *8*, 411–417, doi:10.17268/sci.agropecu.2017.04.12.
9. Barragán-Condori, M.; Delgado-Laime, M. del C.; Carrasco-Sauñe, E.; Quispe-Gutiérrez, U.S. Antocianinas y capacidad antioxidante en extractos de frutos secos y congelados de *Gaultheria glomerata* (Cav.) Sleumer. *Inf. tecnológica* **2021**, *32*, 3–12, doi:10.4067/S0718-07642021000500003.
10. Aronés Jara, M.R.; Cárdenas Landeo, E.; Luna Molero, H.R.; Barbarán Vilcatoma, S.M.; Gómez Quispe, M. Tamizaje fitoquimico, contenido de compuestos fenolicos y potencial antioxidante de trece plantas medicinales de los afloramientos rocosos del bosque de pierdras de huaraca en Peru. *Rev. la Soc. Química del Perú* **2022**, *88*, 165-179. doi:10.37761/rsqp.v88i2.388.
11. Huarancca-Huarcaya, E.; Paredes-Quiroz, L.R.; Pilares-Estrada, N.M.; Barragán-Condori, M.; Huamaní-Meléndez, V.J. Cinética da degradação térmica de antocianinas de alaybilí (*Vaccinium floribundum* Kunth) e macha-macha (*Gaultheria glomerata* (Cav.) Sleumer). *Brazilian J. Food Technol.* **2022**, *25*, 1–10, doi:10.1590/1981-6723.10621.
12. Zapata, L.; Heredia, A.; Quinteros, C.; Malleret, A.; Clemente, G.; Cárcel, J. Optimización de la extracción de antocianinas de arándanos. *Cienc. Docencia y Tecnol.* **2014**, *25*, 166–192.
13. Ore-Areche, F.; Flores, D.D.C.; Pacovilca-Alejo, O. V.; Montesinos, C.C.Z.; Carrasco, S.M.; Chirre, E.T.C.; Camayo-Lapa, B.F.; Solano, M.A.Q.; Hinojosa, R.C.A.; De La Cruz-Porta, E.A.; et al. Effect of different concentrations of stem juice of *Oxalis tuberosa* Mol. and juice of the fruit of *Gaultheria glomerata* (Cav.) Sleumer on the antioxidant activity of the heat-treated functional beverage. *Brazilian J. Biol.* **2023**, *83*, 1–7, doi:10.1590/1519-6984.274986.
14. Klavins, L.; Klavins, M. Cuticular wax composition of wild and cultivated northern berries. *Foods* **2020**, *9*, 587, doi:10.3390/foods9050587.
15. Saleh, M.; Glombitza, W. Volatile oil of *Pernettya mucronata* and its antischistosomal activity. *Planta Med.* **1986**, *52*, 557–557, doi:10.1055/s-2007-969372.
16. Díaz Paico, L.Y.; Braúl Porras, E.G. Cuantificación de vitamina C y evaluación de la capacidad antioxidante del fruto de *Pernettya prostrata* (Cav.) DC. (macha macha) frente al radical 2,2-difenil-1-picrilhidrazilo, Universidad Nacional de Trujillo: Trujillo, Peru, 2018.
17. Cahuana Quispe, C. Actividad antioxidante y polifenoles totales del extracto etanólico del fruto de *Pernettya prostrata* "macha-macha", Universidad Nacional "San Luis Gonzaga": Ica, Peru, 2025.
18. Mieres-Castro, D.; Schmeda-Hirschmann, G.; Theoduloz, C.; Gómez-Alonso, S.; Pérez-Navarro, J.; Márquez, K.; Jiménez-Aspee, F. Antioxidant activity and the isolation of polyphenols and new iridoids from Chilean *Gaultheria phillyreifolia* and *G. poeppigii* berries. *Food Chem.* **2019**, *291*, 167–179, doi:10.1016/j.foodchem.2019.04.019.
19. Mieres-Castro, D.; Schmeda-Hirschmann, G.; Theoduloz, C.; Gómez-Alonso, S.; Pérez-Navarro, J.; Márquez, K.; Jiménez-Aspee, F. Chilean "chaura" berries (*Gaultheria phillyreifolia* and *G. poeppigii*): isolation of secondary metabolites and antioxidant activity. *Planta Med.* **2019**, *85*, 1479–1479, doi:10.1055/s-0039-3399871.

20. Mieres-Castro, D.; Theoduloz, C.; Sus, N.; Burgos-Edwards, A.; Schmeda-Hirschmann, G.; Frank, J.; Jiménez-Aspee, F. Iridoids and polyphenols from chilean *Gaultheria* spp. berries decrease the glucose uptake in Caco-2 cells after simulated gastrointestinal digestion. *Food Chem.* **2022**, *369*, 130940, doi:10.1016/j.foodchem.2021.130940.
21. Lauer, L.A.; Mieres-Castro, D.; Schmalle, V.; Frank, J.; Jiménez-Aspee, F. Cytoprotective effects of iridoids from *Gaultheria* spp. berries in gastric and intestinal epithelial cells after in vitro digestion. *Food Biosci.* **2024**, *57*, 103587, doi:10.1016/j.fbio.2024.103587.
22. Oyarzún, P.; Cornejo, P.; Gómez-Alonso, S.; Ruiz, A. Influence of profiles and concentrations of phenolic compounds in the coloration and antioxidant properties of *Gaultheria poeppigii* fruits from Southern Chile. *Plant Foods Hum. Nutr.* **2020**, *75*, 532–539, doi:10.1007/s11130-020-00843-x.
23. Michel, P.; Olszewska, M.A. Phytochemistry and biological profile of *Gaultheria procumbens* L. and wintergreen essential oil: From traditional application to molecular mechanisms and therapeutic targets. *Int. J. Mol. Sci.* **2024**, *25*, 565, doi:10.3390/ijms25010565.
24. Michel, P.; Granica, S.; Rosińska, K.; Rojek, J.; Poraj, Ł.; Olszewska, M.A. Biological and chemical insight into *Gaultheria procumbens* fruits: a rich source of anti-inflammatory and antioxidant salicylate glycosides and procyanidins for food and functional application. *Food Funct.* **2020**, *11*, 7532–7544, doi:10.1039/d0fo01750g.
25. Michel, P.; Żbikowska, H.M.; Rudnicka, K.; Gonciarz, W.; Krupa, A.; Gajewski, A.; Machała, P.; Olszewska, M.A. Anti-inflammatory, antioxidant and photoprotective activity of standardised *Gaultheria procumbens* L. leaf, stem, and fruit extracts in UVA-irradiated human dermal fibroblasts. *J. Ethnopharmacol.* **2024**, *319*, 117219, doi:10.1016/j.jep.2023.117219.
26. Magiera, A.; Sienkiewicz, M.; Olszewska, M.A.; Kicel, A.; Michel, P. Chemical profile and antibacterial activity of essential oils from leaves and fruits of *Gaultheria procumbens* L. cultivated in Poland. *Acta Pol. Pharm. - Drug Res.* **2019**, *76*, 93–102, doi:10.32383/appdr/94245.
27. Ribnicky, D.M.; Poulev, A.; Raskin, I. The determination of salicylates in *Gaultheria procumbens* for use as a natural aspirin alternative. *J. Nutraceuticals, Funct. Med. Foods* **2003**, *4*, 39–52, doi:10.1300/J133v04n01_05.
28. Villagra, E.; Campos-Hernandez, C.; Cáceres, P.; Cabrera, G.; Bernardo, Y.; Arencibia, A.; Carrasco, B.; Caligari, P.D.S.; Pico, J.; García-Gonzales, R. Morphometric and phytochemical characterization of chaura fruits (*Gaultheria pumila*): A native Chilean berry with commercial potential. *Biol. Res.* **2014**, *47*, 1–8, doi:10.1186/0717-6287-47-26.
29. Fernández-Galleguillos, C.; Quesada-Romero, L.; Puerta, A.; Padrón, J.M.; Souza, E.; Romero-Parra, J.; Simirgiotis, M.J. UHPLC-MS chemical fingerprinting and antioxidant, antiproliferative, and enzyme inhibition potential of *Gaultheria pumila* berries. *Metabolites* **2021**, *11*, 523, doi:10.3390/metabo11080523.
30. Acuña, U.M.; Atha, D.E.; Ma, J.; Nee, M.H.; Kennelly, E.J. Antioxidant capacities of ten edible North American plants. *Phyther. Res.* **2002**, *16*, 63–65, doi:10.1002/ptr.1031.
31. Martin, P.; Wishart, J.; McDougall, G.; Brennan, R. Fruit production and polyphenol content of salal (*Gaultheria shallon* Pursh), a potential new fruit for northern maritime regions. *Fruits* **2015**, *70*, 377–383, doi:10.1051/fruits/2015040.
32. Mcdougall, G.J.; Austin, C.; Van Schayk, E.; Martin, P. Salal (*Gaultheria shallon*) and aronia (*Aronia melanocarpa*) fruits from Orkney: Phenolic content, composition and effect of wine-making. *Food Chem.* **2016**, *205*, 239–247, doi:10.1016/j.foodchem.2016.03.025.
33. Ferguson, A.; Carvalho, E.; Gourlay, G.; Walker, V.; Martens, S.; Salminen, J.P.; Constabel, C.P. Phytochemical analysis of salal berry (*Gaultheria shallon* Pursh.), a traditionally-consumed fruit from western North America with exceptionally high proanthocyanidin content. *Phytochemistry* **2018**, *147*, 203–210, doi:10.1016/j.phytochem.2018.01.002.
34. Ni, H.; Hayes, H.; Stead, D.; Liu, G.; Yang, H.; Li, H.; Raikos, V. Interaction of whey protein with polyphenols from salal fruits (*Gaultheria shallon*) and the effects on protein structure and hydrolysis pattern by Flavourzyme®. *Int. J. Food Sci. Technol.* **2020**, *55*, 1281–1288, doi:10.1111/ijfs.14394.
35. Mieres-Castro, D.; Schmeda-Hirschmann, G.; Theoduloz, C.; Rojas, A.; Piderit, D.; Jiménez-Aspee, F. Isolation and characterization of secondary metabolites from *Gaultheria tenuifolia* berries. *J. Food Sci.* **2020**, *85*, 2792–2802, doi:10.1111/1750-3841.15380.
36. Bahukhandi, A.; Pandey, A.; Sekar, C.K.; Bhatt, I. Polyphenolics, nutrients and antioxidant activity of *Gaultheria trichophylla* Royle: A high value wild edible plant of trans Himalaya. *Hortic. Int. J.* **2017**, *1*, 39–43, doi:10.15406/hij.2017.01.00007.
37. Ahmed, M.; Bose, I.; Goksen, G.; Roy, S. Himalayan sources of anthocyanins and its multifunctional applications: A review. *Foods* **2023**, *12*, 2203, doi:10.3390/foods12112203.

38. Zaman, S.; Al-Joufi, F.A.; Zafar, M.; Zahoor, M. Phytochemical, antimicrobial and cytotoxic activities of *Gaultheria trichophylla* Royle. *Appl. Sci.* **2022**, *12*, 6921, doi:10.3390/app12146921.
39. Boora, P.; Puri, R.; Angmo, D. In vitro anti-inflammatory and antioxidant properties and GC-MS based phytoconstituent screening of methanolic fruit (berry) extracts of *Gaultheria trichophylla* Royle. *Def. Life Sci. J.* **2023**, *8*, 326–333, doi:10.14429/dlsj.8.18389.

Disclaimer/Publisher's Note: The statements, opinions and data contained in all publications are solely those of the individual author(s) and contributor(s) and not of MDPI and/or the editor(s). MDPI and/or the editor(s) disclaim responsibility for any injury to people or property resulting from any ideas, methods, instructions or products referred to in the content.
